# Enzymatic Hydrolysis Methods of Insect Orthoptera Protein: A Systematic Review

**DOI:** 10.1155/ijfo/9091997

**Published:** 2026-04-24

**Authors:** Slamet Hadi Kusumah, Nurheni Sri Palupi, Azis Boing Sitanggang, Fitriya Nur Annisa Dewi, Saraswati

**Affiliations:** ^1^ Division of Food Science and Technology, Faculty of Engineering and Technology, IPB University, Bogor, 16880, Indonesia, ipb.ac.id; ^2^ Faculty of Engineering, Universitas Islam Al-Ihya, Kuningan, 45552, Indonesia; ^3^ School of Veterinary Medicine and Biomedical Sciences, IPB University, Bogor, 16880, Indonesia, ipb.ac.id

**Keywords:** biofunctional properties, enzymatic hydrolysis, insect protein, Orthoptera, protease enzymes

## Abstract

Insects from the Orthoptera order, such as grasshoppers and crickets, are increasingly receiving attention as potential alternative protein sources, especially due to their high protein content and biofunctional properties that can be developed through enzymatic hydrolysis processes. This study aimed to systematically review the enzymatic hydrolysis method of Orthoptera order insect protein and evaluate its effect on biofunctional activity, degree of hydrolysis (DH), and allergenicity. The question formulation used the PICO strategy, namely population (insects, Orthoptera order, locusts, grasshoppers, and crickets), intervention (insect processing using an enzymatic hydrolysis process), comparison (protease enzyme type, protease enzyme concentration, hydrolysis pH, hydrolysis temperature, hydrolysis duration, and drying method), and outcomes (DH, allergenicity, biofunctionality, and bioactive peptides). Sixteen articles were selected from 752 articles sourced from credible scientific journals in trusted electronic databases. The analysis showed that the Alcalase enzyme was most effective in producing bioactive peptides with antioxidant, anti‐inflammatory, and antihypertensive activities. The results of this review recommend that the enzymatic hydrolysis process in Orthoptera insects can use a 3% (w/w) Alcalase enzyme at pH 8–9 and temperature 50°C for 90 min, especially when combined with ultrasonication, which was shown to increase the DH by > 50% and the antioxidant activity significantly. Thus, the enzymatic hydrolysis process of Orthoptera insects can be an effective approach in the development of safe and high‐value functional foods and bioactive ingredients.

## 1. Introduction

Over the past decade, research on edible insects has become a hot topic worldwide. The practice of consuming insects (entomophagy) has long been an integral part of the traditional diets of various communities around the world, particularly in Asia, Africa, Latin America, and Oceania. In these regions, insects have been consumed for generations as an important source of protein, fat, vitamins, and minerals, including grasshoppers, crickets, caterpillars, ants, and beetle larvae [1]. The Orthoptera order has a higher average protein content (61% dry weight) compared to the Coleoptera (41% dry weight), Hymenoptera (46% dry weight), and Lepidoptera (45% dry weight) orders [2]. The Orthoptera order also has the advantage of being more common for consumption compared to other orders. Types of insects included in the Orthoptera order include grasshoppers and crickets, which have a protein content of 65% and 68% (dry weight), respectively. These insects are potential sources of essential amino acids, including leucine, lysine, valine, isoleucine, and threonine. They also contain fiber, healthy fatty acids, and micronutrients (such as iron, zinc, manganese, and magnesium) [3].

The acceptance of insect consumption in certain communities is inseparable from religious considerations. In some religious traditions, particularly Islam, grasshoppers are explicitly mentioned as animals that are halal (permissible) for consumption, thus providing religious legitimacy for the practice of entomophagy in Muslim communities [4]. This religious concern plays an important role in shaping people’s attitudes toward insect consumption, as halal status or religious permissibility is a determining factor in food acceptance [5]. Thus, consuming insects from the order Orthoptera does not conflict with religious perspectives, which strengthens its potential as an alternative protein source for the wider community.

Insects have tremendous potential as the food of the future. Unfamiliarity and neophobia prevent some populations from accepting insects as food [6]. Insects have a distinctive appearance when compared to beef, chicken, or others. This is a weakness of insects as food because of their unusual shape if they are only simply processed, such as fried, grilled, or boiled. A survey involving groups of insect eaters and noninsect eaters in five countries: Belgium, China, Italy, Mexico, and the United States, revealed a strong correlation between food neophobia, feelings of disgust, and desire to consume insects [7]. Based on this, insect processing engineering is needed to improve the final form of processed products so that they are acceptable to consumers.

Utilization of insects as a protein source can be done by processing them into insect protein hydrolyzates. Enzymatic hydrolysis is a method for producing protein hydrolyzates using protease enzymes [8]. This process breaks down proteins into free amino acids and short‐chain peptides known as oligopeptides [9]. Processing insects through proteolysis is also effective for producing hypoallergenic insect protein hydrolyzates [10]. Such processing is very important, as it can change the molecular weight (MW) of proteins and reduce their allergenicity. However, research on the conditions of the hydrolysis process and the effect of enzymatic hydrolysis on the allergenic properties of insect proteins, especially in the order Orthoptera, is still limited.

Several protease enzymes are selected in the process of enzymatic hydrolysis of insects, such as bromelain [11], Alcalase [12], Neutrase [13], papain [14], Flavourzyme [15], Protamex [16], and a combination of these enzymes. These enzymes have various functions, abilities, advantages, disadvantages, and mechanisms for breaking protein bonds. The choice of protease enzyme type and concentration in the hydrolysis process must be appropriate and tailored to the processing objectives. The enzymatic hydrolysis process in some insects is generally carried out at pH 7–9 with a hydrolysis temperature of 50°C. Based on this, a literature study on the use of protease enzymes in the enzymatic hydrolysis process of insects, especially in the order Orthoptera, is needed.

Insect protein hydrolyzate products also have the advantage of containing various bioactive peptides due to the proteolysis process. These bioactive peptide compounds can provide added value, especially for health [17]. For example, cricket protein hydrolyzate has shown potential as an antioxidant [18]. Bioactive peptides from cricket protein hydrolyzates have also shown the α‐amylase, α‐glucosidase, and angiotensin‐converting enzyme (ACE) inhibitory activities [19]. Locust protein hydrolyzate has been reported to have anti‐inflammatory activity [20]. This is important because it will influence consumers’ intention to purchase insect‐based products [21]. Based on the above background, this study aims to provide an overview of the hydrolysis conditions expected to produce bioactive peptides and their biofunctional activities through a systematic literature review (SLR).

## 2. Methods

The SLR stages follow the steps conducted by Kusumah et al. [22], which consists of formulating research questions, collecting research sources, assessing the quality and selection of research sources, extracting, synthesizing, and interpreting data. The formulation of questions used the PICO strategy, namely population, intervention, comparison, and outcome (Table 1). The question formulation focused on investigating the impact of insect protein hydrolysis on parameters such as hydrolysis rate, allergenicity, biofunctionality, and the presence of bioactive peptides. The quality assessment and study selection process followed the Preferred Reporting Items for Systematic Reviews and Meta‐Analyses (PRISMA) guidelines, with the PRISMA checklist provided in Supporting 1. This article also has a review protocol and is registered in PROSPERO (ID: CRD420251125106). The https://www.covidence.org software was used to facilitate the collection, screening, and extraction of study sources.

**TABLE 1:** Formulation of questions with PICO.

Criteria	Description
Population	Orthoptera order: locust, grasshopper, cricket
Intervention	Processing insects using enzymatic hydrolysis process
Comparison	Protease enzyme type, protease enzyme concentration, hydrolysis pH, hydrolysis temperature, hydrolysis duration
Outcome	Degree of hydrolysis (DH) (%), molecular weight, allergenicity, biofunctionality (IC_50_, μg/mL or mg/mL), bioactive peptides

### 2.1. Identification

The literature search was conducted from February 5 to 11, 2025, using major international electronic databases, namely Elsevier’s ScienceDirect, Scopus, PubMed, and Wiley Online Library. There are no restrictions on the year of publication in the collection of articles, as research on enzymatic hydrolysis of insect proteins (particularly within the order Orthoptera) remains a developing field with a relatively limited number of published studies. Therefore, all relevant studies were considered to ensure a comprehensive synthesis of the literature. Examples of keywords used in the literature search process are “protein hydrolysis” AND “edible insects” AND “Orthoptera.” Literature searches were also conducted by adding other more specific keywords, such as protein content, degree of hydrolysis (DH), allergenicity, or biofunctionality. A total of 16 keyword combinations (Table 2) were entered into each electronic database. This process yielded a total of 752 articles, the distribution of which can be seen in detail in Supporting 2. Only original research articles published in peer‐reviewed international journals were included, while review articles, encyclopedias, book chapters, book reviews, or other (discussion, mini‐reviews, conference abstracts, correspondence, and news) are excluded. A total of 399 original articles were selected, and the next step was to identify duplicate articles, resulting in a total of 133 articles. The process of removing duplicates is done automatically using reference software or manually. This stage aims to ensure that each publication is counted only once and to prevent bias due to data repetition from the same article.

**TABLE 2:** Keywords used in the literature search.

No.	Keywords
1	Protein hydrolysis AND edible insects
2	Protein hydrolysis AND (Orthoptera OR grasshopper OR locust OR cricket)
3	Protein hydrolysis AND edible insects AND protein content
4	Protein hydrolysis AND edible insects AND Orthoptera AND protein content
5	Protein hydrolysis AND edible insects AND degree of protein hydrolysis
6	Protein hydrolysis AND edible insects AND Orthoptera AND degree of protein hydrolysis
7	Protein hydrolysis AND edible insects AND allergen
8	Protein hydrolysis AND edible insects AND Orthoptera AND allergen
9	Protein hydrolysis AND edible insects AND allergenicity
10	Protein hydrolysis AND edible insects AND Orthoptera AND allergenicity
11	Protein hydrolysis AND edible insects AND bioactive peptides
12	Protein hydrolysis AND edible insects AND Orthoptera AND bioactive peptides
13	Protein hydrolysis AND edible insects AND biofunctionality
14	Protein hydrolysis AND edible insects AND Orthoptera AND biofunctionality
15	Protein hydrolysis AND edible insects AND (anti‐inflammatory OR antioxidants)
16	Protein hydrolysis AND edible insects AND Orthoptera AND (anti‐inflammatory OR antioxidants)

### 2.2. Screening

The remaining articles were screened based on inclusion and exclusion criteria (Table 3) found in the title and abstract. Eligible studies had to exclusively examine insects belonging to the order Orthoptera, apply enzymatic hydrolysis as the main intervention, clearly report hydrolysis conditions (enzyme type, concentration, pH, temperature, and/or time), and evaluate at least one relevant outcome, such as DH, MW distribution, allergenicity, or biofunctional activity. Studies were excluded if they focused on non‐Orthoptera insects, nonenzymatic processing methods, or lacked sufficient methodological data or results. This approach ensured that only methodologically relevant and comparable studies were included in the synthesis. This process yielded 45 relevant articles and 87 irrelevant articles. Data on the results of article selection based on title and abstract can be seen in detail in Supporting 3.

**TABLE 3:** Inclusion and exclusion criteria.

Inclusion criteria	Exclusion criteria
1. Reputable international journal 2. Has full‐text form 3. Original article/research article 4. Articles are published through the peer‐reviewed stage 5. Samples are insects in the form of whole meat or flour 6. Extraction uses the enzymatic hydrolysis method 7. There are types and concentrations of protease enzymes 8. There is an explanation of the hydrolysis temperature and time 9. There are parameters of DH and/or allergenicity and/or biofunctionality and/or bioactive peptides	1. Duplicate article 2. Book chapters 3. Review articles 4. Types of insects not included in the Orthoptera order 5. Misaligned study outcome with the review focus 6. Irrelevant intervention methods 7. Improper comparison or research design 8. Incomplete data and full text

### 2.3. Eligibility and Included

Articles that passed the title and abstract screening were then analyzed in depth through full‐text assessment. At this stage, each article was evaluated in depth to ensure its suitability with the inclusion criteria, clarity of the enzymatic hydrolysis method, availability of relevant data (e.g., DH, hydrolysis conditions, or biofunctional activity), scientific quality, and completeness of method reporting. Articles were excluded for the following reasons: types of insects not included in the Orthoptera order (*n* = 13), misaligned study outcome with the review focus (*n* = 4), irrelevant intervention methods (*n* = 4), improper comparison or research design (*n* = 3), and/or incomplete data and full text (*n* = 6). Data on the results of article selection based on full text review (inclusion and exclusion criteria) can be seen in detail in Supporting 4. Finally, 16 articles that met the inclusion criteria were selected for further analysis.

### 2.4. Data Extraction, Synthesis, and Interpretation

**FIGURE 1:**
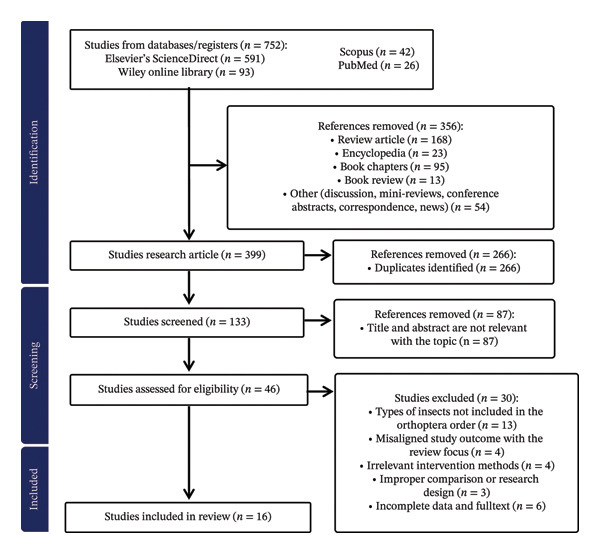
PRISMA results.

Articles that met all eligibility criteria were included in the final qualitative analysis. Data were tabulated in column format as follows: number, first author name, study, country, year of publication, insect type, hydrolysis technique, protease enzyme type, protease enzyme concentration, hydrolysis duration, hydrolysis temperature, drying method, hydrolysis rate, protein content, DH, MW, biofunctionality, and bioactive peptides. Microsoft Excel was used to organize the extracted data. The next step was to interpret the data. Figure 1 presents the results of the SLR following the PRISMA framework.

## 3. Results and Discussion

### 3.1. Insects: Orthoptera Profiles

Table 4 presents profiles of various insect species from the order Orthoptera, namely grasshoppers/locusts (*Pterophylla beltrani*, *Locusta migratoria*, *Schistocerca gregaria*, *Sphenarium purpurascens*, and *Melanoplus cinereus*) and crickets (*Gryllus assimilis*, *Gryllus bimaculatus*, *Gryllodes sigillatus*, and *Acheta domesticus*). These insects come from various countries, such as Mexico, Thailand, Brazil, Korea, Canada, Poland, Indonesia, and New Zealand. The types of raw materials used vary, ranging from whole, dried, to powdered forms. Some species such as *L. migratoria* and *A. domesticus* show high protein content, reaching 68%–71%, especially when processed into flour. Insect raw materials cultivated with attention to feed generally have more uniform nutrition and quality compared to insects in the wild [30].

**TABLE 4:** Orthoptera insect types and sample preparation for hydrolysis.

Insect name	Scientific name	Country of insect	Insect raw material shape	Protein content of raw material	Sample preparation for hydrolysis process	Sample form for hydrolysis process	References
Locust or grasshopper	*Pterophylla beltrani*	Mexico	Flour	65%	Protein extraction and lyophilized	Protein extract (powder)	[23]
	*Locusta migratoria*	Thailand	Flour	71%	Defatting and oven drying	Flour	[24, 25]
		New Zealand	Protein flour	n/a	n/a	Protein flour	[14]
	*Schistocerca gregaria*	Poland	Raw locust	n/a	Boiling/baking/raw and freeze‐drying	Flour	[18]
	*Sphenarium purpurascens*	Mexico	Raw grasshoppers	n/a	Thermally processed flour	Flour	[20]
	*Melanoplus cinereus*	Indonesia	Raw grasshoppers	n/a	n/a	Raw grasshoppers	[11]

Cricket	*Gryllus assimilis*	Brazil	n/a	n/a	Protein extraction and freeze‐drying	Protein concentrate (powder)	[12]
		Brazil	Dehydrated crickets	n/a	Protein extraction and lyophilized	Protein concentrate (powder)	[13, 19]
	*Gryllus bimaculatus*	Korea	Dried cricket	n/a	Defatting and freeze drying	Defatted flour	[16]
	*Gryllodes sigillatus*	Canada	Raw cricket	n/a	n/a	Raw cricket	[26, 27]
		Poland	Raw cricket	n/a	Boiling/baking/raw and freeze‐drying	Flour	[18]
	*Acheta domesticus*	Thailand	Powder	n/a	Protein extraction and freeze drying	Crude cricket proteins	[28]
		Thailand	Flour	70%	Defatting and oven drying	Defatted cricket powder	[29]
		Thailand	Powder	68%	n/a	Flour	[15]

### 3.2. Comparison of Sample Forms and Preparation

Sample preparation is a critical determinant of enzymatic hydrolysis efficiency, peptide profile, and the resulting biofunctional properties of insect‐derived proteins. Sample preparation methods vary depending on the initial form of the raw material and the final goal of the hydrolysis process. Processes such as defatting, oven drying or freeze drying, and protein extraction are generally performed to produce a suitable final form, such as flour, protein concentrate, or powdered protein extract. For example, *G. assimilis* and *P. beltrani* undergo protein extraction and freeze drying to obtain protein concentrate in powder form. On the other hand, some samples such as *M. cinereus* from Indonesia and *G. sigillatus* from Canada are only used in their raw form without the additional processes described.

Table 5 shows four types of sample forms used in the enzymatic hydrolysis process, namely Samples A (raw material/whole), B (flour), C (defatted flour), and D (extract, concentrate, or protein isolate). Several studies have chosen to use whole insects without first going through a protein extraction or purification process [11, 26, 27]. This approach is based on the goal of utilizing the entire nutritional components in the insect body, including proteins, lipids, and other bioactive compounds. The use of raw samples also aims to avoid additional processing steps, such as drying and fat removal, making it more efficient in terms of time and cost. Direct hydrolysis of raw materials also enables the release of a wide variety of bioactive peptides, which contribute to antioxidant activity and anti‐inflammatory and hypoallergenic potential. However, this approach has a number of limitations. The chemical composition of the raw material can vary greatly depending on the insect’s physiological conditions, life phase, and the content of nonprotein compounds such as chitin or pigments, which can compromise the efficiency of the process and the results of the analysis. The risk of microbial contamination may also be higher in raw materials, so measures such as blanching or sterilization are necessary to ensure safety.

**TABLE 5:** Reasons, advantages, and disadvantages of types of insect sample preparation.

Sample types	Reason for using this sample type	Advantages	Disadvantages	References
Sample Type A (raw material/whole)	Efficiency and simplicity of process	Lower cost and simpler and faster process	Presence of nonprotein compounds (e.g., chitin or pigments)	[26]
	Complete and natural protein source	Maintains peptide and amino acid diversity	Potential allergens (such as tropomyosin)	[27]
	Full utilization of raw materials	Minimizes waste	Composition variability	[11]

Sample Type B (flour/powder)	Standardization and consistency of composition	More consistent composition for experimental purposes or industrial‐scale production	Does not reflect the overall natural condition of the insect (e.g. peptides from specific organs may be lost)	[20]
	Commercial and practical availability	More stable and durable in storage	Potential nutrient degradation due to drying process	[14]
	Flexibility of hydrolysis process	Easy to measure and use in standardized formulas (e.g., E/S ratio)	Use of flour may decrease potential bioactivity if previous processes (e.g., autoclaving) damage the protein structure	[15]

Sample Type C (defatted flour)	Eliminates interference from fat	Cleaner and more homogeneous hydrolyzate	Use of organic solvents such as hexane	[24]
	Improves product quality and stability	Improved shelf life and oxidative stability	Defatting step adds steps and costs in processing	[25]
	Retains functional properties	Improved effectiveness of protein hydrolysis	Potential loss of fat‐soluble bioactive compounds	[29]

Sample Type D (extract/crude/concentrate/protein isolate)	Higher and more homogeneous protein content	High hydrolysis efficiency	Additional extraction process	[23]
	Minimizes the influence of nonprotein compounds	More controlled peptide profile	Possible loss of other functional compounds	[12, 19]
	Adapts to the needs of advanced applications (nutraceutical or pharmaceutical)	Higher bioactivity potential	Not environmentally friendly if using chemical solvents	[13, 28]

In contrast, studies by Purschke et al. [14], Zielińska et al. [18], Grossmann et al. [15], and Villaseñor et al. [20] used insect flour as starting materials for hydrolysis. Insect flour is generally obtained through a process of drying, grinding, and sometimes defatting, which results in a more stable, homogeneous, and easy‐to‐handle material. This approach is more suitable for applicative and industrial purposes, as it allows for process standardization and more consistent results. The use of flour facilitates control over substrate protein concentration and hydrolysis process parameters such as enzyme‐substrate ratio and incubation time. Another advantage of flour is the lack of interference from nonprotein compounds, as well as increased efficiency in the extraction and analysis of bioactive compounds. Insect meal has the disadvantage of requiring thermal treatment, which can potentially cause protein denaturation or degradation of active peptides. Some natural functional components of insects can also be lost during the defatting or drying process, which can affect the biological activity of the final product.

The use of defatted materials has a strong scientific basis and significant technological benefits. One of the main reasons for defatting is to increase the efficiency of enzymatic hydrolysis. Fats present in raw materials can inhibit enzyme access to protein substrates because they form physical and chemical barriers and interfere with protein dispersion in liquid media. By removing fat through solvents such as n‐hexane, researchers were able to increase the availability of proteins to enzymes, resulting in a higher DH and a more uniform peptide profile [24]. Defatting also plays an important role in improving the sensory quality and oxidative stability of the final product. High fat content can be a substrate for lipid oxidation reactions that result in rancidity and reduced food quality [25]. Therefore, defatting provides advantages in terms of improved shelf life and organoleptic quality of the resulting protein hydrolyzate. Overall, the defatting step before protein hydrolysis represents an important procedure in obtaining insect protein hydrolyzates with high functional quality and bioactivity, which are highly beneficial for applications as functional foods and bio‐preservatives.

One important approach in the process of insect protein hydrolysis is the use of raw materials in the form of extracts, concentrates, or protein isolates, rather than whole forms or flour. The use of these purer forms is based on several technical and functional considerations. First, the higher and more homogeneous protein content in protein concentrates or isolates allows the hydrolysis process to take place more efficiently. Proteolytic enzymes can work more specifically and effectively on substrates that are not blocked by nonprotein compounds such as chitin, lipids, or complex carbohydrates [12, 23]. Secondly, materials in the form of concentrates or isolates are reported to provide better control over protein composition and structure, which is crucial for producing bioactive peptides with optimal molecular size and amino acid sequence [13]. Several studies have shown that low MW peptides (< 3 kDa) have higher biological activities, including antioxidant, antihypertensive, and antidiabetic activities [13, 28]. However, this approach is not free from limitations. Protein extraction and purification processes require additional steps such as alkali extraction and acid precipitation (AEAP), salting‐in, salting‐out, ultrasonication, or ultrafiltration [22]. On the other hand, the use of chemical solvents such as NaOH or HCl in these processes also raises environmental issues that need to be considered [13, 23].

Based on the comparative evaluation of sample forms used in enzymatic hydrolysis of insect proteins, defatted insect flour is recommended as the most appropriate substrate. This form provides an optimal balance between hydrolysis efficiency, process controllability, and functional performance. The removal of lipids enhances enzyme accessibility to protein substrates, resulting in a higher and more consistent DH as well as a more uniform peptide profile. Therefore, defatted insect flour represents a pragmatic and scalable option that supports both high biofunctional quality and the industrial feasibility of insect protein hydrolyzates.

### 3.3. Modifications in the Enzymatic Hydrolysis Process

**FIGURE 2:**
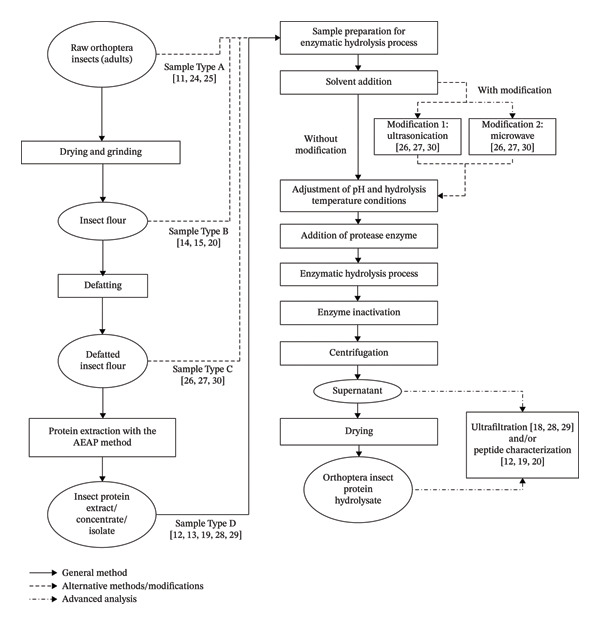
Flow diagram of enzymatic hydrolysis of Orthoptera insect protein.

Figure 2 Schematic overview of sample preparation and enzymatic hydrolysis strategies applied to Orthoptera insect proteins in the reviewed studies. Raw adult Orthoptera insects (Sample Type A) were processed into different sample types, including insect flour (Sample Type B), defatted insect flour (Sample Type C), and protein extract or isolate obtained via the AEAP method (Sample Types D). Enzymatic hydrolysis was performed either without modification or with physical pretreatments, including ultrasonication (Modification 1) and microwave treatment (Modification 2), prior to protease addition. Some advantages and disadvantages of each modification technique in the enzymatic hydrolysis process are presented in Table 6.

**TABLE 6:** Advantages and disadvantages of the additional method to hydrolysis process.

Additional method	Reason for use	Advantages	Disadvantages	References
Ultrasonication	Low thermal	Improves protein hydrolysis efficiency	Strict temperature control	[29]
	Increase antioxidant and antimicrobial activity	Improves storage stability of food products	Equipment cost	[24]
	Potential to reduce allergenicity	Preserves the structure of bioactive compounds	Complex process parameters	[25]

Microwave	Easy to adopt industrially	Efficient and fast	Uneven heat distribution	[29]
	Thermally alters protein structure	Enhances the biological activity of hydrolyzates	Potential protein degradation	[24]
	Potential to reduce allergenicity	Changes the conformation of protein allergens, such as tropomyosin	Lack of microcontrol at the molecular level	[25]

Ultrafiltration	Selective by size	Increase biopeptide activity	Possibility of small peptide loss	[23]
	Can increase the specificity of functional activity evaluation	Maintaining peptide stability	Cost and equipment	[28]
	To prepare fractions for nutraceutical products	Selected fractions can be further analyzed for amino acid sequencing and bioactivity studies	Membrane fouling	[18]

#### 3.3.1. Ultrasonication Treatment

Ultrasonication is a nonthermal technology that utilizes high‐frequency sound waves (typically above 20 kHz) to generate cavitation phenomena in the solution. This cavitation creates frictional forces and microcollisions, which can lead to the denaturation and disintegration of protein structures. In all three reviewed studies, ultrasonication was applied prior to the hydrolysis of proteins from locusts (*L. migratoria*) and crickets (*Acheta domestica*) using the Alcalase enzyme. This treatment was shown to cause protein conformational changes (unfolding), increase surface hydrophobicity, and disrupt the secondary and tertiary structure of proteins [24, 25, 29]. Ultrasonication is particularly relevant for chitin‐containing materials such as insects by disrupting their cell walls.

Comparative analysis across the reviewed studies indicates that the application of ultrasonication pretreatment prior to enzymatic hydrolysis consistently enhances both DH and antioxidant activity of Orthoptera protein hydrolyzates compared to nonultrasonicated controls. For example, studies on *G. sigillatus* and *L. migratoria* proteins reported that ultrasonication significantly increased DH by more than 50% under identical Alcalase hydrolysis conditions due to ultrasound‐induced protein unfolding and disruption of intermolecular interactions, which improved enzyme accessibility to peptide bonds [24, 25]. This enhanced hydrolysis was accompanied by markedly higher antioxidant activity, particularly in low‐molecular‐weight peptide fractions (< 3 kDa) generated after ultrasonication‐assisted hydrolysis [24]. In contrast, studies employing conventional enzymatic hydrolysis without ultrasonication generally reported lower DH values and reduced radical scavenging capacity, even when using similar enzyme types and reaction conditions [14].

#### 3.3.2. Microwave Treatment

Microwave treatment was shown to provide significant benefits in improving the quality and bioactive potential of insect protein hydrolyzates. Based on the research results of tropomyosin [25], microwave treatment of locust protein flour before the hydrolysis process with the Alcalase enzyme was able to significantly increase antioxidant activity, namely, the 1,1‐diphenyl‐2‐picrylhydrazyl (DPPH) value increased by about 20.9%, 2,2′‐azino‐bis(3‐ethylbenzothiazoline‐6‐sulfonic acid) (ABTS) increased by 19.6%, and ferric reducing antioxidant power (FRAP) increased by 20.3% compared to the untreated sample. Antimicrobial activity data also increased, as indicated by a decrease in the minimum inhibitory concentration (MIC) value against *Staphylococcus aureus* from 40 mg/mL to 35 mg/mL (12.5% increase in efficiency). In the application of processed products such as meat emulsion, the addition of microwave‐treated hydrolyzates was able to reduce thiobarbituric acid reactive substances (TBARS) values by 25% during storage, indicating a slowdown in lipid oxidation. This improvement was attributed to protein conformational changes due to thermal treatment, which increased enzyme accessibility to hydrolysis sites, resulting in bioactive peptides in higher amounts and effectiveness.

The use of a microwave is also associated with reduced allergenicity due to changes in the conformational structure of certain proteins such as tropomyosin [25]. This technology may also contribute to the safe consumption of insect protein, particularly for individuals sensitive to allergens. One limitation of microwave use is the risk of degradation of bioactive proteins or peptides due to high temperatures, especially if processing time is not controlled properly. Uneven heat distribution in conventional microwave systems can lead to the formation of hot spots, which can affect the uniformity of hydrolysis results. In contrast to low‐thermal technologies such as ultrasonication that work via mechanical cavitation, microwave relies on heat transfer, which has intrinsic limitations in the control of protein microstructure [29].

### 3.4. Types of Protease Enzymes in Orthoptera Insect Hydrolysis

Based on Table 7, seven types of proteolytic enzymes are obtained, which are commonly used for insect protein hydrolysis. One of the most widely used enzymes is Alcalase, a protease from *Bacillus licheniformis* that is available in both liquid and powder form and originates from various countries such as Germany, the United States, Denmark, and Brazil. This enzyme was chosen because it has high endoprotease activity, so it can produce peptides with antihypertensive activity through inhibition of ACE [12, 28]. For example, research by Summart et al. [28] used house cricket (*A. domesticus*) powder as the protein feedstock, which was then hydrolyzed using 5% (v/w) Alcalase enzyme at pH 9 and 50°C for 3 h. This process yielded the peptide fraction that showed the highest ACE inhibitory activity of 57.93%, but this was the result of further fractionation via chromatography, not of the entire hydrolyzate. This means that only a small fraction of the hydrolyzed product has high bioactivity.

**TABLE 7:** Protease enzyme profile (single enzyme).

Name of protease enzymes	Enzyme form	Country of origin of enzyme	Biological sources (animal/plant/microbe)	Enzyme activity	Main reasons this enzyme was chosen	References
Alcalase	Liquid	Germany	*Bacillus licheniformis*	18000 U/mL	Because it can produce a lot of peptides in a short time	[12]
	Powder	United States	*Bacillus licheniformis*	2.4 AU/g	Because of its ability to produce protein hydrolyzates consisting of low molecular weight peptides (less than 1 kDa) that have high bioactive activity, especially in terms of antioxidant activity and inhibition of ACE	[28]
	Powder	United States	*Bacillus licheniformis*	2.4 AU/g	Alcalase has been reported to be effective for chitin‐containing materials such as insect protein, making it suitable for hydrolyzing proteins from crickets	[29]
	Liquid	United States	*Bacillus licheniformis*	2.4 AU/g	The authors stated that Alcalase can produce hydrolyzed proteins with high antimicrobial and antioxidant activities	[25]
	Liquid	Denmark	*Bacillus licheniformis*	2.4 AU/g	Often used in the food industry because it has high activity and is stable at moderate pH and temperature (pH 8, 50°C)	[14]
	Liquid	Denmark	*Bacillus licheniformis*	2.4 AU/g	Capable of degrading insect proteins into small peptides very effectively	[15]
	Liquid	United States	*Bacillus licheniformis*	2.4 AU/g	Alcalase is particularly effective for chitin‐containing materials such as insects and has been shown to produce hydrolyzates with high antioxidant and antimicrobial potential	[24]
	Powder	United States	*Bacillus licheniformis*	2.4 AU/g	Alcalase is effective in producing bioactive peptides from various protein sources, including insects, and is able to reduce allergenicity.	[27]

Flavourzyme	Liquid	Germany	*Aspergillus oryzae*	6000 U/mL	Its presence is very important because it can produce short peptides from the ends of proteins, which contribute greatly to antidiabetic activity (α‐amylase and α‐glucosidase inhibitors)	[12]
	Liquid	Brazil	*Aspergillus oryzae*	n/a	Having endopeptidase and exopeptidase activity, which allows the release of free amino acids, provides additional benefits to the bioactive properties of hydrolyzed peptides	[19]
	Liquid	Denmark	*Aspergillus oryzae*	n/a	Flavourzyme is an enzyme that is widely known in the food industry and has been used in various previous studies	[15]
	Liquid	Denmark	*Aspergillus oryzae*	1100 LAPU/g	Suitable as a second‐stage enzyme in the two‐step hydrolysis process because it complements the breakdown by endoproteases	[14]

Neutrase	Liquid	Germany	*Bacillus amyloliquefaciens*	200,000 U/mL	To cut peptide bonds inside the chain (endopeptidase)	[12]
	Liquid	Denmark	*Bacillus amyloliquefaciens*	0.8 AU‐N/g	Neutrase is used as a reference in the exploratory stage but is not the primary enzyme selected for further applications	[16]
	Liquid	Brazil	*Bacillus amyloliquefaciens*	n/a	Neutrase is a neutral endoprotease from *Bacillus amyloliquefaciens* that has an affinity for hydrophobic amino acids	[13]

Papain	Powder	United States	Papaya latex	10 U/mg protein	It has been commonly used in the food industry to produce protein hydrolyzates	[14]

Bromelain	Powder	n/a	Pinecaps	n/a	Bromelain was chosen because it does not cause a bitter taste, unlike other protease enzymes such as papain, which can leave a bitter taste in the hydrolyzate results	[11]

Protamex	n/a	Denmark	n/a	n/a	Protamex is used to evaluate its ability to hydrolyze insect proteins by cleaving peptide bonds in the interior of the protein chain (endopeptidase)	[16]

Protease A “Amano” 2SD	Powder	Japan	n/a	n/a	Selected based on the results of sensory pre‐experiments on 12 types of enzyme preparations. The results showed that the hydrolyzate produced with Protease A had the most balanced, rounded, and umami‐like flavor profile	[15]

The second most popular enzyme that the author is interested in is Flavourzyme. This enzyme has activity as an endo‐ and exoprotease, which allows the breaking of peptide bonds both in the interior of the protein chain and at its terminal end [13, 19]. In a study conducted by de Matos et al. [12], the Flavourzyme enzyme was chosen to hydrolyze cricket protein concentrate due to its strong exopeptidase (aminopeptidase) activity; furthermore, the hydrolyzate produced by this enzyme contained short peptides that had antidiabetic activities, such as inhibition of α‐amylase and α‐glucosidase.

Another enzyme is Neutrase from *Bacillus amyloliquefaciens*, which acts as a neutral endoprotease enzyme. Neutrase is known to have an affinity for hydrophobic amino acids and can produce a good distribution of peptide MWs [13]. Proteolytic enzymes from plant sources are also used, such as papain from papaya latex and bromelain from pineapple stems. Papain is often used in the food industry to produce protein hydrolyzates [14], while bromelain was chosen because it does not impart a bitter taste to the hydrolysis results, in contrast to papain, which can leave a bitter aftertaste [11]. Other enzymes used in the exploratory study included Protamex from Denmark, which functions as an endoprotease in insect protein hydrolysis [16], as well as Protease A “Amano” 2SD from Japan, which was selected based on sensory tests from 12 types of enzymes, and it produced the most balanced and umami hydrolyzate flavor [15].

### 3.5. The Effect of Enzyme Type and Concentration on the DH

The DH is an important parameter in assessing the extent to which a protein has been broken down into peptides and amino acids through enzymatic processes. DH is defined as the percentage of the number of peptide bonds that have been cleaved compared to the total number of peptide bonds in an intact protein molecule. The study report data on the DH of Orthoptera insects can be seen in Table 8.

**TABLE 8:** Degree of hydrolysis and molecular weight of protein hydrolyzate.

Insect type	Hydrolysis process conditions		DH (%)	Molecular weight (kDa)		References
	Enzyme type and concentration (E/S)	pH, temperature, and duration of hydrolysis		Before hydrolysis	After hydrolysis	
Locust	Neutrase 0.5% + Flavourzyme 1%	8; 50°C; 60 min	5.1–28.5	10–75	10–15	[14]
Locust	α‐Amylase, pepsin, pancreatin, and bile extract solution	n/a; 37°C; n/a	n/a	29–116	6.5–97.2	[18]
Grasshopper	Pepsin + pancreatin	Pepsin pH 2, pancreatin pH 7.5; 37°C; 120 min	n/a	50–150	37–125	[20]
Grasshopper	Bromelain 4%–6%	7; 55°C; 420 min	44.65–51.33	n/a	n/a	[11]
Cricket	Neutrase 0.5% + Flavourzyme 0.5%	n/a; 50°C; 120 min	n/a	26–94	< 14	[12]
Cricket	Combination by Flavourzyme, Alcalase, and/or Neutrase	7; 50°C; 120 min	n/a	> 67	3.5–30	[19]
Cricket	Combination by Flavourzyme, Alcalase, and/or Neutrase	7; 50°C; 120 min	n/a	43–94	< 14	[13]
Cricket	Flavourzyme 30 U with Alcalase 12 mU/g protein	n/a; 55°C; 240 min	n/a	50–150	< 14	[16]
Cricket	Alcalase 0.5%–3%	8; 50°C; 90 min	26.1–52.4	14.4–212	< 14.4	[26]
Cricket	α‐Amylase, pepsin, and pancreatin	n/a; 37°C; n/a	n/a	29–116	6.5–97.2	[18]
Cricket	Alcalase 3%	8; 50°C; 80 min	15–85	37	< 37	[27]
Cricket	Flavourzyme 2% or Alcalase 2%	n/a; n/a; 120 min	33–46	n/a	n/a	[15]

A consistent trend emerges when comparing enzyme types and processing conditions. Studies employing Alcalase under neutral to alkaline conditions (pH 8–9) and temperatures around 50°C–55°C generally reported higher DH values than those using plant‐derived proteases such as papain or bromelain. For example, Hall et al. [26] reported a DH of 52.4% for *G. sigillatus* protein hydrolyzed using 3% Alcalase at pH 8 and 50°C for 90 min. In contrast, bromelain‐treated *M. cinereus* protein achieved a protein digestibility of approximately 51% only at the highest enzyme concentration, without direct DH quantification [11]. This difference can be attributed to the strong endoprotease activity of Alcalase, which preferentially cleaves internal peptide bonds and rapidly generates free amino groups detectable by trinitrobenzenesulfonic acid (TNBS) or ortho‐phthalaldehyde (OPA) assays [14, 27].

Methodological differences in DH determination also contribute to variability among studies. The TNBS method, which quantifies free amino groups at 420 nm, is widely used and sensitive for assessing peptide bond cleavage [14, 26]. Alternatively, the OPA method enables rapid, high‐throughput analysis at 340 nm and is commonly applied in studies focusing on flavor development and functional properties [15]. Although both methods quantify liberated amino groups, differences in reagent specificity, calibration, and reference standards can lead to systematic variation in reported DH values. Consequently, cross‐study comparisons should be interpreted with caution unless standardized analytical protocols are adopted [15, 27].

The selection of enzyme concentrations for insect protein hydrolysis should not be solely focused on achieving a high DH value but rather on the desired peptide bioactivity. Analysis of studies in Orthoptera shows that increasing enzyme concentrations does not always correlate linearly with increased biological activity, so the feasibility of their use needs to be evaluated functionally, not just technically. Several studies have shown that low‐ to moderate‐enzyme concentrations (0.5%–3%) are capable of producing low MW peptide fractions (< 3–10 kDa) that exhibit significant bioactive activity, particularly antioxidant activity [24, 25]. Conversely, the use of high enzyme concentrations (3%–6%) can significantly increase DH, but this does not always result in a comparable increase in bioactivity [28]. Excessive hydrolysis can potentially produce very short peptides or free amino acids, which in some cases actually reduces specific antioxidant activity, increases bitterness, and reduces peptide stability [29]. Therefore, although biochemically effective, high enzyme concentrations are often less feasible if the primary goal is the production of functional peptides rather than maximum protein degradation.

### 3.6. Comparison of Single Enzymes and Enzyme Combinations

Several research reports have used a combination of enzymes in the hydrolysis of insect proteins (Table 9). The use of combined protease systems is frequently proposed as a strategy to enhance protein hydrolysis efficiency by exploiting the complementary actions of endo‐ and exopeptidases. However, a critical assessment of Orthoptera protein hydrolysis studies indicates that the necessity of enzyme combinations is highly dependent on the intended outcome of hydrolysis and not universally justified.

**TABLE 9:** Protease enzyme combination in enzymatic hydrolysis process.

Enzyme combination	Reason for enzyme selection	Positive effect	Negative effect	References
*Double enzyme:*				
Flavourzyme + Alcalase	Enhances peptide cleavage and release of bioactive peptides	Solubility increased from 10% to 22% to around 55% at pH 9 after 120 min of hydrolysis	ACE activity lost after digestion	[14, 16, 19]
Flavourzyme + Neutrase	Combination of endo‐ and exopeptidase for hydrolysis efficiency	Higher solubility than single enzyme	Decreased foamability at neutral to alkaline pH	[14, 19]
Flavourzyme + Papain	Protease combination for complete degradation of peptides into small size	High‐oil binding capacity (2.33 g/g)	Low degree of hydrolysis (21.1%)	[14]

*Triple enzyme:*				
Alcalase + Neutrase + Flavourzyme	Proteolytic synergy for maximum hydrolysis	Highest DH (41.6%)	Significant decrease in foamability at pH 7–9	[14]
Alcalase + Papain + Flavourzyme	Efficiency of protein degradation and improvement of functional properties (emulsion, foam, solubility)	More water‐soluble peptide fragments	Drastically decreased foam stability at pH 3 and 5 (< 10%)	[14]
Neutrase + Papain + Flavourzyme	Optimization of protein degradation and improvement of functional properties	High solubility at pH 9 (up to 55%)	Drastic decrease in foam stability at pH 3 and 5	[14]

*Quadruple enzyme:*				
Alcalase + Neutrase + Papain + Flavourzyme	To see synergistic effects and improved functional properties	Solubility increased by 55% compared to control (10%–22%)	Not able to maintain foam at pH 5	[14]

*Digestive enzymes:*				
Pepsin + pancreatin	Mimics natural digestion process	Low IC_50_ against DPPH, ABTS, inducible nitric oxide synthase (iNOS), COX‐2	Some peptides are inactive or too small to be effective	[20]
Porcine pepsin + bovine trypsin + chymotrypsin	Realistic simulation of in vitro digestion	ACE, antidiabetic, antioxidant activity	Fraction < 3 kDa has the lowest ACE inhibitor activity	[23]
α‐Amylase + pepsin + pancreatin	Realistic evaluation of complete digestion	High antioxidant and anti‐inflammatory activity (IC50: LOX 3.13; COX‐2 5.05 μg/mL)	After dialysis and in vitro absorption, peptide content decreased	[18]

Single broad‐specificity endoproteases, particularly Alcalase, have repeatedly demonstrated the ability to achieve high DH and substantial MW reduction without requiring additional enzymes. For example, Alcalase applied to cricket proteins at concentrations of 0.5%–3% produced DH values up to 52.4% [26], while Alcalase at 3% achieved DH values approaching 85% under similar pH and temperature conditions [27]. These results suggest that, for applications primarily targeting extensive peptide bond cleavage or high DH, a single, highly active endoprotease may be sufficient, rendering enzyme combinations redundant from both a technical and economic perspective.

In contrast, studies employing combined enzyme systems, such as Neutrase with Flavourzyme or combinations involving Alcalase, Neutrase, and Flavourzyme, often did not report markedly higher DH values than those achieved using single enzymes [13]. Instead, the main benefit of enzyme combinations was observed in the modulation of peptide size distribution, with posthydrolysis MWs frequently reduced to < 14–15 kDa. This indicates that exopeptidases such as Flavourzyme primarily refine peptide termini rather than substantially increasing the overall extent of hydrolysis. Consequently, enzyme combinations appear more effective for tailoring peptide profiles than for maximizing DH.

The combination of Flavourzyme and Neutrase enzymes produced a protein hydrolyzate from *G. assimilis* that had relatively stable antidiabetic activity after simulated digestion. The activity was shown by the ability to inhibit the α‐amylase enzyme by 47.87% (at a concentration of 5 mg/mL) and α‐glucosidase by 12.73% (at a concentration of 2.5 mg/mL) after in vitro digestion treatment [19]. A combination of three types of enzymes is used to synergistically optimize protein degradation. The combination of Alcalase, Neutrase, and Flavourzyme was shown to produce the highest DH (41.6%) [14]. Intensive hydrolysis, especially with a combination of four enzymes, namely Alcalase, Neutrase, Papain, and Flavourzyme, causes very high protein degradation, which produces short peptides [14].

From a structural perspective, MW analysis provides essential insight into the extent of protein breakdown following enzymatic hydrolysis. Intact insect proteins typically exhibit high MWs (approximately 50–200 kDa), which are progressively reduced through peptide bond cleavage into smaller peptides (< 10 kDa). Techniques such as SDS–PAGE are commonly employed to visualize these changes at a macro level. For example, enzymatic hydrolysis of *L. migratoria* proteins resulted in the disappearance of protein bands in the 25–75 kDa range and the emergence of new bands between 10 and 15 kDa, confirming effective protein degradation [14]. Comparable findings were reported for *G. assimilis*, where hydrolysis—particularly using Flavourzyme or enzyme combinations—produced predominantly low‐MW polypeptides (< 14 kDa), suggesting enhanced potential for releasing antioxidant peptides [13].

Importantly, MW reduction is also closely linked to allergenicity modulation. Hall et al. [27] demonstrated that extensive Alcalase‐mediated hydrolysis of cricket protein (60%–85% DH) significantly reduced or eliminated IgE binding to tropomyosin (37 kDa) in shrimp‐allergic human sera, indicating that enzymatic breakdown of allergenic epitopes is associated with reduced IgE reactivity. Collectively, cross‐study comparisons indicate that while SDS–PAGE effectively captures overall protein degradation, ultrafiltration combined with peptide fractionation reveals functionally relevant low‐MW peptides (< 3–5 kDa) that are more directly associated with enhanced bioactivity and reduced allergenic potential [18, 23, 27].

From a critical standpoint, the routine use of enzyme combinations may also introduce unnecessary complexity. Multi‐enzyme systems require additional optimization steps, including sequential or simultaneous enzyme addition, pH adjustment, and control of reaction kinetics. Yet, many studies do not clearly justify why a combined approach was selected, nor do they compare it systematically against single‐enzyme controls under equivalent conditions. This omission weakens the argument that enzyme combinations are inherently superior. Overall, evidence from Orthoptera protein hydrolysis studies suggests that the use of combined protease systems is not intrinsically necessary. Single‐enzyme systems, particularly those based on Alcalase, are often sufficient to achieve high DH and significant protein breakdown. Enzyme combinations should therefore be considered a target‐specific tool, justified only when precise control over peptide size distribution or terminal amino acid composition is required.

### 3.7. The Effects of Digestion Compared to Hydrolysis With Commercial Proteases

Simulation of human digestive enzymes is done to mimic the natural process in vitro. The combination of enzymes such as porcine pepsin, bovine trypsin, and chymotrypsin was able to produce peptides with antihypertensive, antidiabetic, and antioxidant activities [23]. The study actually showed that the ACE inhibitor activity with the peptide fraction < 3 kDa (34.3% inhibition) was lower than the fraction > 10 kDa (67.39% inhibition). This suggests that in this case, ultrafiltration is not necessary to enhance the ACE inhibitory response. The combination of pepsin and pancreatin produced peptides with strong antioxidant activity based on DPPH (IC_50_ = 0.78 ± 0.01 mg/mL) and ABTS (IC_50_ = 0.63 ± 0.01 mg/mL) [20]. Meanwhile, a more complete combination including α‐amylase, pepsin, pancreatin, and bile extract produced peptides from the locust *S. gregaria* that showed the highest anti‐inflammatory activities (IC_50_ LOX: 3.13 μg/mL; COX‐2: 5.05 μg/mL) [18], although the peptide content decreased after in vitro absorption. Bile extract containing bile salts plays a role in emulsifying the relatively high lipid fraction in insects, thereby opening up the food matrix structure and making proteins more accessible to proteolytic enzymes. It can be argued that Alcalase‐based hydrolysis consistently produced higher degrees of hydrolysis and stronger antioxidant activity compared to plant‐derived proteases such as papain or bromelain. In contrast, studies employing simulated gastrointestinal digestion tended to generate peptides with broader MW distributions and variable bioactivities, suggesting that enzyme specificity plays a critical role in determining functional outcomes.

### 3.8. Ultrafiltration and Peptide Characterization

Ultrafiltration is widely applied to fractionate insect protein hydrolyzates into peptide groups based on MW, commonly producing fractions such as < 3, < 10, and > 10 kDa. This approach is grounded in extensive evidence that low‐molecular‐weight peptides, particularly those below 3–5 kDa, tend to exhibit enhanced biological activities, including antioxidant capacity and ACE inhibition, which are closely associated with blood pressure regulation [23]. Functionally, ultrafiltered peptide fractions derived from insect proteins have demonstrated diverse bioactivities, such as free radical scavenging, suppression of intracellular reactive oxygen species (ROS), and upregulation of endogenous antioxidant defense systems, including superoxide dismutase (SOD), catalase (CAT), glutathione synthetase (GSR), and glutathione peroxidase (GPx).

While MW distribution provides a useful preliminary indication of protein breakdown, it offers limited insight into the structural determinants of peptide bioactivity. Consequently, peptide characterization at the sequence level using mass spectrometry (MS)‐based techniques has become increasingly important in studies of enzymatic hydrolysis. Across the reviewed literature, liquid chromatography–tandem mass spectrometry (LC–MS/MS), including high‐resolution MS/MS (HRMS/MS), was the most commonly applied approach for peptide identification. Several studies employed MS‐based workflows to identify specific bioactive peptide sequences following ultrafiltration and chromatographic fractionation (Table 10). Across the reviewed studies, peptide sequencing and complementary in silico analyses enabled the identification of anti‐inflammatory candidates such as GPPGPAGV and KPTVGVVTY that interact with COX‐2 and iNOS pathways, while other hydrolyzates yielded multifunctional peptides, including AGDDAPR and YPLDL, associated with the inhibition of α‐amylase, α‐glucosidase, and ACE, supporting antidiabetic and antihypertensive potential [12, 20]. Further refinement through chromatographic fractionation coupled with LC–MS/MS uncovered short tetrapeptides (e.g., FVEG and FYDQ) exhibiting strong cellular antioxidant capacity and enzyme‐binding affinity, demonstrating how advanced MS workflows enhance mechanistic interpretation of peptide function [28]. Importantly, untargeted proteomic profiling after simulated gastrointestinal digestion confirmed that low‐molecular‐weight fractions (< 3 kDa) still contain sequence‐identifiable peptides such as LPPLP with predicted multifunctional bioactivity, emphasizing that biological relevance depends on peptide identity and stability rather than size distribution alone [19].

**TABLE 10:** MS‐based peptide characterization of Orthoptera insect protein hydrolyzates.

Insect type	MS platform/workflow	Reported key peptides or motifs	Main bioactivities reported	References
Cricket (*G. assimilis*)	Proteomic/peptidomic identification of bioactive peptides after enzymatic hydrolysis	AGDDAPR; YPLDL	Antidiabetic (α‐amylase and α‐glucosidase inhibition) and antihypertensive (ACE inhibition) activities	[12]
Cricket (*A. domesticus*)	LC–MS/MS after chromatographic fractionation	FVEG; FYDQ	Cellular antioxidant activity and ACE inhibition with strong binding affinity to target enzymes	[28]
Cricket (*G. assimilis*)	Untargeted MS‐based proteomics of < 3 kDa peptide fraction	LPPLP (predicted bioactive in silico)	Antioxidant, antidiabetic, and antihypertensive activities affected by simulated digestion	[19].
Grasshopper (*S. purpurascens*)	Peptide sequencing followed by molecular docking analysis	GPPGPAGV; KPTVGVVTY	Anti‐inflammatory (COX‐2 and iNOS inhibition) and antioxidant activities	[20]

However, a critical synthesis reveals that not all studies reporting enhanced bioactivity performed sequence‐level peptide identification. In several cases, conclusions regarding bioactivity were inferred solely from MW fractions (e.g., < 3–3.5 kDa) without MS‐based confirmation of peptide composition. This highlights an important limitation in the current literature, as similar MW ranges may contain peptides with markedly different sequences and functional properties. Therefore, the integration of MS‐based peptide identification is essential for establishing robust structure–function relationships and for validating claims of bioactive peptide generation.

### 3.9. Impact of Enzymatic Hydrolysis on the Biofunctionality of Orthoptera Protein Hhydrolyzates

#### 3.9.1. Antioxidant Activity

The antioxidant profiles of various insect species were evaluated using DPPH, ABTS, and FRAP methods (Table 11). The available data show a very high methodological variability. Notable differences are seen in the type of enzyme used (such as Alcalase, Pepsin, Flavourzyme, and others); enzyme concentration, pH, temperature, hydrolysis duration, as well as additional treatments such as ultrasonication or ultrafiltration. Furthermore, the reporting of antioxidant activity values is also inconsistent; the units used vary widely, ranging from percent inhibition and IC_50_ to μmol or mg of Trolox per gram of protein or peptide. These variations make direct comparisons between studies difficult, as the results obtained are not derived from uniform experimental conditions.

**TABLE 11:** The antioxidant activity of Orthoptera insect protein hydrolyzates.

Insect types	Antioxidant activity	Hydrolysis process conditions (enzyme type and concentration E/S; pH; T; t; modification)	References
*Method: DPPH (2,2-diphenyl-1-picrylhydrazyl):*			
Locust (*L. migratoria*)	82.00 ± 0.95% inhibition at a concentration of 50 mg/mL	Alcalase; 3% w/w; pH 8; 50°C; 90 min; ultrasonication	[24, 25]
Grasshopper (*S. purpurascens*)	IC_50_ = 0.78 ± 0.01 mg/mL	Pepsin (pH 2) + pancreatin (pH 7.5); 1:20 (w/w); 37°C; 2 + 2 h	[20]
Cricket (*G. sigillatus*)	IC_50_ = 10.9 μg/mL	α‐Amylase, pepsin, pancreatin, and bile extract solution; n/a; pH 7; 37°C; n/a	[18]
Cricket (*G. assimilis*)	47.74 ± 0.41 μmol TE/g protein	Flavourzyme; 100 U/mL; pH 7; 50°C; 2 h	[19]
Cricket (*G. assimilis*)	IC_50_ = 455 μg/mL	Flavourzyme; 100 U/mL; pH 7; 50°C; 2 h	[13]
Cricket (*G. sigillatus*)	0.21 mM TE/mg (after digestion)	Alcalase; 3% (w/w); pH 8; 50°C; 40 min	[27]
Cricket (*A. domesticus*)	393.6 ± 3.7 mg Trolox/g peptide	Alcalase; 5%; pH 9; 50°C; 3 h	[28]
Cricket (*A. domesticus*)	60.7 ± 1.3% inhibition at a concentration of 50 mg/mL	Alcalase; 3% (w/w); pH 8; 50°C; 90 min; ultrasonication	[29]

*Method: ABTS (2,2′-azino-bis(3-ethylbenzothiazoline-6-sulfonic acid)):*			
Locust (*P. beltrani*)	554.01 μM TE/mg protein (*F *< 3 kDa)	Pepsin (pH 2) + trypsin (pH 7.8) + chymotrypsin; 1:10 (w/w); 37°C; 5 h; ultrafiltration	[23]
Locust (*L. migratoria*)	88.00 ± 1.20% inhibition at a concentration of 50 mg/mL	Alcalase; 3% (w/w); pH 8; 50°C; 90 min; ultrasonication	[24, 25]
Grasshopper (*S. purpurascens*)	IC_50_ = 0.63 ± 0.01 mg/mL	Pepsin (pH 2) + pancreatin (pH 7.5); 1:20 (w/w); 37°C; 2 + 2 h	[20]
Cricket (*G. assimilis*)	355.99 ± 2.61 μmol TE/g protein	Flavourzyme; 100 U/mL; pH 7; 50°C; 2 h	[19]
Cricket (*G. assimilis*)	IC_50_ = 71 μg/mL	Flavourzyme; 100 U/mL; pH 7; 50°C; 2 h	[13]
Cricket (*G. assimilis*)	0.26 mM TE/mg (after digestion)	Alcalase; 3% (w/w); pH 8; 50°C; 40 min	[27]
Cricket (*A. domesticus*)	305.9 ± 3.5 mg Trolox/g peptide	Alcalase; 5%; pH 9; 50°C; 3 h	[28]
Cricket (*A. domesticus*)	67.4 ± 1.1% inhibition at a concentration of 50 mg/mL	Alcalase; 3% (w/w); pH 8; 50°C; 90 min; ultrasonication	[29]

*Method: FRAP (Ferric Reducing Antioxidant Power):*			
Locust (*L. migratoria*)	1.49 ± 0.03 μmol Trolox/100 g	Alcalase; 3% (w/w); pH 8; 50°C; 90 min; ultrasonication	[24, 25]
Cricket (*G. assimilis*)	44.68 ± 0.77 μmol TE/g protein	Flavourzyme; 100 U/mL; pH 7; 50°C; 2 h	[19]
Cricket (*G. assimilis*)	624 ± 37 μmol TE/g	Flavourzyme; 100 U/mL; pH 7; 50°C; 2 h	[13]
Cricket (*G. sigillatus*)	991 μmol TE/mg	Alcalase; 3% (w/w); pH 8; 50°C; 40 min	[27]
Cricket (*A. domesticus*)	290.4 ± 6.7 mg Trolox/g peptide	Alcalase; 5%; pH 9; 50°C; 3 h	[28]
Cricket (*A. domesticus*)	T2: 185.3 ± 1.9 μmol TE/100 g	Alcalase; 3% (w/w); pH 8; 50°C; 90 min; ultrasonication	[29]

##### 3.9.1.1. DPPH Method

This method can be used to measure antioxidant activity in both polar and nonpolar extracts because DPPH radicals are soluble in various organic solvents (e.g., methanol, ethanol). The DPPH method is used to evaluate the antioxidant capacity of peptides from insect protein hydrolysis, which works through the mechanism of hydrogen atom donation or single electron transfer. In this context, peptides containing amino acid residues with phenolic groups or sulfhydryl groups, such as tyrosine, tryptophan, histidine, or cysteine, are believed to play a role in neutralizing DPPH free radicals by donating hydrogen atoms or electrons.

Grasshopper protein hydrolyzate treated with Alcalase enzyme (3% w/w) and ultrasonicated for 90 min at pH 8 and 50°C showed the highest inhibitory activity against DPPH radicals of 82.00 ± 0.95% at a concentration of 50 mg/mL [24, 25]. This activity indicates the high antioxidant potential of the resulting peptide. Meanwhile, with the same hydrolysis conditions, the cricket protein hydrolyzate sample had lower antioxidant inhibitory activity, namely 60.7 ± 1.3% inhibition at a concentration of 50 mg/mL [29]. Another report showed that cricket protein hydrolyzate had an antioxidant activity of 393.6 ± 3.7 mg TE/g peptide [28]. Therefore, it is important to consider that differences in DPPH concentration, sample solution, incubation time, and unit of measurement may affect the final results and make comparisons between studies only descriptive rather than quantitatively comparative.

##### 3.9.1.2. ABTS Method

The ABTS method is widely used to measure the antioxidant capacity of compounds through a free radical capture mechanism. This method can be used to measure antioxidant activity in both polar (hydrophilic) and nonpolar (lipophilic) extracts, usually in aqueous conditions. This assay involves the reaction between the blue‐green ABTS^+^ radical and antioxidant compounds capable of donating electrons or hydrogen, causing a color change that can be measured spectrophotometrically [19]. Antioxidant activity in this method is strongly influenced by the presence of certain functional groups in the structure of the bioactive compound. In peptides, the most important functional groups are free amino groups (‐NH_2_), carboxyl groups (‐COOH), thiol groups (‐SH) from amino acids such as cysteine and methionine, and aromatic rings from tyrosine, tryptophan, and phenylalanine. These groups function as electron or proton donors that can neutralize free radicals by stabilizing the radicals through resonance or hydrogen bonding [28].

Antioxidant activity through the ABTS mechanism showed a consistent pattern among various insect species. Protein hydrolyzate from *P. beltrani* showed the highest activity of 554.01 μM TE/mg protein, especially in the < 3 kDa peptide fraction obtained through ultrafiltration [28]. These results strengthen the evidence that the molecular size of peptides greatly affects their biological activity. Proteins from *L. migratoria* also showed high ABTS radical capture activity, with an inhibition value of 88.00 ± 1.20% at an extract concentration of 50 mg/mL [31]. Although these percentage inhibition values reflect potential activity, it should be noted that these results are strongly influenced by the test concentration, reaction time, and media conditions, so comparisons between studies should be made with respect to uniform test parameters.

The combination of enzymatic hydrolysis methods (e.g., with Flavourzyme or Alcalase) and pretreatment technologies such as ultrasonication or microwave is proven to enhance the release of small‐sized bioactive peptides that contribute to increased antioxidant activity. For example, proteins from *L. migratoria* hydrolyzed using the Alcalase enzyme showed an ABTS radical capture activity (at a concentration of 50 mg/mL) of 43.26% without pretreatment, which then increased to 60.21% after microwave treatment and reached 88.00% after ultrasonication treatment, showing an almost twofold increase over the untreated control [25]. This suggests that physical pretreatment is able to accelerate the degradation of protein structures, thereby facilitating the release of biologically active small peptides.

##### 3.9.1.3. FRAP Method

In the FRAP method, antioxidant activity is measured based on the compounds’ ability to reduce ferrous ions (Fe^3+^) to ferric ions (Fe^2+^) through electron donation. Compounds that act as electron donors in this reaction are generally functional groups found in aromatic and highly polarized amino acids, such as hydroxyl groups on tyrosine, indole groups on tryptophan, and thiol groups on cysteine and methionine. In addition, free amino groups (‐NH_2_) and carboxyl groups (‐COOH) at the peptide end also contribute to the ability to reduce ferric ions. Peptides with these structures are able to stabilize ferric ions through coordination bonds or charge delocalization, thus showing high reduction activity in the FRAP assay [28]. This method is commonly used to measure antioxidant activity in polar (hydrophilic) extracts because it requires an aqueous medium for the reduction of ferric ions (Fe^3+^) to ferrous ions (Fe^2+^).

In this test, the cricket *G. sigillatus* produced the highest value of 991 μmol TE/mg, indicating a remarkable reduction capacity [27]. This was followed by *G. assimilis* with FRAP values reaching 624 ± 37 μmol TE/g in a study by de Matos et al. [13], which also used the Flavourzyme enzyme singly. Interestingly, the use of ultrasonication pretreatment consistently provides a significant enhancement of antioxidant activity, including FRAP. For instance, the study by Lone et al. [31] showed a value of 185.3 ± 1.9 μmol TE/100 g after ultrasonication treatment.

#### 3.9.2. Anti‐Inflammatory Activity

Peptides resulting from protein hydrolysis from locust‐type insects (*S. gregaria* and *S. purpurascens*) show high potential anti‐inflammatory activity through the inhibition of proinflammatory enzymes such as LOX, cyclooxygenase‐2 COX, and iNOS (Table 12). The mechanism of peptide inhibition of these inflammatory enzymes generally occurs through direct interaction between the peptide and the active site of the enzyme, resulting in impaired access or catalytic activity to its natural substrate. LOX and COX‐2 enzymes play an important role in the arachidonic acid metabolic pathway [32]. LOX converts arachidonic acid into leukotrienes, while COX‐2 converts it into prostaglandins. Both are major inflammatory mediators. Meanwhile, iNOS catalyzes the formation of large amounts of nitric oxide (NO) during inflammatory conditions, which contributes to oxidative stress and tissue damage.

**TABLE 12:** Comparison of protein hydrolysis process conditions on anti‐inflammatory, antihypertensive, and antidiabetic activity.

Insect name	Analysis method	Activity value	Positive control	Hydrolysis process conditions (enzyme type and concentration E/S; pH; T; t)	References
*Anti-inflammatory:*					
Locust (*S. gregaria*)	Lipoxygenase	IC_50_ = 3.13 μg/mL	Aronia melanocarpa extract (IC_50_ = 30.3–91.0 μg/mL)	α‐Amylase, pepsin, pancreatin, and bile extract solution; pH 7; 37°C; 3–4 h	[18]
	COX‐2	IC_50_ = 5.05 μg/mL	Purple basil extract (IC_50_ = 5.0 μg/mL)		

Grasshopper (*S. purpurascens*)	COX‐2	IC_50_ = 0.52 ± 0.12 mg/mL	n/a	Pepsin (pH 2) and pancreatin (pH 7.5); 1:20 (w/w); 37°C; 2 + 2 h	[20]
	iNOS	IC_50_ = 0.51 ± 0.01 mg/mL	n/a		

*Antihypertensive:*					
Locust (*P. beltrani*)	ACE inhibitory	IC_50_ = 0.50 mg/mL	Captopril (IC_50_ = 0.00217 mg/mL)	Pepsin (pH 2) + trypsin and chymotrypsin (pH 7.8); 1:10 (w/w); 37°C; 5 h	[23]

Cricket (*G. assimilis*)	ACE inhibitory	50.84% inhibition at a concentration of 0.5 mg/mL	Captopril (IC_50_ = 0.012 mg/mL)	Flavourzyme + Alcalase (1:1); 100 U/mL total (1:20 w/v); pH 7; 50°C; 2 h	[12]
	ACE inhibitory	42.22 ± 6.29% inhibition at a concentration of 0.5 mg/mL	Captopril (IC_50_ = 0.011 mg/mL)	Flavourzyme + Alcalase; 50 + 50 U/mL; pH 7; 50°C; 2 h	[19]

Cricket (*G. bimaculatus*)	ACE inhibitory	IC_50_ = 0.047 mg/mL	Captopril (IC_50_ = 0.0082 mg/mL)	Alcalase; 72 mU/g protein; n/a; 55°C; 8 h	[16]

Cricket (*G. sigillatus*)	ACE inhibitory	> 90% inhibition at a concentration of 5 mg/mL, or IC_50_ = 0.051–0.089 mg/mL	Captopril (IC_50_ = 0.006 mg/mL)	Alcalase; 3% (w/w); pH 8; 50°C; 60–80 min	[27]

Cricket (*A. domesticus*)	ACE inhibitory	57.93% inhibition (concentration n/a)	Captopril (IC_50_ = 0.0054 mg/mL)	Alcalase; 5%; pH 9; 50°C; 3 h	[28]

*Antidiabetic:*					
Locust (*P. beltrani*)	α‐Amylase	IC_50_ = 0.49 mg/mL or 0.68 mg/mL (*F* < 3 kDa)	Acarbose (IC_50_ = 0.29 mg/mL)	Pepsin (pH 2) + trypsin and chymotrypsin (pH 7.8); 1:10 (w/w); 37°C; 5 h	[23]

Cricket (*G. assimilis*)	α‐Amylase	IC_50_ = 1.99 mg/mL, or 55.40% inhibition at a concentration of 5.0 mg/mL	Acarbose (IC_50_ = 0.31 mg/mL)	Flavourzyme + Neutrase (1:1); 100 U/mL total (1:20 w/v); pH 7; 50°C; 2 h	[12]
	α‐Amylase	47.87 ± 0.91% inhibition at a concentration of 5.0 mg/mL	Acarbose (IC_50_ = 0.31 mg/mL)	Flavourzyme + Neutrase; 50 + 50 U/mL; pH 7; 50°C; 2 h	[19]
	α‐Glucosidase	IC_50_ = 6.21 mg/mL, or 17.07% inhibition at a concentration of 2.5 mg/mL	Acarbose (IC_50_ = 0.41 mg/mL)	Flavourzyme + Neutrase (1:1); 100 U/mL total (1:20 w/v); pH 7; 50°C; 2 h	[12]
	α‐Glucosidase	12.73 ± 0.99% inhibition at a concentration of 2.5 mg/mL	Acarbose (IC_50_ = 0.41 mg/mL)	Flavourzyme + Neutrase; 50 + 50 U/mL; pH 7; 50°C; 2 h	[19]

Based on the results of Zielińska et al. [18], peptides from *S. gregaria* locust that had undergone simulated gastrointestinal digestion (using α‐amylase, pepsin, pancreatin, and bile) showed significant LOX and COX‐2 inhibitory activity, with IC_50_ values of 3.13 and 5.05 μg/mL, respectively. Meanwhile, a study by Villaseñor et al. [20] found that peptides from the hydrolysis of *S. purpurascens* protein using pepsin and pancreatin enzymes have inhibitory activity against COX‐2 (IC_50_ = 0.52 ± 0.12 mg/mL) and iNOS (IC_50_ = 0.51 ± 0.01 mg/mL). Through molecular docking assays, some peptide sequences showed strong binding affinity toward the active site of COX‐2 and iNOS enzymes, indicating a possible competitive mechanism toward natural substrates, namely arachidonic acid (for COX‐2 and LOX) and L‐arginine (for iNOS). However, most of these data are still limited to in vitro and in silico assays, and to date no advanced assays using cell culture or animal models (in vivo) have been reported.

#### 3.9.3. Antihypertensive Activity

An ACE inhibitor is a key enzyme in the renin–angiotensin system, which plays a role in the conversion of angiotensin I to angiotensin II, a vasoconstrictor peptide that causes an increase in blood pressure [33]. Therefore, inhibition of ACE activity is one of the main strategies in hypertension control. Peptides with antihypertensive activity generally have certain characteristics, including small molecular size, usually consisting of 2–12 amino acids, which facilitates penetration into biological targets. These peptides tend to contain hydrophobic amino acid residues such as proline, phenylalanine, or tyrosine, particularly in the C‐terminal position, which play an important role in binding to the ACE active site. The presence of positively charged amino acids such as lysine or arginine also increases the affinity toward the enzyme. In addition, effective antihypertensive peptides are generally stable against digestion, so they remain active after passing through the gastrointestinal tract and can perform their functions in the body optimally [27].

Research by Montiel‐Aguilar et al. [23] showed that peptides from *P. beltrani* produced through hydrolysis using pepsin, trypsin, and chymotrypsin enzymes have ACE inhibitory activity with an IC_50_ value of 0.50 mg/mL. Higher activity was obtained from peptides hydrolyzed from *G. sigillatus* using the Alcalase enzyme, with IC_50_ values ranging from 0.051–0.089 mg/mL and ACE inhibition effectiveness of more than 90% at a concentration of 1 mg/mL [27]. Meanwhile, peptides from *G. bimaculatus* also showed strong inhibitory activity with an IC_50_ value of 0.047 mg/mL [16].

Protein hydrolyzate from *G. assimilis* processed using a combination of Flavourzyme and Alcalase enzymes produced ACE inhibitory activity of 50.84% at a concentration of 1 mg/mL, and 42.22% before simulated digestion [12, 19]. Meanwhile, peptides from *A. domesticus* were able to inhibit ACE activity by 57.93% at a concentration of 1 mg/mL [28]. These findings corroborate that insect proteins, particularly Orthoptera, are a potential source of bioactive peptides with promising antihypertensive activity.

#### 3.9.4. Antidiabetic Activity

The ability of insect peptides to inhibit carbohydrate‐degrading enzymes such as α‐amylase and α‐glucosidase makes them strong candidates in the management of type 2 diabetes [34]. Both enzymes play an important role in the process of carbohydrate digestion: α‐Amylase catalyzes the breakdown of starch into oligosaccharides, while α‐glucosidase breaks down oligosaccharides into glucose, which is then absorbed into the bloodstream. Some peptides are known to contain hydrophobic, charged, or aromatic amino acids, which are able to interact with enzymes through hydrogen, electrostatic, or hydrophobic bonds. The inhibitory mechanism of these peptides is thought to involve a direct interaction between certain amino acid residues in the peptide (such as leucine, tyrosine, or lysine) with the active site of the enzyme via hydrogen, electrostatic, or hydrophobic bonds, thereby interfering with substrate binding and catalytic activity of the enzyme [35]. This activity can take place competitively or noncompetitively depending on the structure and amino acid sequence in the bioactive peptide. Inhibition of these two enzymes may slow the release of glucose from food and help control blood glucose spikes after eating [36].

Peptides from *P. beltrani* showed an IC_50_ value of 0.49 mg/mL against α‐amylase activity, indicating strong inhibitory potential [23]. This decrease in enzyme activity was also observed in the peptide of *G. assimilis* hydrolyzed by the combination of Flavourzyme and Neutrase. At a concentration of 5 mg/mL, the peptide fraction showed α‐amylase inhibition of 55.40% (IC_50_ = 1.99 mg/mL) [12] and 47.87% [19]. Activity against α‐glucosidase was also reported at the same concentration, with inhibition values of 17.07% (IC_50_ = 6.21 mg/mL) [12] and 12.73% [19], which still contributed to the overall antidiabetic effect.

## 4. Conclusion

One key contribution of this review is the identification of convergent hydrolysis conditions across multiple studies, particularly the effectiveness of Alcalase at approximately 3% (w/w), pH 8–9, 50°C, and hydrolysis times around 90 min, which consistently resulted in high degrees of hydrolysis and enhanced biofunctional activity. Furthermore, it demonstrates that single enzyme systems are more effective for targeted peptide production. Despite the promising biofunctional results, allergenicity reduction remains underexplored, with only few studies evaluating IgE reactivity after hydrolysis; thus, further research is needed.

## Author Contributions

Slamet Hadi Kusumah: conceptualization, writing–original draft, methodology, software, data curation, project administration, and formal analysis. Nurheni Sri Palupi: conceptualization, writing–review and editing, supervision, and validation. Azis Boing Sitanggang and Fitriya Nur Annisa Dewi: supervision and writing–review and editing. Saraswati: conceptualization, validation, methodology, writing–review and editing, formal analysis, and supervision.

## Funding

The publication costs for this article were funded by the Indonesian Endowment Fund for Education (Lembaga Pengelola Dana Pendidikan) (Grant Number: 00273/J5.2.3./BPI.06/9/2022) through the international journal publication assistance fund scheme in the Indonesian Education Scholarship Program (BPI PPAPT Kemdiktisaintek).

## Conflicts of Interest

The authors declare no conflicts of interest.

## Supporting Information

Additional supporting information can be found online in the Supporting Information section.

## Supporting information


**Supporting Information 1** PRISMA checklist.

**Supporting Information 2** Distribution of article search results is based on keywords and study sources.

**Supporting Information 3** Article selection results are based on title and abstract.

**Supporting Information 4** Screening results are based on full text review.

## Data Availability

The data that support the findings of this study are available from the corresponding author upon reasonable request.
