# Supplementary material for: Enzymatic Hydrolysis Methods of Insect Orthoptera Protein: A Systematic Review
Source: Int J Food Sci. 2026 Apr 24;2026:9091997. doi: 10.1155/ijfo/9091997 (PMC13108587; doi:10.1155/ijfo/9091997)
Supplement: Supplementary file 2 — Supporting Information 2 Distribution of article search results is based on keywords and study sources. [file IJFO-2026-9091997-s002.docx]

**Supplementary Material 2**

**Distribution of article search results based on keywords and study sources**

| No. | Keyword | Study source | Search date  (DD/MM/YY) | Search results | | | | | | |
| --- | --- | --- | --- | --- | --- | --- | --- | --- | --- | --- |
|  |  |  |  | Research article | Review article | Encyclopedia | Book chapters | Book review | Other | Sum |
| 1 | protein hydrolysis AND edible insects | ScienceDirect | 05/02/2025 | 62 | 24 | 4 | 17 | 1 | 8 | 116 |
|  |  | Scopus | 10/02/2025 | 15 | 1 | 0 | 0 | 0 | 0 | 16 |
|  |  | PubMed | 11/02/2025 | 13 | 1 | 0 | 0 | 0 | 0 | 14 |
|  |  | Wiley | 08/02/2025 | 17 | 1 | 0 | 0 | 0 | 2 | 20 |
|  |  | **Sum** |  | **107** | **27** | **4** | **17** | **1** | **10** | **166** |
| 2 | protein hydrolysis AND (orthoptera OR grasshopper OR locust OR cricket) | ScienceDirect | 06/02/2025 | 25 | 10 | 5 | 15 | 0 | 3 | 58 |
|  |  | Scopus | 10/02/2025 | 11 | 0 | 0 | 0 | 0 | 1 | 12 |
|  |  | PubMed | 11/02/2025 | 0 | 0 | 0 | 0 | 0 | 0 | 0 |
|  |  | Wiley | 08/02/2025 | 1 | 0 | 0 | 0 | 0 | 0 | 1 |
|  |  | **Sum** |  | **37** | **10** | **5** | **15** | **0** | **4** | **71** |
| 3 | protein hydrolysis AND edible insects AND protein content | ScienceDirect | 06/02/2025 | 56 | 18 | 2 | 15 | 0 | 6 | 97 |
|  |  | Scopus | 10/02/2025 | 2 | 1 | 0 | 0 | 0 | 0 | 3 |
|  |  | PubMed | 11/02/2025 | 0 | 1 | 0 | 0 | 0 | 0 | 1 |
|  |  | Wiley | 08/02/2025 | 15 | 0 | 0 | 0 | 0 | 2 | 17 |
|  |  | **Sum** |  | **73** | **20** | **2** | **15** | **0** | **8** | **118** |
| 4 | protein hydrolysis AND edible insects AND orthoptera AND protein content | ScienceDirect | 06/02/2025 | 9 | 5 | 0 | 6 | 0 | 0 | 20 |
|  |  | Scopus | 10/02/2025 | 1 | 0 | 0 | 0 | 0 | 0 | 1 |
|  |  | PubMed | 11/02/2025 | 0 | 0 | 0 | 0 | 0 | 0 | 0 |
|  |  | Wiley | 08/02/2025 | 2 | 1 | 0 | 0 | 0 | 0 | 3 |
|  |  | **Sum** |  | **12** | **6** | **0** | **6** | **0** | **0** | **24** |
| 5 | protein hydrolysis AND edible insects AND degree of protein hydrolysis | ScienceDirect | 06/02/2025 | 6 | 3 | 0 | 0 | 0 | 0 | 9 |
|  |  | Scopus | 10/02/2025 | 1 | 0 | 0 | 0 | 0 | 0 | 1 |
|  |  | PubMed | 11/02/2025 | 4 | 1 | 0 | 0 | 0 | 0 | 5 |
|  |  | Wiley | 08/02/2025 | 2 | 0 | 0 | 0 | 0 | 0 | 2 |
|  |  | **Sum** |  | **13** | **4** | **0** | **0** | **0** | **0** | **17** |
| 6 | protein hydrolysis AND edible insects AND orthoptera AND degree of protein hydrolysis | ScienceDirect | 06/02/2025 | 1 | 0 | 0 | 0 | 0 | 0 | 1 |
|  |  | Scopus | 10/02/2025 | 0 | 0 | 0 | 0 | 0 | 0 | 0 |
|  |  | PubMed | 11/02/2025 | 0 | 0 | 0 | 0 | 0 | 0 | 0 |
|  |  | Wiley | 08/02/2025 | 0 | 0 | 0 | 0 | 0 | 0 | 0 |
|  |  | **Sum** |  | **1** | **0** | **0** | **0** | **0** | **0** | **1** |
| 7 | protein hydrolysis AND edible insects AND allergen | ScienceDirect | 08/02/2025 | 9 | 11 | 4 | 6 | 1 | 4 | 35 |
|  |  | Scopus | 10/02/2025 | 1 | 0 | 0 | 0 | 0 | 0 | 1 |
|  |  | PubMed | 11/02/2025 | 1 | 0 | 0 | 0 | 0 | 0 | 1 |
|  |  | Wiley | 08/02/2025 | 1 | 2 | 0 | 0 | 0 | 1 | 4 |
|  |  | **Sum** |  | **12** | **13** | **4** | **6** | **1** | **5** | **41** |
| 8 | protein hydrolysis AND edible insects AND orthoptera AND allergen | ScienceDirect | 08/02/2025 | 0 | 6 | 0 | 3 | 0 | 0 | 9 |
|  |  | Scopus | 10/02/2025 | 0 | 0 | 0 | 0 | 0 | 0 | 0 |
|  |  | PubMed | 11/02/2025 | 0 | 0 | 0 | 0 | 0 | 0 | 0 |
|  |  | Wiley | 08/02/2025 | 0 | 1 | 0 | 0 | 0 | 0 | 1 |
|  |  | **Sum** |  | **0** | **7** | **0** | **3** | **0** | **0** | **10** |
| 9 | protein hydrolysis AND edible insects AND allergenicity | ScienceDirect | 08/02/2025 | 14 | 16 | 2 | 9 | 0 | 6 | 47 |
|  |  | Scopus | 10/02/2025 | 1 | 0 | 0 | 0 | 0 | 0 | 1 |
|  |  | PubMed | 11/02/2025 | 0 | 0 | 0 | 0 | 0 | 0 | 0 |
|  |  | Wiley | 08/02/2025 | 4 | 2 | 0 | 0 | 0 | 2 | 8 |
|  |  | **Sum** |  | **19** | **18** | **2** | **9** | **0** | **8** | **56** |
| 10 | protein hydrolysis AND edible insects AND orthoptera AND allergenicity | ScienceDirect | 08/02/2025 | 1 | 6 | 0 | 4 | 0 | 0 | 11 |
|  |  | Scopus | 10/02/2025 | 0 | 0 | 0 | 0 | 0 | 0 | 0 |
|  |  | PubMed | 11/02/2025 | 0 | 0 | 0 | 0 | 0 | 0 | 0 |
|  |  | Wiley | 08/02/2025 | 0 | 1 | 0 | 0 | 0 | 0 | 1 |
|  |  | **Sum** |  | **1** | **7** | **0** | **4** | **0** | **0** | **12** |
| 11 | protein hydrolysis AND edible insects AND bioactive peptides | ScienceDirect | 08/02/2025 | 28 | 14 | 3 | 12 | 0 | 7 | 64 |
|  |  | Scopus | 10/02/2025 | 0 | 0 | 0 | 0 | 0 | 0 | 0 |
|  |  | PubMed | 11/02/2025 | 0 | 0 | 0 | 0 | 0 | 0 | 0 |
|  |  | Wiley | 08/02/2025 | 10 | 2 | 0 | 0 | 0 | 2 | 14 |
|  |  | **Sum** |  | **38** | **16** | **3** | **12** | **0** | **9** | **78** |
| 12 | protein hydrolysis AND edible insects AND orthoptera AND bioactive peptides | ScienceDirect | 08/02/2025 | 6 | 3 | 0 | 4 | 0 | 0 | 13 |
|  |  | Scopus | 10/02/2025 | 0 | 0 | 0 | 0 | 0 | 0 | 0 |
|  |  | PubMed | 11/02/2025 | 0 | 0 | 0 | 0 | 0 | 0 | 0 |
|  |  | Wiley | 08/02/2025 | 1 | 1 | 0 | 0 | 0 | 0 | 2 |
|  |  | **Sum** |  | **7** | **4** | **0** | **4** | **0** | **0** | **15** |
| 13 | protein hydrolysis AND edible insects AND biofunctionality | ScienceDirect | 08/02/2025 | 1 | 4 | 0 | 0 | 0 | 0 | 5 |
|  |  | Scopus | 10/02/2025 | 0 | 0 | 0 | 0 | 0 | 0 | 0 |
|  |  | PubMed | 11/02/2025 | 0 | 0 | 0 | 0 | 0 | 0 | 0 |
|  |  | Wiley | 08/02/2025 | 0 | 0 | 0 | 0 | 0 | 0 | 0 |
|  |  | **Sum** |  | **1** | **4** | **0** | **0** | **0** | **0** | **5** |
| 14 | protein hydrolysis AND edible insects AND orthoptera AND biofunctionality | ScienceDirect | 08/02/2025 | 1 | 2 | 0 | 0 | 0 | 0 | 3 |
|  |  | Scopus | 10/02/2025 | 0 | 0 | 0 | 0 | 0 | 0 | 0 |
|  |  | PubMed | 11/02/2025 | 0 | 0 | 0 | 0 | 0 | 0 | 0 |
|  |  | Wiley | 08/02/2025 | 0 | 0 | 0 | 0 | 0 | 0 | 0 |
|  |  | **Sum** |  | **1** | **2** | **0** | **0** | **0** | **0** | **3** |
| 15 | protein hydrolysis AND edible insects AND (anti-inflammatory OR antioxidants) | ScienceDirect | 08/02/2025 | 44 | 21 | 3 | 0 | 11 | 8 | 87 |
|  |  | Scopus | 10/02/2025 | 6 | 0 | 0 | 0 | 0 | 0 | 6 |
|  |  | PubMed | 11/02/2025 | 5 | 0 | 0 | 0 | 0 | 0 | 5 |
|  |  | Wiley | 08/02/2025 | 12 | 3 | 0 | 0 | 0 | 2 | 17 |
|  |  | **Sum** |  | **67** | **24** | **3** | **0** | **11** | **10** | **115** |
| 16 | protein hydrolysis AND edible insects AND orthoptera AND (anti-inflammatory OR antioxidants) | ScienceDirect | 08/02/2025 | 7 | 5 | 0 | 4 | 0 | 0 | 16 |
|  |  | Scopus | 10/02/2025 | 1 | 0 | 0 | 0 | 0 | 0 | 1 |
|  |  | PubMed | 11/02/2025 | 0 | 0 | 0 | 0 | 0 | 0 | 0 |
|  |  | Wiley | 08/02/2025 | 2 | 1 | 0 | 0 | 0 | 0 | 3 |
|  |  | **Sum** |  | **10** | **6** | **0** | **4** | **0** | **0** | **20** |

Note: Other (discussion, mini reviews, conference abstracts, correspondence, news)

**Recapitulation:**

| Studi source | Research article | Review article | Encyclopedia | Book chapters | Book review | Other | Sum |
| --- | --- | --- | --- | --- | --- | --- | --- |
| Elsevier’s ScienceDirect | 270 | 148 | 23 | 95 | 13 | 42 | 591 |
| Scopus | 39 | 2 | 0 | 0 | 0 | 1 | 42 |
| PubMed | 23 | 3 | 0 | 0 | 0 | 0 | 26 |
| Wiley online library | 67 | 15 | 0 | 0 | 0 | 11 | 93 |
| **Total** | **399** | **168** | **23** | **95** | **13** | **54** | **752** |
| Description | Study learned, proceed to next stage | Article not studied, References removed | Article not studied, References removed | Article not studied, References removed | Article not studied, References removed | Article not studied, References removed |  |
