# Supplementary material for: Enzymatic Hydrolysis Methods of Insect Orthoptera Protein: A Systematic Review
Source: Int J Food Sci. 2026 Apr 24;2026:9091997. doi: 10.1155/ijfo/9091997 (PMC13108587; doi:10.1155/ijfo/9091997)
Supplement: Supplementary file 3 — Supporting Information 3 Article selection results are based on title and abstract. [file IJFO-2026-9091997-s003.pdf]

Supplementary Material 3
Article selection results based on title and abstract

No.	Authors	Title	Year	Source	DOI	Abstract	Relevant or Irrelevant	Reason	Decision
1	Simona Rimoldi, Chiara Ceccotti, Fabio Brambilla, Filippo Faccenda, Micaela Antonini, Genciana Terova	Potential of shrimp waste meal and insect exuviae as sustainable sources of chitin for fish feeds	2023	Aquaculture	https://doi.org/10.1016/j.aquaculture.2023.739256	Aquaculture is one of the world's fastest growing food-producing sectors, providing more than half of all fish consumed globally for human nutrition. However, to maintain such growth and meet the increasing demand for aquatic food, sustainable raw materials for fish feeds are needed. In this regard, insects represent one of the most promising alternatives to fish meal (FM) protein source for use in aquafeeds. In addition to protein, insects contain bioactive compounds, such as chitin, which is a natural polysaccharide abundantly present in the pupal exuviae of some insects. Studies have shown that dietary chitin or its derivate chitosan acts as a prebiotic thus modulating the gut microbial communities of fish. Accordingly, the present study aimed to evaluate the effect of two waste products rich in chitin, i.e., shrimp head meal (SHM), and insect (Hermetia illucens) pupal exuviae on the gut microbiota of rainbow trout (Oncorhynchus mykiss). Three isoproteic, isolipidic, and isoenergetic diets containing either FM, SHD, or a combination of FM and 1.6% of pupal exuviae meal (PEM) were tested through a 91-day feeding trial. At the end of the experiment, no differences in final mean body weight, specific growth rate, and feed conversion ratio values were observed between fish experimental groups. Mortality was <1% and it did not correlate with diet for the entire duration of the trial. However, a modulatory effect of dietary pupal exuviae on fish gut microbiota was detected. Indeed, gut bacterial species richness improved by including insect exuviae. In particular, Firmicutes and Actinobacteria phyla, mainly represented by Bacillus, Facklamia, Brevibacterium, and Corynebacterium genera, were enriched in trout receiving pupal exuviae. These genera are chitinolytic and short-chain fatty acids (SCFAs)-producing bacteria. SCFAs production was confirmed by gas chromatography analysis, which detected the highest amount of butyrate in feces of trout fed with pupal exuviae meal. Functional inference analysis of intestinal microbiota using PICRUST metagenome prediction tool, showed differences in response to diet. In particular, eleven pathways were significantly different between control fish (FM) and fish fed the PEM diet, whereas twenty functional traits were significantly different between the FM and SHM fish groups. Overall, our data confirmed that chitin from insect's pupal exuviae represents a promising functional ingredient, better than SHM, for positively modulating gut microbiota communities of rainbow trout.	Irrelevant	Title and abstract are not relevant with the topic	References removed
2	M Maciejewska, A DÄ…browska, M Cano-Lamadrid	Sustainable Protein Sources: Functional Analysis of Tenebrio molitor Hydrolysates and Attitudes of Consumers in Poland and Spain Toward Insect-Based Foods	2025	Foods	10.3390/foods14020333	This study explores the potential of Tenebrio molitor protein hydrolysates as functional food ingredients, evaluating their bioactivity and consumer acceptance of the incorporation of edible insects into food across Poland and Spain. By aligning technical advancements with consumer preferences, this research bridges the gap between laboratory innovation and market feasibility, contributing to the development of sustainable functional foods. The study optimized the process of enzyme hydrolysis using serine protease from Cucurbita ficifolia, thereby enhancing DPPH scavenging capacity increased from 3.15 Â± 0.53 to 8.17 Â± 0.62 ÂµM Trolox/mL and ABTS decolorization capacity increased from 4.29 Â± 0.01 to 10.29 Â± 0.01 ÂµM Trolox/mL after 5 h of hydrolysis. Consumer surveys incorporating the Food Neophobia, Insect Phobia, and Entomophagy Scales revealed demographic and cultural influences on entomophagy acceptance. Among respondents, 27.1% in Poland and 25.7% in Spain had previously consumed insect-based products, while Polish participants showed a higher willingness to adopt insect-enriched foods. The study confirmed that hydrolysis enhances the antioxidant activity of T.Â molitor protein hydrolysates and that demographic and cultural factors significantly influence consumer acceptance of insect-based foods. Â© 2025 by the authors.	Relevant	Title and abstract are relevant to the topic	References are used for the full review stage

3	Suttida Chukiatsiri, Nattakarn Wongsrangsap, Pichamon Kiatwuthinon, Wannarat Phonphoem	Purification and identification of novel antioxidant peptides derived from Bombyx mori pupae hydrolysates	2024	Biochemistry and Biophysics Reports	https://doi.org/10.1016/j.bbrep.2024.101707	The biological importance of antioxidant peptides was the focus of new natural sources of food preservatives. Bombyx mori pupae are considered a valuable by-product of the silk-reeling industry due to their high-quality protein content. This study aimed to purify and identify the antioxidant peptides obtained from enzymatically hydrolyzed B. mori pupae, which could be used as new sources of natural food preservatives. Among the prepared hydrolysates, pepsin hydrolysate with the highest antioxidant activities was purified sequentially using ultrafiltration and reversed-phase high-performance liquid chromatography (RP-HPLC). The DPPH radical scavenging and ferrous ion chelating activity were used to evaluate antioxidant activity. Fractions with high activity were further analyzed by liquid chromatography-tandem mass spectrometry (LCâ€"MS/MS). Three peptides were identified as Glu-Asn-Ile-Ile-Leu-Phe-Arg (ENIILFR), Leu-Asn-Lys-Asp-Leu-Met-Arg (LNKDLMR), and Met-Leu-Ile-Ile-Ile-Met-Arg (MLIIIMR), respectively. All three novel identified peptides exhibited significantly stronger antioxidant capacity than synthetic antioxidants used in the food industry, including butylated hydroxyanisole (BHA), and butylated hydroxytoluene (BHT). ENIILFR showed the best antioxidant activity. These findings indicate that the three peptides have potential applications as natural antioxidants in the food industry.	Relevant	Title and abstract are relevant to the topic	References are used for the full review stage
4	Aymar Rodrigue Fogang Mba, Germain Kansci, MichÃ¨le Viau, Lucie Ribourg, John Fogoh Muafor, Nordine Hafnaoui, Philippe Le Gall, Claude Genot	Growing conditions and morphotypes of African palm weevil (Rhynchophorus phoenicis) larvae influence their lipophilic nutrient but not their amino acid compositions	2018	Journal of Food Composition and Analysis	https://doi.org/10.1016/j.jfca.2018.02.012	African palm weevil (Rhynchophorus phoenicis, Rp) larvae are a potential source of lipids and proteins of good quality for human consumption. To evaluate the influence of growing conditions on nutrient composition of larvae grown on different raffia palms, larvae (3 larvae batches per condition) were collected from the wild (yellow wild or white wild larvae) or from a breeding system (white larvae). Growing conditions influenced the morphometric parameters of the larvae and their lipid content and composition. As highlighted by principal component analysis, wild larvae exhibited greater morphometric parameters (weight, length) and higher energy, lipid, carotenoid and total fatty acid contents than larvae from breeding system. As compared to white larvae, yellow larvae contained more energy and more lipids (27.7â€¯Â±â€¯2.3â€¯g/100â€¯g fresh weight vs around 19.8â€¯g/100â€¯g), less polyunsaturated fatty acids (0.5â€¯Â±â€¯0.1â€¯g/100â€¯g vs 0.8â€¯g/100â€¯g) and tocopherols (2.25â€¯Â±â€¯0.23â€¯mg/100â€¯g vs around 4.5â€¯mg/100â€¯g), and, explaining their color, more carotenoids (800â€¯Î¼g/100â€¯g vs 280â€"390â€¯Î¼g/100â€¯g). Rp larvae contained from 7.8â€¯Â±â€¯0.3 (white wild) to 8.8â€¯Â±â€¯0.9 (yellow wild) g/100â€¯g proteins and high levels of indispensable amino acids, with no effect of growing conditions. Their nitrogen to protein conversion factors varied from 6.08â€¯Â±â€¯0.3 (white wild) to 7.42â€¯Â±â€¯0.4 (yellow wild), This work gives a new and reliable insight on nutrient content variations in palm weevil larvae.	Irrelevant	Title and abstract are not relevant with the topic	References removed
5	Hale Ä°nci Ã–ztÃ¼rk, Aysun OraÃ§	Harvesting bioactive peptides from sustainable protein sources: Unveiling technological and functional properties through in silico analyses	2024	Food and Humanity	https://doi.org/10.1016/j.foohum.2024.100294	Bioactive peptides, usually characterized by short amino acid sequences, are functional components obtained from precursor proteins by enzymatic, chemical, or other processing conditions. These peptides are known to have a wide range of effects on human health. In particular, protein-rich foods have been identified as natural sources of bioactive peptides. Furthermore, industrial by-products or wastes from various food processes have the potential to be a source of bioactive peptides. In this context, it is important to isolate proteins from food wastes, hydrolyze proteins to obtain peptides, determine the resulting peptide sequences, and characterize both functional and technological properties. Additionally, computational in silico analyses offer innovative and practical approaches for determining the properties of the peptides, offering significant time savings. This paper aims to present information on the extraction of bioactive peptides from sustainable protein sources, the identification of peptide sequences, and the determination of functional and technological properties of peptides by current in silico analyses.	Irrelevant	Title and abstract are not relevant with the topic	References removed

6	L J H Sweers, M Mishyna, R M Boom, V Fogliano, J K Keppler, C M M Lakemond	Microfiltration for effective microbiological decontamination of edible insects â€“ Protein hydrolysis, aggregation and pH are critical for protein recovery	2023	Food and Bioproducts Processing	https://doi.org/10.1016/j.fbp.2023.08.002	During the processing of insects for food and feed applications, thermal treatments are often used for decontamination purposes. However, these treatments denature proteins comprising their functional properties. Milder methods for microbiological decontamination can be used, such as microfiltration. Therefore, in this study dead-end microfiltration (0.2Â Âµm polyethersulphone membrane) was tested for decontamination of soluble fractions of lesser mealworms and house crickets obtained at pH values of 3 and 8. The results showed that dead-end microfiltration was successful in the removal of microorganisms (total viable count), removing the need for thermal treatments for the permeates. Protein recovery in the permeate was relatively low (14â€“43 %). Lesser mealworms at pH 3 gave the highest protein recovery in the permeate (43 %), as explained by the activity of endogenous proteases producing soluble peptides during microfiltration. Confocal imaging showed that the efficiency of microfiltration was reduced by membrane fouling caused by protein aggregation. The presence of lipids in the pre-filtrate does not hamper the permeate protein recovery. In conclusion, dead-end microfiltration with a 0.2Â Âµm membrane is successful in achieving microbial stability. A pH of 3 is recommended during the extraction process to obtain a higher protein recovery.	Relevant	Title and abstract are relevant to the topic	References are used for the full review stage
7	Audry Peredo-Lovillo, AdriÃ¡n HernÃ¡ndez-Mendoza, Belinda Vallejo-Cordoba, Haydee Eliza Romero-Luna	Conventional and in silico approaches to select promising food-derived bioactive peptides: A review	2022	Food Chemistry: X	https://doi.org/10.1016/j.fochx.2021.100183	The interest for food-derived bioactive peptides, either from common or unconventional sources, has increased due to their potential therapeutic effect against a wide range of diseases. The study of such bioactive peptides using conventional methods is a long journey, expensive and time-consuming. Hence, bioinformatic approaches, which can not only help to predict the formation of bioactive peptides from any known protein source, but also to analyze the protein structure/function relationship, have gained a new meaning in this scientific field. Therefore, this review aims to provides an overview of conventional characterization methods and the most recent advances in the field of in silico approaches for predicting and screening promising food-derived bioactive peptides.	Irrelevant	Title and abstract are not relevant with the topic	References removed
8	Francielle Miranda de Matos, JosÃ© Thalles Jocelino Gomes de Lacerda, Giovanna Zanetti, Ruann Janser Soares de Castro	Production of black cricket protein hydrolysates with Î±-amylase, Î±-glucosidase and angiotensin I-converting enzyme inhibitory activities using a mixture of proteases	2022	Biocatalysis and Agricultural Biotechnology	https://doi.org/10.1016/j.bcab.2022.102276	The objective of this work was to evaluate the in vitro antidiabetic and antihypertensive properties of protein hydrolysates obtained by enzymatic hydrolysis of black cricket (Gryllus assimilis) protein concentrate. The enzymatic hydrolysis was carried out by isolated application and in binary/ternary combinations of the commercial enzymes Flavourzymeâ„¢ 500Â L, Alcalaseâ„¢ 2.4Â L and Neutraseâ„¢ 0.8Â L, using an experimental mixture design. The in vitro antidiabetic properties of the protein hydrolysates were assessed through the ability to inhibit Î±-amylase and Î±-glucosidase activities, while the inhibition of the angiotensin-converting enzyme (ACE) was used to measure the potential antihypertensive property. The results showed that most of the samples had their inhibitory activities increased after enzymatic hydrolysis when compared with the non-hydrolyzed sample. The highest percentual of inhibition of Î±-amylase and Î±-glucosidase activities was 55.40% and 17.07%, respectively, detected for the protein hydrolysate produced with the binary mixture of Flavourzymeâ„¢ 500Â L and Neutraseâ„¢ 0.8Â L. The highest ACE inhibitory activity was 50.84%, detected for the sample produced by the use of a binary combination of Flavourzymeâ„¢ 500Â L and Alcalaseâ„¢ 2.4Â L. Fractionation by ultrafiltration confirmed that small peptides resulted in improved bioactive properties. Antidiabetic and antihypertensive peptides such as AGDDAPR and YPLDL were identified, in addition to sequences homologous to those already described as bioactive in the BIOPEP database. The enzymatic hydrolysis proved to be a process capable of significantly increasing the in vitro antidiabetic and antihypertensive properties of black cricket protein.	Relevant	Title and abstract are relevant to the topic	References are used for the full review stage

9	Zipporah Wangari Maimba, John N Kinyuru, George W Wanjala, James P Egonyu	Inhibiting post-harvest perishability of edible beetle grubs (Oryctes spp) by blanching and pre-treatment with sodium metabisulphite and ascorbic acid	2024	LWT	https://doi.org/10.1016/j.lwt.2023.115659	Although edible beetle grubs (Oryctes spp) are highly nutritious, post-harvest perishability limits their utilization in food processing. This study investigated the effects of blanching singly or in combination with either ascorbic acid or sodium metabisulphite or a blend of the two chemicals, on colour, microbial quality, protein quality, total phenol content and amino acid composition of the grubs after harvest. All the pretreatments effectively preserved the colour of the grubs, reduced protein oxidation and inhibited most harmful microbes to food safety limits. The treatments had insignificant effect on amino acid profiles of the larvae. Combining blanching with ascorbic acid eliminated Staphylococcus aureus from the larvae and outperformed the other treatments in enhancing the content of total phenols. The findings provide prospects for preservation of the edible beetle grubs using the affordable, readily available, and easy-to-apply heat and chemical pretreatments prior to processing into other palatable food products.	Irrelevant	Title and abstract are not relevant with the topic	References removed
10	Emmanuel Nunes, Kilian Odenthal, Nuno Nunes, TomÃ¡sia Fernandes, Igor A Fernandes, Miguel A A Pinheiro de Carvalho	Protein extracts from microalgae and cyanobacteria biomass. Techno-functional properties and bioactivity: A review	2024	Algal Research	https://doi.org/10.1016/j.algal.2024.103638	Microalgae and cyanobacteria are photosynthetic and unicellular organisms that contain considerable amounts of proteins, lipids, carbohydrates, and polyunsaturated fatty acids, among others, with applications in the cosmetic, pharmaceutical, and food industries. These microorganisms can accumulate protein up to 70Â % of total biomass depending on the microalgal strain, hence they have been regarded as an alternative protein source for the future. Microalgal proteins have important applications such as emulsifying, foaming, and gelation properties, which are important for the determination of quality and texture of foods. Some microalgal peptides possess important bioactivity with many health-benefit effects. Therefore, to maximize the production of proteins from microalgae and cyanobacteria, many protein extraction procedures have been studied to increase the economic return. They have been tested towards higher protein yields at low energy cost, the preservation of protein native properties, and lower cell debris. This later is fundamental to facilitate the subsequent purification processes so that the overall cost can be reduced. The aim of this work is to review some cell disruption processes for the extraction of protein from microalgae and cyanobacteria, considering that this step is crucial for the overall process due to the high rigidness of microalgal cell covering, which can hamper the release of proteins. It also aims at reviewing the purification techniques after cellular disruption, from conventional to more recent approaches, and finally addresses the antioxidant, antidiabetic, antihypertensive, antibacterial and other bioactive properties of microalgal protein hydrolysates and peptides.	Irrelevant	Title and abstract are not relevant with the topic	References removed
11	Denisse MarÃ-a Rivas-Navia, Alex Alberto DueÃ±as-Rivadeneira, Juan Pablo DueÃ±as-Rivadeneira, Sesan Abiodun Aransiola, Naga Raju Maddela, Ram Prasad	Bioactive compounds of insects for food use: Potentialities and risks	2023	Journal of Agriculture and Food Research	https://doi.org/10.1016/j.jafr.2023.100807	Insects are an alternative source of human and animal food that not only have nutritional characteristics and important bioactive compounds, but also can help reduce the impact of chronic non-communicable diseases; thus, derivatives from insects are a great contribution to the food and pharmaceutical industry. The aim of the present work was to highlight the chemical composition, main bioactive compounds, and anti-nutritional factors present in some insects and their applications as raw materials in the food and pharmaceutical industry through the analysis of different studies. Results indicated that entomophagy was included in the diet of many regions in the world and that it is going to become a significant component in the food industry. It was also found that bioactive compounds with potential functional properties within the chemical composition of insects such as an appropriate digestibility between 54Â % and 86Â %, peptides between 35Â % and 77Â % and other nutrients such as proteins and carbohydrates that can be a great contribution to nutrition and reduce the risk of chronic non-communicable diseases. Global agencies are of the opinion that edible insects are the viable option to over the food scarcity. However, use of food matrices from insects raises safety issues, such as anti-nutritional factors allergenicity and capacity to harbor toxins such as oxalates, tannins, alkaloids, phytates and saponins or pathogens and heavy metal. Insights presented in this work are useful to insect business (such as farmers, producers of edible insect products and consultants), researchers, and policy makers. Such insights can be an initiative for insect food companies in many ways, such as to forge collaborations with different bodies (researchers, peoples, government), improve perception of insect consumption, promote market acceptance, identify new and native edible insect species, make ecologically sustainable insect food companies, produce nutritious and delicious insect-based foods, etc.	Irrelevant	Title and abstract are not relevant with the topic	References removed

12	C Wang, J Zhang, Y Wang, C Niu, R Ma, Y Wang, Y Bai, H Luo, B Yao	Biochemical characterization of an acidophilic Î²-mannanase from Gloeophyllum trabeum CBS900.73 with significant transglycosylation activity and feed digesting ability	2016	Food Chemistry	10.1016/j.foodchem.2015.10.115	Acidophilic β-mannanases have been attracting much attention due to their excellent activity under extreme acidic conditions and significant industrial applications. In this study, a β-mannanase gene of glycoside hydrolase family 5, man5A, was cloned from Gloeophyllum trabeum CBS900.73, and successfully expressed in Pichia pastoris. Purified recombinant Man5A was acidophilic with a pH optimum of 2.5 and exhibited great pH adaptability and stability (>80% activity over pH 2.0–6.0 and pH 2.0–10.0, respectively). It had a high specific activity (1356 U/mg) against locust bean gum, was able to degrade galactomannan and glucomannan in a classical four-site binding mode, and catalyzed the transglycosylation of mannotetrose to mannooligosaccharides with higher degree of polymerization. Besides, it had great resistance to pepsin and trypsin and digested corn–soybean meal based diet in a comparable way with a commercial β-mannanase under the simulated gastrointestinal conditions of pigs. This acidophilic β-mannanase represents a valuable candidate for wide use in various industries, especially in the feed.	Irrelevant	Title and abstract are not relevant with the topic	References removed
13	Annalaura Brai, Claudia Immacolata Trivisani, Chiara Vagaggini, Roberto Stella, Roberto Angeletti, Giulia Iovenitti, Valeria Francardi, Elena Dreassi	Proteins from Tenebrio molitor: An interesting functional ingredient and a source of ACE inhibitory peptides	2022	Food Chemistry	https://doi.org/10.1016/j.foodchem.2022.133409	The angiotensin-converting enzyme (ACE) inhibitory potential of the main protein fractions from Tenebrio molitor larvae (TML) was examined to evaluate their use as a novel antihypertensive functional food. Both fractions contained YAN tripeptide, previously reported as responsible for ACE inhibition. Although YAN has been synthesized and was used as a standard for LC-MS/MS quantification and IC50 against ACE was determined, low yields of YAN from TML did not explain adequately the activity of the whole protein fraction. LC-HRMS/MS investigation led to the identification of other three peptides, which were evaluated in silico, synthesized and tested against ACE. Among them, tetrapeptide NIKY showed the most promising activity (52Â ÂµM), highlighting once more the potential of TML and paving the way for exploitation in novel foods.	Relevant	Title and abstract are relevant to the topic	References are used for the full review stage
14	Milan Dhakal, Varongsiri Kemsawasd, Kanyawee Whanmek, Wimonphan Chathiran, Saranya Intawong, Warangkana Srichamnong, Uthaiwan Suttisansanee, Suwapat Kittibunchakul	Physicochemical characteristics, volatile components and bioactivities of fermented seasoning sauce produced from cricket (Acheta domesticus) meal	2025	Future Foods	https://doi.org/10.1016/j.fufo.2024.100505	This study developed novel fermented seasoning products from house cricket meal using an accelerated process that integrated enzymatic digestion with bacterial fermentation. Two non-pathogenic food-derived bacteria, Staphylococcus piscifermentans TISTR 824 and Halobacillus sp. TISTR 1860, chosen for their ability to grow in AlcalaseÂ®-pretreated cricket broth and exhibit proteolytic activity at high salt concentrations (15 % NaCl), were employed as starter cultures for a 12-week fermentation process. The resulting fermented cricket sauce (FCS) products showed values for degree of hydrolysis (âˆ¼54.8â€"56.6 %), pH (âˆ¼5.7â€"6.2), absorbance at 420 nm (âˆ¼2.8â€"4.2) and water activity (âˆ¼0.74â€"0.75) that were similar to Thai fish sauces. The FCS products had unique volatile profiles compared to a commercial Thai fish sauce sample (CFSS) and exhibited improved antioxidant potential compared to the raw material as a competitive alternative to fish sauces. A selected FCS product with the highest antioxidant activities showed better anti-obesity and anti-diabetic effects than the CFSS and was classified as a "light in sodium" product. Given its NaCl content, this product should be stable over extended periods without refrigeration. Our results support the use of fermentation to process edible cricket products, thereby enhancing the value of cricket meal and potentially boosting the acceptance and consumption of insect-based foods.	Relevant	Title and abstract are relevant to the topic	References are used for the full review stage

15	Andressa Jantzen da Silva Lucas, EliÃ©zer Quadro Oreste, Helena LeÃ£o Gouveia Costa, HÃ©ctor MartÃ-n LÃ³pez, Carolina Dias Medeiros Saad, Carlos Prentice	Extraction, physicochemical characterization, and morphological properties of chitin and chitosan from cuticles of edible insects	2021	Food Chemistry	https://doi.org/10.1016/j.foodchem.2020.128550	As an alternative, cuticles from edible insects was proposed as an unconventional but viable source of chitin and chitosan. The chitin present in the mealwormâ€™s (Tenebrio molitor) cuticles was obtained biotechnologically in one step of enzymatic deproteinization and after deacetylated. Differences in the physicochemical characteristics and the properties of the cuticles, chitin, and chitosan were investigated in this study. Commercial chitosan was used as a reference sample to validate the methods used. The enzymatic deproteinization used to obtain chitin showed an efficiency of 85%. The global yield of the process (cuticle-to-chitosan) was 31.9%. The characterization results of these polymers using DSC, FT-IR, XRD, TGA, and SEM techniques demonstrate consistency with the degree of deacetylation of the obtained chitosan, allowing the differentiation between chitin and chitosan. This study suggests that the wastes of edible insect breeding should be collected and evaluated as an alternative of chitin/chitosan source.	Irrelevant	Title and abstract are not relevant with the topic	References removed
16	LetÃ-cia Nunes da Cruz, Liliana de Oliveira Rocha, Ruann Janser Soares de Castro	Submerged fermentation using Aspergillus tubingensis as an efficient strategy to obtain antioxidant extracts from black cricket proteins	2023	Food and Humanity	https://doi.org/10.1016/j.foohum.2023.08.012	In recent years, there has been growing interest in edible insects as a novel food source due to their high nutritional value and more sustainable cultivation system compared to traditional animal sources. Edible insects are rich in proteins, which can be used to produce bioactive peptides through enzymatic hydrolysis or fermentation. The main objective of this study was to evaluate the production of an antioxidant extract using proteins extracted from the black cricket (Gryllus assimilis) as a substrate for the submerged fermentative process conducted by the filamentous fungus Aspergillus tubingensis. A response surface methodology (CCRD) was employed to investigate the effect of glucose and black cricket protein concentrations in the culture medium on the release of the peptides. The results demonstrated that the maximum values for DPPH (290.79Â Âµmol TE gâ€“1), ABTS (862.82Â Âµmol TE gâ€“1), and FRAP (1020.11Â Âµmol TE gâ€“1) were obtained at 72 or 96Â h of fermentation using lower concentrations of both protein and glucose. These findings confirm that black cricket proteins are suitable for producing an antioxidant extract via microbial fermentation.	Relevant	Title and abstract are relevant to the topic	References are used for the full review stage
17	Xiangxiang Ni, Chengcheng Chen, Ruixi Li, Qiwei Liu, Chaoyi Duan, Xiu Wang, Mingfeng Xu	Effects of ultrasonic treatment on the structure and functional characteristics of myofibrillar proteins from black soldier fly	2024	International Journal of Biological Macromolecules	https://doi.org/10.1016/j.ijbiomac.2024.135057	In the process of utilizing black soldier fly larvae (BSFL) lipids to develop biodiesel, many by-products will be produced, especially the underutilized protein components. These proteins can be recycled through appropriate treatment and technology, such as the preparation of feed, biofertilizers or other kinds of bio-products, so as to achieve the efficient use of resources and reduce the generation of waste. Myofibrillar protein (MP), as the most important component of protein, is highly susceptible to environmental influences, leading to oxidation and deterioration, which ultimately affects the overall performance of the protein and product quality. For it to be high-quality and fully exploited, in this study, black soldier fly myofibrillar protein (BMP) was extracted and primarily subjected to ultrasonic treatment to investigate the impact of varying ultrasonic powers (300, 500, 700, 900Â W) on the structure and functional properties of BMP. The results indicated that as ultrasonic power increased, the sulfhydryl content and turbidity of BMP decreased, leading to a notable improvement in the stability of the protein emulsion system. SEM images corroborated the changes in the microstructure of BMP. Moreover, the enhancement of ultrasound power induced modifications in the intrinsic fluorescence spectra and FTIR spectra of BMP. Additionally, ultrasonic treatment resulted in an increase in carbonyl content and emulsifying activity of BMP, with both peaking at 500Â W. It was noteworthy that BMP treated with ultrasound exhibited stronger digestibility compared to the untreated. In summary, 500Â W was determined as the optimal ultrasound parameter for this study. Overall, ultrasound modification of insect MPs emerges as a dependable technique capable of altering the structure and functionality of BMP.	Irrelevant	Title and abstract are not relevant with the topic	References removed

18	Lorenzo Nissen, Seyedeh Parya Samaei, Elena Babini, Andrea Gianotti	Gluten free sourdough bread enriched with cricket flour for protein fortification: Antioxidant improvement and Volatilome characterization	2020	Food Chemistry	https://doi.org/10.1016/j.foodchem.2020.127410	Insects represent a novel source of edible high nutritional value proteins which are gaining increasing interest as an alternative to traditional animal foods. In this work, cricket flour was used to produce gluten-free sourdough breads, suitable for celiac people and â€œsource of proteinsâ€�The doughs were fermented by different methods and pH and microbial growth, volatile compounds, protein profile, and antioxidant activity, before and after baking, were analyzed and compared to standard gluten-free doughs. The results showed that cricket-enriched doughs and the standard had similar fermentation processes. Cricket enrichment conferred to the breads a typical flavoring profile, characterized by a unique bouquet of volatile compounds, made by nonanoic acid, 2,4-nonadienal (E,E), 1-hexanol, 1-heptanol, and 3-octen-2-one, expressed in different amounts depending on the type of inoculum. Finally, antioxidant activities were significantly enhanced in cricket breads, indicating that cricket powder provides to bakery gluten-free goods high nutritional value proteins and antioxidant properties.	Relevant	Title and abstract are relevant to the topic	References are used for the full review stage
19	Jia-hao Xu, Shan Xiao, Ji-hui Wang, Bo Wang, Yan-xue Cai, Wen-feng Hu	Comparative study of the effects of ultrasound-assisted alkaline extraction on black soldier fly (Hermetia illucens) larvae protein: Nutritional, structural, and functional properties	2023	Ultrasonics Sonochemistry	https://doi.org/10.1016/j.ultsonch.2023.106662	In this study, we developed an ultrasound-assisted alkaline method for extracting black soldier fly larvae protein (BSFLP). The effects of ultrasound-assisted extraction on the nutritional value, structural characteristics, and techno-functional properties of BSFLP were compared with those using the conventional hot alkali method. The results showed that ultrasound-assisted extraction significantly increased the extraction ratio of BSFLP from 55.40% to 80.37%, but reduced the purity from 84.19% to 80.75%. The BSFLP extracted by ultrasound-assisted extraction met the amino acid requirements for humans proposed by the Food and Agriculture Organization in 2013, and ultrasound-assisted extraction did not alter the limiting amino acids of the BSFLP. The ultrasound-assisted extraction increased the in vitro protein digestibility from 82.97% to 99.79%. Moreover, ultrasound-assisted extraction obtained BSFLP with a more ordered secondary structure and more loosely porous surface morphology, without breaking the peptide bonds. By contrast, the conventional hot alkaline method hydrolyzed BSFLP into smaller fragments. The effect of ultrasound-assisted extraction on the structure of BSFLP improved the solubility and emulsion capacity of BSFLP, but reduced its foaming properties. In conclusion, the results of this study suggest that ultrasound-assisted alkaline extraction could be a suitable method for extracting BSFLP and improving its nutritional value, and structural and functional properties. The findings obtained in this study could promote the wider application of BSFLP in food industry.	Relevant	Title and abstract are relevant to the topic	References are used for the full review stage
20	M Aulitto, F A Fusco, G Fiorentino, S Bartolucci, P Contursi, D Limauro	A thermophilic enzymatic cocktail for galactomannans degradation	2018	Enzyme and Microbial Technology	10.1016/j.enzmictec.2017.12.008	The full utilization of hemicellulose sugars (pentose and exose) present in lignocellulosic material, is required for an efficient bio-based fuels and chemicals production. Two recombinant thermophilic enzymes, an endo-1,4-β-mannanase from Dictyoglomus turgidum (DturCelB) and an α-galactosidase from Thermus thermophilus (TtGalA), were assayed at 80 °C, to assess their heterosynergystic association on galactomannans degradation, particularly abundant in hemicellulose. The enzymes were tested under various combinations simultaneously and sequentially, in order to estimate the optimal conditions for the release of reducing sugars. The results showed that the most efficient degree of synergy was obtained in simultaneous assay with a protein ratio of 25% of DturCelB and 75% of TtGalA, using Locust bean gum as substrate. On the other hand, the mechanism of action was demonstrated through the sequential assays, i.e. when TtGalA acting as first to enhance the subsequent hydrolysis performed by DturCelB. The synergistic association between the thermophilic enzymes herein described has an high potential application to pre-hydrolyse the lignocellulosic biomasses right after the pretreatment, prior to the conventional saccharification step.	Irrelevant	Title and abstract are not relevant with the topic	References removed

21	Laura Jenet Montiel-Aguilar, Jorge Ariel Torres-Castillo, RocÃo RodrÃguez-Servin, Adiel Berenice LÃ³pez-Flores, VÃctor Eustorgio Aguirre-Arzola, Gerardo MÃ©ndez-Zamora, Sugey Ramona Sinagawa-GarcÃa	Nutraceutical effects of bioactive peptides obtained from Pterophylla beltrani (Bolivar & Bolivar) protein isolates	2020	Journal of Asia-Pacific Entomology	10.1016/j.aspen.2020.06.006	Edible insects have been important sources of food proteins for human consumption and animal feed. In this study, a protein isolate from Pterophylla beltrani Bolivar & Bolivar, 1942 (Orthoptera: Tettigoniidae) was enzymatically processed and its nutraceutical properties were evaluated. Protein isolates were obtained from an insect flour and then was hydrolyzed for 5 h with a sequential process using pepsin and trypsin-chymotrypsin to simulate the gastric intestinal fluids. To evaluate the effect of peptide molecular size on nutraceutical properties, the peptides obtained from Total hydrolyzed (TH) were fractionated by ultrafiltration (UF) with 10 kDa and 3 kDa UF membranes giving fractions (F) with different molecular size (F < 3KDa, F < 10KDa and F > 10KDa). The inhibition assay of Angiotensin-Converting Enzyme (ACE) showed that the best treatment (P < 0.05) was the (TH) with an IC50 value of 0.5 mg/mL while the F < 3KDa was the lowest (P < 0.05) with an IC50 of 1.44 mg/mL, and the peptide size had no effect. However, an Î±-amylase inhibition was observed with an increase of the IC50 value between TH, F > 10KDa and F < 10KDa although no significative difference (P > 0.05) was found between the TH and F < 3KDa with IC50 of 0.48 and 0.68 mg/mL, respectively. In antioxidant activity, significant differences (P < 0.05) were observed between TH and UF fractions where the best response was in the F < 3KDa. In conclusion, P. beltrani proteins isolate are a source of bioactive peptide, and these could be considered as potential edible insect and sustainable food with nutraceutical effects.	Relevant	Title and abstract are relevant to the topic	References are used for the full review stage
22	L J H Sweers, C M M Lakemond, V Fogliano, R M Boom, M Mishyna, J K Keppler	Biorefining of liquid insect fractions by microfiltration to increase functionality	2024	Journal of Food Engineering	https://doi.org/10.1016/j.jfoodeng.2023.111821	Insects are gaining attention as sustainable protein sources, necessitating the evaluation of gentle processing techniques for decontamination and fractionation of edible insects. Microfiltration was shown to be an effective alternative to thermal treatments for microbial decontamination of soluble insect protein. In this study, mealworm and cricket fractions extracted at pH 3 and 8, obtained through a microfiltration (0.2Â Î¼m polyethersulphone membrane) process, were assessed. This microfiltration process yielded a cream layer, a retentate containing protein aggregates, and a decontaminated permeate containing smaller-sized proteins (mostly <75Â kDa). Microfiltration improved the permeatesâ€™ foamability to values higher than whey protein isolate (215â€“317% vs. 163%), while the retentates retained the gelling properties of up to â‰ˆ5000Â Pa. Additionally, stable lipid droplets resembling plant-based oleosomes were recovered from the cream layer. This study shows that microfiltration is promising for simultaneous decontamination and fractionation, providing distinct fractions for diverse food applications.	Irrelevant	Title and abstract are not relevant with the topic	References removed
23	Renske H Janssen, Jean-Paul Vincken, Nathalie J G Arts, Vincenzo Fogliano, Catriona M M Lakemond	Effect of endogenous phenoloxidase on protein solubility and digestibility after processing of Tenebrio molitor, Alphitobius diaperinus and Hermetia illucens	2019	Food Research International	https://doi.org/10.1016/j.foodres.2018.12.038	Upon extracting soluble proteins from insects as potential food ingredient, endogenous enzymes, such as phenoloxidases, are expected to negatively affect protein properties. The effect of phenoloxidases on solubility and digestibility of proteins was investigated for larvae of Tenebrio molitor, Alphitobius diaperinus and Hermetia illucens. Phenoloxidase inhibition was done using blanching (50â€¯s, 90â€¯Â°C) before extraction or extracting in presence of sulfite. Similar soluble protein yields and compositions were found without and with sulfite addition, whereas blanching decreased soluble protein yield. Upon in-vitro hydrolysis by pepsin and trypsin, soluble proteins from H. illucens were more digestible than those of T. molitor and A. diaperinus. Phenoloxidase activity during grinding negatively affected in-vitro pepsin hydrolysis. Besides phenoloxidase activity, also endogenous proteases were shown to remain active at pHâ€¯8 in extracts containing sulfite and after blanching of larvae. This stresses that protease activity needs to be carefully controlled in the design of insect based ingredients.	Irrelevant	Title and abstract are not relevant with the topic	References removed

24	Y Wang, P Shi, H Luo, Y Bai, H Huang, P Yang, H Xiong, B Yao	Cloning, over-expression and characterization of an alkali-tolerant endo-Î²-1,4-mannanase from Penicillium freii F63	2012	Journal of Bioscience and Bioengineering	10.1016/j.jbiosc.2012.02.005	A glycosyl hydrolase family 5 endo-β-mannanase gene (man5F63) was cloned from Penicillium freii F63 and overexpressed in Pichia pastoris. man5F63 contained an open reading frame of 1260 bp that encoded a polypeptide of 419 amino acids including a putative 18-residue signal peptide. The recombinant enzyme (rMan5F63) was secreted into the culture supernatant to near electrophoretic homogeneity with a high yield (1.1 g l–1 in flask). Its apparent molecular weight was approximately 72.0 kDa, 29.0 kDa higher than the theoretical molecular mass. rMan5F63 was optimal at pH 4.5 and 60°C and exhibited good stability over a broad pH range from acidic to alkaline (> 85.0% activity at pH 4.0–9.0, > 70.0% activity at pH 10.0 and 43.7% even at pH 12.0). The activity of rMan5F63 was significantly enhanced in the presence of Co2 +, Cu2 +, Mn2 + and β-mercaptoethanol and was strongly inhibited by Hg2 + and SDS. The specific activity, Km and Vmax values were 47.5 U mg− 1, 7.8 mg ml− 1 and 70.4 μmol min− 1 mg− 1, respectively, for locust bean gum, and 40.3 U mg− 1, 2.3 mg ml− 1 and 61.7 μmol min− 1 mg− 1, respectively, for konjac flour. All these favorable enzymatic properties make it cost-effective to commercialization and valuable in various industries.	Irrelevant	Title and abstract are not relevant with the topic	References removed
25	Yoo Hee Kim, Phuong Nguyen, Seong-Ryul Kim, Sang-Kuk Kang, Kee-Young Kim, Young Ho Koh	A comparison of nutritional components and memory enhancement effects of HongJam prepared from different silkworm varieties that weave yellow-colored cocoons	2023	Journal of Asia-Pacific Entomology	https://doi.org/10.1016/j.aspen.2023.102167	Hongjam (HJ) is a natural health food made from mature silkworms, and various health-promoting effects of HJ have been reported. Recent studies have reported that the health-promoting effects of HJ vary depending on the color of the cocoon of the silkworm variety used for its production. Recently, we reported that Golden Silk (GS), a variety of silkworm with yellow cocoons, exhibit superior memory improvement effects when HJ derived from these silkworms is compared to other varieties. However, breeding lines used to produce silkworm varieties are maintained through inbreeding, which can lead to serious side effects in the case of the GS variety. Therefore, it is necessary to investigate the nutritional components and health-promoting effects of Daehwangjam (DHJ), a recently established yellow cocoon silkworm variety.A comparative study was conducted on the nutritional components and memory-enhancing effects of GS and DHJ-HJ. We found no significant differences in nutrient composition between GS- and DHJ-HJ as determined through proximate analysis, amino acid composition analysis, fatty acid content analysis and other methods. Additionally, in a rodent model of mild cognitive impairment, DHJ-HJ proved to be equally effective as GS-HJ. Since DHJ produces larger mature silkworms than GS, it will be possible to efficiently produce HJ for the purpose of creating memory enhancement health food.	Irrelevant	Title and abstract are not relevant with the topic	References removed
26	Suwapat Kittibunchakul, Kanyawee Whanmek, Chalat Santivarangkna	Physicochemical, microbiological and nutritional quality of fermented cricket (Acheta domesticus) paste	2023	LWT	https://doi.org/10.1016/j.lwt.2023.115444	House crickets (Acheta domesticus) were processed into a seasoning paste, the so-called fermented cricket paste (FCP), by mimicking the production process of Thai fermented shrimp paste (Kapi). Whole house crickets were ground with solar salt (crickets to salt ratioÂ =Â 10:1 w/w), sun-dried to â¨¼50% moisture content and fermented at 30Â Â°C for 4 weeks using Kapi (5% w/w) as a starter culture. Results showed that salting and drying steps might contribute greatly to eradicating undesirable microorganisms by lowering the Aw of cricket from 0.9 to 0.7. The changes in physicochemical properties during FCP preparation could be attributed to the activity of lactic acid bacteria, which were predominant microorganisms in the FCP (5.66 log CFU/g DW). Overall, the FCP had similar characteristics to commercial Kapi products and should be well-preserved without refrigeration. Despite the lower overall nutritive value, the FCP possessed an improved amino acid profile and protein digestibility compared with the raw material (93.80% vs 81.91%), indicating that the FCP preparation process dealing with fermentation could enhance the protein quality of house crickets. The FCP could be used as a protein-rich seasoning and an alternative for fermented shrimp paste, thus helping to promote the consumption of insect-based foods.	Relevant	Title and abstract are relevant to the topic	References are used for the full review stage

27	Andrea Roncolini, Vesna MilanoviÄ‡, Lucia Aquilanti, Federica Cardinali, Cristiana Garofalo, Riccardo Sabbatini, Francesca Clementi, Luca Belleggia, Marina Pasquini, Massimo Mozzon, Roberta Foligni, Maria Federica Trombetta, M Naceur Haouet, M Serena Altissimi, Sara Di Bella, Arianna Piersanti, Francesco Griffoni, Anna Reale, Serena	Lesser mealworm (Alphitobius diaperinus) powder as a novel baking ingredient for manufacturing high-protein, mineral-dense snacks	2020	Food Research International	https://doi.org/10.1016/j.foodres.2020.109031	Increasing interest in consuming foods that are high in protein, vitamin, amino acid, and mineral contents is steering growth in the market for fortified snacks. The aim of the present study was to evaluate the use of lesser mealworm (Alphitobius diaperinus) powder (LP) (at 10 or 30% substitution for wheat flour) for the protein and mineral fortification of crunchy snacks (rusks). Hence, the technological, microbiological, nutritional, and sensory characteristics of the fortified rusks were evaluated. The protein content was enriched up to 99.3% in rusks with 30% substitution; moreover, a notable increase in the essential amino acids content was observed, with histidine fortification reaching up to 129.1% in rusks with 30% substitution. The incorporation of LP has led to an enrichment of almost all the minerals considered here, and especially Fe, P and Zn, with Zn showing fortification percentages of up to 300% in rusks with 30% substitution for LP. The experimental rusks showed pleasant sensory traits and low aw values. In view of the potential industrial manufacturing of insect-based rusks, the proposed product can be assigned to level 4 (validation in a laboratory environment) of the Technology Readiness Level (TRL) scale, and it is thus ready to be tested in a simulated production environment.	Irrelevant	Title and abstract are not relevant with the topic	References removed
28	C Rocha, J A Teixeira, L Hilliou, P Sampaio, M P GonÃ§alves	Rheological and structural characterization of gels from whey protein hydrolysates/locust bean gum mixed systems	2009	Food Hydrocolloids	10.1016/j.foodhyd.2009.02.005	The gelling ability of whey proteins can be changed by limited hydrolysis and by the addition of other components such as polysaccharides. In this work the effect of the concentration of locust bean gum (LBG) on the heat-set gelation of aqueous whey protein hydrolysates (10% w/w) from pepsin and trypsin was assessed at pH 7.0. Whey protein concentrate (WPC) mild hydrolysis (up to 2.5% in the case of pepsin and 1.0% in the case of trypsin) ameliorates the gelling ability. The WPC synergism with LBG is affected by the protein hydrolysis. For a WPC concentration of 10% (w/w), no maximum value was found in the G' dependence on LBG content in the case of the hydrolysates, unlike the intact WPC. However, for higher protein concentrations, the behaviour of gels from whey proteins or whey protein hydrolysates towards the presence of LBG becomes very similar. In this case, a small amount of LBG in the presence of salt leads to a big enhancement in the gel strength. Further increases in the LBG concentration led to a decrease in the gel strength.	Irrelevant	Title and abstract are not relevant with the topic	References removed

29	Ratasark Summart, Sumeth Imsoonthornruksa, Jirawat Yongsawatdigul, Mariena Ketudat-Cairns, Natteewan Udomsil	Characterization and molecular docking of tetrapeptides with cellular antioxidant and ACE inhibitory properties from cricket (Acheta domesticus) protein hydrolysate	2024	Heliyon	https://doi.org/10.1016/j.heliyon.2024.e35156	Wide-ranging bioactivities of enzymatically digested insect protein to produce peptides have been targeted for functional food development. In this study, fractionated peptides obtained from cricket (Acheta domesticus) protein hydrolysate by alcalase digestion were identified and evaluated for their bioactivities. Peptide fractions F44, F45, and F46, isolated through size exclusion chromatography, demonstrated strong cytoprotective effects on SH-SY5Y and HepG2 cells exposed to H2O2. This was evidenced by a 2-fold decrease in reactive oxygen species (ROS) accumulation in the cells and a 3-fold upregulation of genes encoding antioxidant enzymes. The F45 peptide fractions also showed chemical antioxidant activities ranging from approximately 290 to 393Â mg trolox/g peptide, measured by DPPH, ABTS, and FRAP assays. Furthermore, F45 demonstrated the highest angiotensin-converting enzyme I (ACE) inhibitory activity, 57.93Â %. F45 induced higher levels of Nrf2, SOD1, SOD2, CAT, GSR, and GPx4 gene expression in SH-SY5Y and HepG2 cells compared to cells treated with H2O2 and no peptides (pÂ <Â 0.05). Cells treated with H2O2 and F45 exhibited significantly increased antioxidant enzyme activity, including SOD, CAT, GSR, and GPx (pÂ <Â 0.05). The F45B fraction from F45 was sequenced to obtain FVEG and FYDQ tetrapeptides. Molecular docking analysis revealed their high binding affinity to cellular antioxidant enzymes (SOD, CAT, GSR, GPx1, and GPx4), an antioxidant-related protein (Keap1), and ACE. These results suggest that the novel tetrapeptides from Acheta domesticus demonstrate important biological activities, establishing them as significant cellular antioxidant activities and a potential source of antihypertensive peptides.	Relevant	Title and abstract are relevant to the topic	References are used for the full review stage
30	Abir Boukil, VÃ©ronique Perreault, Julien Chamberland, Samir Mezdour, Yves Pouliot, Alain Doyen	High Hydrostatic Pressure-Assisted Enzymatic Hydrolysis Affect Mealworm Allergenic Proteins.	2020	Molecules (Basel, Switzerland)	10.3390/molecules25112685	Edible insects have garnered increased interest as alternative protein sources due to the world's growing population. However, the allergenicity of specific insect proteins is a major concern for both industry and consumers. This preliminary study investigated the capacity of high hydrostatic pressure (HHP) coupled to enzymatic hydrolysis by Alcalase(Â®) or pepsin in order to improve the in vitro digestion of mealworm proteins, specifically allergenic proteins. Pressurization was applied as pretreatment before in vitro digestion or, simultaneously, during hydrolysis. The degree of hydrolysis was compared between the different treatments and a mass spectrometry-based proteomic method was used to determine the efficiency of allergenic protein hydrolysis. Only the Alcalase(Â®) hydrolysis under pressure improved the degree of hydrolysis of mealworm proteins. Moreover, the in vitro digestion of the main allergenic proteins was increased by pressurization conditions that were specifically coupled to pepsin hydrolysis. Consequently, HHP-assisted enzymatic hydrolysis represents an alternative strategy to conventional hydrolysis for generating a large amount of peptide originating from allergenic mealworm proteins, and for lowering their immunoreactivity, for food, nutraceutical, and pharmaceutical applications.	Relevant	Title and abstract are relevant to the topic	References are used for the full review stage

31	LÃvya Alves Oliveira, Stephanie Michelin Santana Pereira, Kelly Aparecida Dias, Stefany da Silva Paes, Mariana Grancieri, Luis Gonzalo Salinas Jimenez, Carlos Wanderlei Piler de Carvalho, Eugenio Eduardo de Oliveira, HÃ©rcia Stampini Duarte Martino, Ceres Mattos Della Lucia	Nutritional content, amino acid profile, and protein properties of edible insects (Tenebrio molitor and Gryllus assimilis) powders at different stages of development	2024	Journal of Food Composition and Analysis	https://doi.org/10.1016/j.jfca.2023.105804	Insects have great potential as ingredients for industrial purposes, providing good technological properties. This study aimed to characterize powders of Tenebrio molitor and Gryllus assimilis at two developmental stages for potential use in human nutrition. The insect powders were analyzed for their proximate composition, mineral content, carotenoids, vitamins C and E, riboflavin, niacin, amino acid profile, in vitro protein digestibility, solubility as a function of pH, foam-forming, and water and oil-holding capacity. Both powders were excellent sources of proteins (ranging from 57.36 % to 67.97 %), phosphorus (512.00â€“732Â mg/100Â g), copper (1.45â€“3.01Â mg/100Â g), iron (5.41â€“8.41Â mg/100Â g), zinc (11.62â€“25.57Â mg/100Â g), manganese (1.63â€“8.08Â mg/100Â g), good sources of magnesium (84.00â€“180.00Â mg/100Â g), and sources of potassium (624.00â€“820.00Â mg/100Â g), and niacin (1.88â€“3.21Â mg/100Â g). The proteins showed good digestibility (84.48â€“92.53 %) and increased solubility in alkaline pH (âˆ¼11). Lysine was identified as the limiting amino acid for both species analyzed (EAAS: 0.55â€“0.79). The development stage of the insects influenced their nutritional content, amino acid profile, and functional protein properties. The data obtained support the potential use of these insect powders on a large scale and contribute to selecting the development stage with the best nutritional composition.	Irrelevant	Title and abstract are not relevant with the topic	References removed
32	Aunzar B Lone, Hina F Bhat, Sunil Kumar, Mehnaza Manzoor, Abdo Hassoun, Abderrahmane AÃ¯t-Kaddour, Tanyaradzwa E Mungure, Rana Muhammad Aadil, Zuhaib F Bhat	Improving microbial and lipid oxidative stability of cheddar cheese using cricket protein hydrolysates pre-treated with microwave and ultrasonication	2023	Food Chemistry	https://doi.org/10.1016/j.foodchem.2023.136350	The study was carried out to investigate cricket protein hydrolysatesâ€™ (CPH) potential to enhance the storage stability of cheddar cheese. The cricket protein (CP) samples pre-processed with microwave (T1), ultrasonication (T2) or without pre-treatment (T0) were used for developing the CPH using alcalase enzyme (3%). Freeze-dried CPH were incorporated in the cheese samples (CPH-T1, CPH-T2 and CPH-T0) at the maximum level of 1.5% and were analysed for quality during 3Â months of storage (4Â Â±Â 1Â Â°C) compared to the control samples without CPH. The pre-treatments significantly improved the antimicrobial and antioxidant potential of the CPH. The CPH exhibited a significant positive effect on antioxidant potential, lipid stability, protein oxidation, microbial growth, and sensory quality of the cheddar cheese during storage. Digestion simulation showed a significant positive impact on the antioxidant activity of the cheddar cheese. Our results indicate the potential of CPH to enhance the quality of fat-rich foods during storage.	Relevant	Title and abstract are relevant to the topic	References are used for the full review stage

33	Carlos I Rivas-Vela, Eduardo CastaÃ±o-Tostado, Anaberta Cardador-MartÃnez, Silvia L Amaya-Llano, Gustavo A Castillo-Herrera	Subcritical water hydrolysis for the obtention of bioactive peptides from a grasshopper Sphenarium purpurascens protein concentrate	2023	The Journal of Supercritical Fluids	https://doi.org/10.1016/j.supflu.2023.105893	The effect of adding sodium bicarbonate(SB) and citric acid(CA) in the subcritical water (SW) hydrolysis process was studied to improve peptide extraction from Sphenarium purpurascens. A complete factorial(23) design was accomplished, and the effects of the temperature, pressure and modifier concentration on the degree of hydrolysis(DH), molecular distribution, antioxidant activity and Angiotensin I-converting enzyme(ACE) inhibitory activity were evaluated. A protein concentrate was obtained and hydrolyzed, showing a DH of up to 44.56Â Â±Â 0.92% with the addition of 1Â M SB at 130Â Â°C/20Â MPa; meanwhile, 38.73Â Â±Â 0.58% was observed using CA under the same conditions. The best bioactivities were found in CA treatments: IC50 of up to 4.06Â Â±Â 0.35Â mg/mL for DPPH, 330Â Â±Â 0.03Â Âµg/mL for ABTS and up to 0.148Â Â±Â 0.016Â mg/mL for the ACE inhibition activity. Moreover, differential hydrolysis was found due to the presence of modifiers and process conditions, being able to modulate the hydrolysis degree and obtaining hydrolysates with different characteristics and bioactivities.	Relevant	Title and abstract are relevant to the topic	References are used for the full review stage
34	Geon-Woo Park, Kyung-Ho Park, Sang-Gu Kim, Sang-Yun Lee	Profiles of Aroma Volatile Components in Textured Vegetable Proteins using Headspace Solid Phase Microextraction-Gas Chromatography-Mass Spectrometry	2025	Current Research in Food Science	https://doi.org/10.1016/j.crfs.2025.100999	Textured vegetable protein (TVP) is a significant alternative to meat, with its primary raw materials being soybeans, peas, rice, and wheat proteins. While advancements in technology have successfully replicated the unique texture of meat in plant-based proteins, research on the aroma profiles of these key raw materials remains limited. The subtle differences in aroma between meat and meat substitutes are yet to be fully addressed. In this study, we employed headspace solid-phase microextraction (HS-SPME) combined with gas chromatography-mass spectrometry (GC-MS), a specialized technique for the analysis of volatile aromatic compounds, to examine the volatile profiles of soybean, pea, rice, and wheat proteins. The identified volatile compounds included alcohols, aldehydes, carboxylic acids, ethers, furans, indoles, ketones, phenols, pyrans, and sulfur compounds. Based on prior research, eight compounds (hexanal, nonanal, 2-nonenal, 3-methylbutanal, benzaldehyde, 1-octen-3-ol, 3-octen-2-one, and 2-pentylfuran) were classified as off-flavors. Hexanal, a key marker, was found in the following order: rice showed the highest levels, followed by soybeans, peas, and wheat. Other major volatile components exhibited distinct ratios across the samples. These findings could assist in refining the next generation of TVPs and minimizing aroma heterogeneity.	Irrelevant	Title and abstract are not relevant with the topic	References removed
35	Giulia Leni, Augusta Caligiani, Stefano Sforza	Killing method affects the browning and the quality of the protein fraction of Black Soldier Fly (Hermetia illucens) prepupae: a metabolomics and proteomic insight	2019	Food Research International	https://doi.org/10.1016/j.foodres.2018.08.021	Insects are being explored as novel protein sources in order to overcome the future food demands connected to world growing population. Insects for food/feed uses are currently slowly killed through freezing by most insect rearing companies, and typically, enzymatic browning takes place in the insect proteins fractions. However, very little is known about the influence of these enzymatic reactions on the protein physical, chemical, nutritional and technological properties. In this work a metabolomics and proteomic study was conducted on Black Soldier Fly (Hermetia illucens) prepupae, killed by two different methods: freezing (commonly used), and blanching (with the aim to inhibit the enzymatic activities). Proton nuclear magnetic resonance (1H NMR) metabolomics demonstrated that slow killing method by freezing, compared with blanching, elicits the activation of several enzymatic pathways, among them melanisation with tyrosine consumption, energetic metabolism and lipolysis. These metabolic changes have an impact also on protein nutritional quality, with a loss of cysteine and lysine, likely involved in the process of melanisation and enzymatic browning. A strong effect was also observed on protein extractability: proteins from prepupae killed by blanching were found to be more extractable in milder conditions by chemical methods, and more prone to enzymatic digestion (97% of proteins released in solution upon proteolysis) than proteins from prepupae killed by freezing. All these data indicate that killing by blanching inhibits the browning reaction and other enzymatic changes occurring during slow killing by freezing, increasing the extractability of proteins in aqueous solutions, avoiding essential amino acid loss, and improving enzymatic digestibility.	Relevant	Title and abstract are relevant to the topic	References are used for the full review stage

36	Cansu Yay, Zeynep Ozlem Cinar, Serhat Donmez, Tugba Boyunegmez Tumer, Onur Guneser, Muge Isleten Hosoglu	Optimizing bioreactor conditions for Spirulina fermentation by Lactobacillus helveticus and Kluyveromyces marxianus: Impact on chemical & bioactive properties	2024	Bioresource Technology	https://doi.org/10.1016/j.biortech.2024.130832	This study focused on optimizing the production of fermented Spirulina (FS) products using a bioactivity-guided strategy with Lactobacillus helveticus B-4526 and Kluyveromyces marxianus Y-329 in a 3-L bioreactor. Various operating conditions, including aeration rates and pH modes, were tested. While both microorganisms thrived under all conditions, the â€œcascadeâ€�mode, controlling dissolved oxygen, enhanced protein hydrolysis and antioxidant activity, as confirmed by SDS-PAGE and DPPH/TEAC assays, respectively. Screening revealed that â€œcascadeâ€�FS significantly decreased viability of colon cancer cells (HT-29) in a dose-dependent manner, with up to a 72Â % reduction. DosesÂ â‰¤Â 500Â Î¼gÂ mLâˆ’1 of â€œcascadeâ€�FS proved safe and effective in suppressing NO release without compromising cellular viability. Additionally, â€œcascadeâ€�FS exhibited diverse volatile organic compounds and reducing the characteristic â€œseaweedâ€�aroma. These findings highlight â€œcascadeâ€�FS as a promising alternative food source with improved bioactive properties, urging further exploration of its bioactive compounds, particularly bioactive peptides.	Irrelevant	Title and abstract are not relevant with the topic	References removed
37	Zhenjiao Du, Yonghui Li	Review and perspective on bioactive peptides: A roadmap for research, development, and future opportunities	2022	Journal of Agriculture and Food Research	https://doi.org/10.1016/j.jafr.2022.100353	Bioactive peptides (BPs) possess a broad spectrum of beneficial effects, such as antioxidant, antihypertensive, antidiabetic, anticancer, anti-inflammatory, antimicrobial, and anti-aging functions. Given their rapid developments, it is important to revisit our knowledge of BPs, including their production, bioactivity, bioaccessibility, commercial availability, and the latest work in structural bioinformatics. In this paper, recent advances in BP sources, production and characterization, bioactivities, and commercial availability are briefly reviewed; critical challenges, as well as corresponding perspectives, are highlighted. In silico approaches show a great potential to accelerate BP studies and warrant further research. An integrated and comprehensive workflow for future BP screening and evaluation is proposed. There is a great demand for in vivo experiments to further understand the mechanisms of BPs and promote their commercialization. This perspective paper will be a useful reference for both academic researchers and industry partners to comprehend current developments in BPs and to design future research that connects structural bioinformatics, advanced computation methods, and wet chemistry studies.	Irrelevant	Title and abstract are not relevant with the topic	References removed
38	Yongkang Chen, Jian Zhong, Xuanqi Chen, Xiaomin Li, Haiqi Pu, Baoyang Chen, Yucai Guo, Anqi Chen, Wenjie Li, Peng Hu, Xinliang Zhu, Wei Zhao, Jin Niu	Dietary astaxanthin alleviates black soldier fly oil-induced negative changes of fatty acids content and muscle quality on Oncorhynchus mykiss via mammalian target of rapamycin and AMP-activated protein kinase pathway	2024	Animal Nutrition	https://doi.org/10.1016/j.aninu.2024.07.005	This study evaluated the effect of black soldier fly (Hermetia illucens) larvae oil (BO) produced by a novel technique, subcritical butane extraction, on the flesh quality, lipid nutrients and muscle growth of rainbow trout (Oncorhynchus mykiss) fillet, and investigated the alleviating mechanisms of dietary astaxanthin (AST) supplementation. Two hundred and forty fish (215.16Â Â±Â 2.30Â g) were distributed to three groups with four replicates. Fish were fed three experimental diets for 8 weeks: the control diet (CD diet), total fish oil of the CD diet was replaced with BO to formulate the BO100 diet, and then 1Â g/kg AST was supplemented with the BO100 diet to formulate the AST diet. Results showed that the final body weight and the sarcomere length of fillet were significantly increased and the protein phosphorylation levels of mammalian target of rapamycin (mTOR) and p70 S6 kinase were enhanced in the BO100 group compared to the CD group (PÂ <Â 0.05). However, there was a reduction in the hardness, springiness and chewiness of fillets, with a decrease in eicosapentaenoic acid (EPA) and docosahexaenoic acid (DHA) levels in the fish of the BO100 group (PÂ <Â 0.05). Supplementation of AST in the BO100 diet mitigated the impairment of springiness and chewiness and further promoted the sarcomere length of fillet (PÂ <Â 0.05). Furthermore, dietary AST partially restored the EPA and DHA content of fillet by increasing the phosphorylation levels of serine/threonine kinase (AKT) and AMP-activated protein kinase Î± (AMPKÎ±) (PÂ <Â 0.05) and activating the gene expression of unsaturated fatty acid synthesis. To conclude, BO produced by subcritical butane extraction can be a readily available oil source for rainbow trout feed that can be used to promote muscle growth in rainbow trout. Further dietary AST supplementation can alleviate BO-induced lipid accumulation, restore DHA levels and improve the flesh quality of rainbow trout fillet.	Irrelevant	Title and abstract are not relevant with the topic	References removed

39	Yizhe Zhang, Ping He, Leiman Pan, Zhengli Lin, Jinxi Yang, Hui Wu, Mengmeng Zhang	Immunomodulatory effect of earthworm protein autolysates on Cyclophosphamide(CTX)-Induced immunosuppressed mice	2023	Food Bioscience	https://doi.org/10.1016/j.fbio.2023.103297	Earthworms are rich in protein and are considered a good source of edible insect protein. However, there has been limited research on the bioactivity of earthworm protein hydrolysates. The present study investigated the immunological impact of earthworm protein autolysate (EPA) on Cyclophosphamide (CTX)-induced immunosuppressed mice model. The results revealed that the Degree of Hydrolysis (DH) and percentage of soluble peptide content of the EPA were 22.38% and 77.92%, respectively. EPA was efficient in treating CTX-dropped mice's immunosuppression as well as intestinal inflammation. The peptide sequences of the EPA digestion products were identified, and ten top-scoring peptides were obtained using the PeptideRanker and HPepDOCK assessment, whose activity was validated in the macrophage model, with WNWLLPLMLG having the best immunomodulatory activity. Therefore, EPA may be a potential resource for immunomodulatory peptides, and autolysis can be a reliable technique for preparing peptides.	Irrelevant	Title and abstract are not relevant with the topic	References removed
40	Maria Otilia Carvalho, Henrique Geirinhas, SÃ³nia Duarte, Carla GraÃ§a, Isabel de Sousa	Impact of red flour beetle infestations in wheat flour and their effects on dough and bread physical, chemical, and color properties	2023	Journal of Stored Products Research	https://doi.org/10.1016/j.jspr.2023.102095	The impact of Tribolium castaneum on the quality and technological suitability of bread-making wheat flour was evaluated. The aim of this study is to investigate whether insect-infested flours can be used, after pest removal, avoiding the common use of insecticides and flour waste. The tests were carried out with wheat flour infested red flour beetle with 500 adults/kg (N1), 1000 adults/kg (N2) and 2000 adults/kg (N3), for two weeks. Flour color, total starch, protein and water contents, mineral composition and flour acidity were studied. The technological properties of the respective doughs and of bread were characterized. The results showed that infested flours acidity was significantly higher (N1, N2 and N3Â =Â 0.3Â g H2SO4/100Â g) than the control (0.1Â g H2SO4/100Â g), the total starch content decrease, from 78Â g (control) to 70Â g/100Â gÂ dm (N3), and protein content did not significantly change, 7Â g/100Â g (control) to 8Â g/100Â g (N2); flour infested showed darker greyish tone which colour differences Î”E higher than 5, while the bread crust was lighter; the gelatinization properties of starch were slightly influenced by degree of infestation with gelatinization temperature reduction of about 5Â°c. There was not impact on the structure of the doughs from infested flours measured by rheology. The extensibility of the doughs, before and after fermentation, was moderately affected by the insects. For the respective breads, they kept their softness for longer, without significant volume change. These results encourage insect tolerance on grains and derivatives, avoiding chemical toxic insecticides and food waste. This pretends to be a contribution to pest management and decision support systems, prevention, and control of losses, relevant subject to stored products.	Irrelevant	Title and abstract are not relevant with the topic	References removed
41	Yongli Jiang, Qi Tian, Chongyang Chen, Yun Deng, Xiaosong Hu, Yunjie Yi	Impact of salting-in/out assisted extraction on rheological, biological, and digestive, and proteomic properties of Tenebrio molitor larvae protein isolates	2024	International Journal of Biological Macromolecules	https://doi.org/10.1016/j.ijbiomac.2024.137044	In this study, NaCl (salting-in) and (NH4)2SO4 (salting-out) treatments were employed in alkaline extraction and acid precipitation procedures, respectively, to enhance the extraction of Tenebrio molitor larvae protein isolates (TPIs). The in vitro digestibility, rheological properties, biological activities, and proteomic analysis of TPIs were investigated. The results showed that salting-in treatment did not result in significant differences (PÂ >Â 0.05) in antioxidant activities (except for ABTS radical scavenging), but increased tumor necrosis factor Î² (TNF-Î²) and the degree of hydrolysis (DH). Salting-out treatment significantly (P < 0.05) enhanced trichloroacetic acid-soluble peptide yield (Tsp) and ACE inhibitory activities but negatively affected antioxidant and antibacterial activities. The combined salting-in-out treatment produced the highest values of DH (45.80Â %), Tsp (72.46Â %), and TNF-Î² (0.86Â mg/kg). Proteomic analysis using UPLC-MS/MS identified 141 proteins, including metabolic enzymes and actin, in the TPIs. While the salting treatments did not significantly alter the protein compositions, they primarily affected protein content. Overall, salting-in and salting-out treatments can effectively enhance specific biological properties of T. molitor protein isolates, particularly digestibility and ACE inhibitory activities, while salting-out treatments may reduce antioxidant functions. These findings suggest the potential of salting-assisted extractions for optimizing insect protein functionality in food and nutraceutical applications.	Relevant	Title and abstract are relevant to the topic	References are used for the full review stage

42	Ha-Seong Cho, Ju-Hwi Park, Ibukunoluwa Fola Olawuyi, Ju-Ock Nam, Won-Young Lee	Optimization of ultrasound-assisted enzymatic hydrolysis Zophobas morio protein and its protective effects against H2O2-induced oxidative stress in RAW264.7 cells	2025	International Journal of Biological Macromolecules	https://doi.org/10.1016/j.ijbiomac.2025.140111	Zophobas morio protein (ZMP) is a promising protein resource with notable biological properties, and its hydrolysis could unlock enhanced bioactivities. This study investigated ultrasound-assisted enzymatic hydrolysis (UAEH) of ZMP using different enzymes (Alcalase, Neutrase, and Protamex) to determine its effect on the degree of hydrolysis (DH) compared to enzymatic hydrolysis (EH). UAEH showed greater hydrolysis efficiency than EH, with Alcalase exhibiting the highest DH. Response surface methodology (RSM) was applied to optimize UAEH conditions for Zophobas morio protein hydrolysate (ZMPH). Optimal conditions for producing ZMPH with the maximum DH were a substrate concentration of 3.52Â % (w/v), enzyme to substrate ratio of 7.64Â % (v/v), and pH of 8.35. Under the optimal condition, the maximum DH was 25.03Â %. In addition, significant structural changes in the optimized ZMPH compared to ZMP were identified, showing decreased Î±-helix and Î²-sheet content, with increased Î²-turn and unordered coil. Moreover, the optimized ZMPH demonstrated significantly improved ABTS antioxidant activity and attenuated H2O2-induced cell death in RAW264.7 cells compared to ZMP, which was attributed to better mitigation of ROS production. These findings provide an effective enzymatic hydrolysis method for producing ZMPH with significant antioxidant activity, demonstrating the potential of ultrasound-assisted hydrolysis in enhancing the bioactivity of insect proteins.	Relevant	Title and abstract are relevant to the topic	References are used for the full review stage
43	Sara Costa, SÃ³nia Pedro, Helena LourenÃ§o, Irineu Batista, BÃ¡rbara Teixeira, Narcisa M Bandarra, Daniel Murta, Rui Nunes, Carla Pires	Evaluation of Tenebrio molitor larvae as an alternative food source	2020	NFS Journal	https://doi.org/10.1016/j.nfs.2020.10.001	Edible insects have gained an increasing interest as a food product in recent years. They may represent an alternative source of protein and micronutrients compared to conventional meat sources. Thus the objective of this work was to determine the nutritional value as well as the chemical and microbiological contamination for a better evaluation of the risk/benefit of yellow mealworm larvae consumption. These larvae have a nutritional value (10.4% of fat and 13.7% of protein) similar to conventional meat sources. Their main fatty acids were oleic (37.8%) and linoleic (33.2%). The results showed these larvae fulfill the requirements for essential amino acids (all were above 100% of the daily contribution), with the exception of lysine. Our findings also showed that T. monitor larvae are an excellent source of minerals like phosphorus, magnesium and zinc (114%, 109 to 128% and 117% above RDI). In spite of having a low contribution of potassium and iron (16â€“17% and 20% of RDI), the lower sodium content is an advantage in terms of human health since high intake increases blood pressure. Mercury and lead values were below the detection limit and cadmium level was 0.10 mg/kg. Yellow mealworm larvae had high bacterial loads but no pathogenic bacteria were detected. Starvation for eight days at 5 Â°C reduced the total counts of all microorganisms, specially yeasts and total mesophilic anaerobic spore-forming bacteria.	Irrelevant	Title and abstract are not relevant with the topic	References removed
44	Benjamin Kumah Mintah, Ronghai He, Mokhtar Dabbour, Jiahui Xiang, Akwasi Akomeah Agyekum, Haile Ma	Techno-functional attribute and antioxidative capacity of edible insect protein preparations and hydrolysates thereof: Effect of multiple mode sonochemical action	2019	Ultrasonics Sonochemistry	https://doi.org/10.1016/j.ultsonch.2019.104676	Hermetia illucens (edible insect) larvae protein, and hydrolysates were prepared using three pretreatment modes (conventional, fixed-frequency ultrasonic, and sweep-frequency). Protein subunit scores, microstructure, antioxidative activity, and techno-functional property of the respective isolates and hydrolysates were investigated. Alkaline protease hydrolysis significantly enhanced protein solubility, but impaired the emulsifying property and foaming stability. Isolates and hydrolysates treated by ultrasound exhibited highest antioxidative effect, and showed excellent solubility and foam expansion over wide (2â€“12) pH, likened the conventional. Ultrasonic, particularly sweep-frequency, treated hydrolysates overall showed superior solubility, foam, and antioxidative (ABTS, Superoxide scavenging, and Ferric-reducing) capacity than the remaining modes and isolates (pâ€¯<â€¯0.05). Treatment type influenced microstructure, functional attributes and antioxidative capacity of hydrolysates and isolates. Thus, functional/antioxidative property could be improved or modified for different food applications based on elected treatment. H. illucens isolate and hydrolysate preparations thereof could suitably be used in development of novel food formulations.	Relevant	Title and abstract are relevant to the topic	References are used for the full review stage

45	Francielle Miranda de Matos, Gabriela Boscariol Rasera, Ruann Janser Soares de Castro	Multifunctional properties of peptides derived from black cricket (Gryllus assimilis) and effects of in vitro digestion simulation on their bioactivities	2024	Food Research International	https://doi.org/10.1016/j.foodres.2024.115134	Insects are a rich source of proteins and are produced in systems that have lower environmental impact. As an alternative protein source, they can be consumed directly or used as an ingredient in other formulations. Recently, there has been growing interest in utilizing insect proteins as a substrate to obtain bioactive peptides as well as in investigating the maintenance of their biological properties under physiological conditions. This study aimed to evaluate the impact of simulated digestion on the bioactive properties of protein hydrolysates from black crickets (Gryllus assimilis). Following simulated digestion of the hydrolysate obtained through the application of Flavourzyme, the scavenging activities of ABTS and DPPH radicals, and ferric reducing antioxidant power (FRAP) increased by approximately 17Â %, 246Â %, and 173Â %, respectively. For the hydrolysate obtained using the binary combination of Flavourzyme/Neutrase, the inhibitory activities of Î±-amylase and Î±-glucosidase after digestion were 47.87Â % and 12.73Â %, respectively, not significantly (pÂ >Â 0.05) different from non-digested hydrolysates. The angiotensin-converting enzyme (ACE) inhibitory activity of the sample hydrolyzed with Flavourzyme/Alcalase proteases was 42.22Â %, but this property was completely lost after in vitro digestion. Untargeted proteomic analysis allowed the identification of 22 peptides in the <3Â kDa fraction of the digested black cricket protein. The LPPLP sequence was considered potentially bioactive for all activities tested in silico.	Relevant	Title and abstract are relevant to the topic	References are used for the full review stage
46	Teresa Gonzalez-de la Rosa, Sergio Montserrat-de la Paz, Fernando Rivero-Pino	Production, characterisation, and biological properties of Tenebrio molitor-derived oligopeptides	2024	Food Chemistry	https://doi.org/10.1016/j.foodchem.2024.139400	Three protein hydrolysates from Tenebrio molitor were obtained by enzymatic hydrolysis employing two food-grade proteases (i.e. Alcalase and Flavourzyme), and a complete characterisation of their composition was done. The digestion-derived products were obtained using the INFOGEST protocol. In vitro antioxidant activity and anti-inflammatory activities were evaluated. Tenebrio molitor flour and the protein hydrolysates showed a high ability to scavenge the DPPH radical (EC50 values from 0.30 to 0.87Â mg/mL). The hydrolysate obtained with a combination of the two food-grade proteases could decrease the gene expression of pro-inflammatory genes after being digested. Furthermore, the peptidome was fully determined for the first time for T. molitor hydrolysates and digests, and 40 peptides were selected based on their bioactivity to be evaluated by in silico tools, including prediction tools and molecular docking. These results provide new perspectives on the use of edible insects as sustainable and not nutritionally disadvantageous food for human consumption.	Relevant	Title and abstract are relevant to the topic	References are used for the full review stage
47	Jiao Tan, Jing Yang, Xinyi Zhou, Ahmed Mahmoud Hamdy, Xilu Zhang, Huayi Suo, Yu Zhang, Ning Li, Jiajia Song	Tenebrio molitor Proteins-Derived DPP-4 Inhibitory Peptides: Preparation, Identification, and Molecular Binding Mechanism.	2022	Foods (Basel, Switzerland)	10.3390/foods11223626	Inhibition of dipeptidyl peptidase-4 (DPP-4) is an effective way to control blood glucose in diabetic patients. Tenebrio (T.) molitor is an edible insect containing abundant protein. T. molitor protein-derived peptides can suppress the DPP-4 activity. However, the amino acid sequence and binding mechanism of these DPP-4 inhibitory peptides remain unclear. This study used the flavourzyme for T. molitor protein hydrolysis, identified the released peptides with DPP-4 inhibitory effect, and investigated the binding interactions of these peptides with DPP-4. The results showed that flavourzyme efficiently hydrolyzed the T. molitor protein, as demonstrated by the high degree of hydrolysis, disappearance of protein bands in SDS-PAGE, and changes to protein structure. The 4-h flavourzyme hydrolysates showed a good inhibitory effect on DPP-4 (IC(50) value of 1.64 mg/mL). The fragment of 1000-3000 Da accounted for 10.39% of the total peptides, but showed the strongest inhibitory effect on DPP-4. The peptides LPDQWDWR and APPDGGFWEWGD were identified from this fraction, and their IC(50) values against DPP-4 were 0.15 and 1.03 mg/mL, respectively. Molecular docking showed that these two peptides interacted with the DPP-4 active site via hydrogen bonding, hydrophobic interactions, salt bridge formation, Ï€-cation interactions, and Ï€-Ï€ stacking. Our findings indicated that T. molitor protein-derived peptides could be used as natural DPP-4 inhibitors.	Relevant	Title and abstract are relevant to the topic	References are used for the full review stage

48	Kora Kassandra Grossmann, Michael Merz, Daniel Appel, Maria Monteiro De Araujo, Lutz Fischer	New insights into the flavoring potential of cricket (Acheta domesticus) and mealworm (Tenebrio molitor) protein hydrolysates and their Maillard products	2021	Food Chemistry	https://doi.org/10.1016/j.foodchem.2021.130336	Insect proteins have an earthy-like flavor and have not shown great flavor potential for food applications so far. In this study, insect proteins of cricket Acheta domesticus and mealworm Tenebrio molitor larvae were first enzymatically hydrolyzed using two peptidase preparations (Flavourzyme1000L and ProteaseA â€œAmanoâ€[illegible]SD). Xylose was then added to facilitate Maillard reactions (30Â min, TÂ =Â 98Â Â°C, 1% (w/v) xylose). A comprehensive sensory evaluation showed that both the hydrolysis and the Maillard reactions changed the flavor description of the samples significantly to more complex and savory-like taste profiles (27 descriptors for cricket and 39 descriptors for mealworm protein). In addition, 38 odor-active molecules were identified using gas chromatographyâ€“olfactometry (1 alcohol, 5 acids, 11 aldehydes, 5 ketones and 16 heterocyclic compounds). The results showed impressively that the flavoring potential of insect proteins was significantly enhanced with respective processing.	Relevant	Title and abstract are relevant to the topic	References are used for the full review stage
49	Zahra Tavakoli, Gholamreza Kavoosi, Roghayeh Siahbalaei, Javad Karimi	Spirulina maxima as a valuable ingredient: Determination of broad fatty acid and amino acid profiles and nutritional quality and anti-amylase capacity	2025	Applied Food Research	https://doi.org/10.1016/j.afres.2025.100741	This research studied Arthrospira (Spirulina) maxima for their approximate chemical composition, fatty acid composition, amino acid composition, protein nutritional quality, lipid nutritional quality, and anti-amylase capacity. The study on Arthrospira indicates a significantly high protein content of 45.50 %, along with 21 % carbohydrates, 17 % lipids, and 9.33 % ash content. The amino acid profile shows high levels of alanine (17.38 g), glycine (11.75 g), and glutamic acid (9.69 g) per 100 g protein. The protein quality is noteworthy, with significant amounts of protein efficiency ratio, and essential, non-essential, hydrophobic, ketogenic, branched-chain, flavor, and sulfur amino acids. Additionally, the study highlights a diverse fatty acid profile, including linolenic and palmitic acids, with a high percentage of unsaturated fatty acids (75.76 %) and polyunsaturated fatty acids (71.63 %). Given the lipid nutritional quality indices, Arthrospira had promising unsaturation, peroxidability health-promoting, omega-6/omega-3, hypocholesterolemic, nutritive value, atherogenicity, and thrombogenicity indices. Ultraviolet absorption, fluorescence quenching analysis, and Colorimetric assay revealed that protein and lipid hydrolysate interact with amylase and inhibit amylase activity. Therefore, Arthrospira can be considered a functional food with high nutritional quality and is imperative as an amylase inhibitor for diabetes management.	Irrelevant	Title and abstract are not relevant with the topic	References removed
50	S K Reddy, V BÃ¥genholm, N A Pudlo, H Bouraoui, N M Koropatkin, E C Martens, H StÃ¥lbrand	A Î²-mannan utilization locus in Bacteroides ovatus involves a GH36 Î±-galactosidase active on galactomannans	2016	FEBS Letters	10.1002/1873-3468.12250	The Bacova_02091 gene in the β-mannan utilization locus of Bacteroides ovatus encodes a family GH36 α-galactosidase (BoGal36A), transcriptionally upregulated during growth on galactomannan. Characterization of recombinant BoGal36A reveals unique properties compared to other GH36 α-galactosidases, which preferentially hydrolyse terminal α-galactose in raffinose family oligosaccharides. BoGal36A prefers hydrolysing internal galactose substitutions from intact and depolymerized galactomannan. BoGal36A efficiently releases (> 90%) galactose from guar and locust bean galactomannans, resulting in precipitation of the polysaccharides. As compared to other GH36 structures, the BoGal36A 3D model displays a loop deletion, resulting in a wider active site cleft which likely can accommodate a galactose-substituted polymannose backbone.	Irrelevant	Title and abstract are not relevant with the topic	References removed
51	Daniel E GarcÃ-a-Valle, Madai. LÃ³pez-Silva, Graciela. Santos-MartÃ-nez, Virginia. HernÃ¡ndez-PÃ©rez, Juan JosÃ©. Figueroa-GonzÃ¡lez	Chemical, structural characterization and in vitro protein digestibility of cicada (Cicadidae) flour	2024	Journal of Food Composition and Analysis	https://doi.org/10.1016/j.jfca.2024.106454	Cicada (Cididade) is an insect that has lacked exhaustive characterization despite its consumption in various regions of Mexico and other parts of the world. This study aims to determine the chemical composition of cicada (Cicadidae) flour and determine the structural characteristics and in vitro digestibility of the proteins present in cicada (Cicadidae) flour. Cicada flour exhibited a higher protein content (63.51 Â± 0.22â€¯g 100â€¯gâˆ’1 flour). FTIR analysis revealed that Î²-turns and Î²-sheets dominate the protein secondary structure in insect flour. In vitro protein digestibility was assessed using the INFOGEST protocol and demonstrated high levels of digestion, with values around 95â€¯% of hydrolysis for cicadas. These findings highlight cicada flour as a valuable protein source that offers an alternative to traditional animal protein production methods. Therefore, it can be a potential ingredient for incorporation into various food matrices.	Relevant	Title and abstract are relevant to the topic	References are used for the full review stage

52	D Azzollini, A Derossi, V Fogliano, C M M Lakemond, C Severini	Effects of formulation and process conditions on microstructure, texture and digestibility of extruded insect-riched snacks	2018	Innovative Food Science & Emerging Technologies	https://doi.org/10.1016/j.ifset.2017.11.017	Extruded cereals made of wheat flour and grinded Yellow mealworm larvae (Tenebrio molitor) were produced to investigate the effect of insect inclusion (0%, 10%, 20%) and processing conditions (barrel temperature and screw speed) on their nutritional content, microstructure, texture and digestibility. Snacks enriched with 10% mealworm powder shifted their macronutrient composition towards a protein content high enough to claim the food as â€œsource of proteinâ€according to European food regulation. At 10% of enrichment, the adoption of high barrel temperature and screw speed significantly improved the microstructure, in terms of expansion and pore structure, delivering acceptable textural qualities. At 20% substitution, snacks showed poor expansion properties, mainly due to the presence of fat in the larvae. Starch and protein digestibility of were correlated with microstructure properties as a function of porosity, pore size and wall thickness. Interestingly, mechanical forces generated in extrusion likely improved the digestibility of T. molitor proteins which are tightly bound and sclerotized to the exoskeleton. Tailoring processing conditions and formulation insect ingredients can be successfully incorporated into extruded cereal snacks. Industrial relevance This study evaluated the nutritional and technological properties of extruded cereal snacks enriched with an edible insect powder (T. molitor). Results suggested that edible insects can be used as novel ingredient in extruded snacks and pointed out how processing conditions can modulate snack digestibility.	Irrelevant	Title and abstract are not relevant with the topic	References removed
53	S Kang, C.-H. Chung	Characteristics of Tenebrio molitor larvae and Bombyx mori pupae sequentially fermented with Aspergillus oryzae and Bacillus subtilis	2022	Korean Journal of Food Science and Technology	10.9721/KJFST.2022.54.1.94	Defatted soybean, larvae of brown mealworm (Tenebrio molitor), and powdered pupae of silkworm (Bombyx mori) were fermented in solid and liquid forms using Aspergillus oryzae and Bacillus subtilis. The protein degradation rate (NDR) through solid fermentation was the highest in the fermented soybean control sample (54.69±6.54%), followed by silkworm pupae (34.82±5.99%) and brown mealworm larvae (30.54±3.80%). When these edible insects were fermented in liquid form, solid extraction yield was 37.73-46.88%, and protein yield was 47.47-63.02%. NDR of fermented liquid form products increased to 58.90, 52.62, and 50.13% for soybean, brown mealworm larvae, and silkworm pupae, respectively. SDS-PAGE of the liquid fermented products confirmed that microbial fermentation decomposed higher-molecular-weight proteins into small polypeptides. In vitro digestibility of liquid forms of edible insects increased by 1.26 to 1.53 times after fermentation. The protein solubility, foaming ability, and foam stability of liquid-fermented edible insects all tended to increase through fermentation.	Irrelevant	Title and abstract are not relevant with the topic	References removed
54	C. Azagoh, F. Ducept, R. Garcia, L. Rakotozafy, M. E. Cuvelier, S. Keller, R. Lewandowski, S. Mezdour	Extraction and physicochemical characterization of Tenebrio molitor proteins	2016	Food Research International	10.1016/j.foodres.2016.06.010	This study focused on the extraction and physicochemical characterization of proteins from larvae and larvae meal of Tenebrio molitor. The larvae were subjected to a protein extraction process which involved a thermo-mechanical pre-treatment to produce the larvae meal. Soluble proteins from larvae and from larvae meal were subsequently extracted by solubilisation at an alkaline pH. The products obtained were then characterized and compared. The larvae and larvae meal were rich in protein (65.6% and 71.6% respectively) and displayed good essential amino acid (EAA) profiles. They contained all EAA and in sufficient quantities to meet the dietary requirements of both humans and salmon, except for a deficiency in methionine. The EAA profile of the larvae meal was also comparable to those of fish and soya meals used for feed. At pHÂ 10 and 45Â Â°C, the protein extraction yield of larvae (59.9%) was two-fold that of larvae meal (26.4%). The soluble proteins had protein contents on dry matter of 84% and 80% from larvae and larvae meal respectively. Molecular weights ranged from â‰¤Â 14 to 100Â kDa but the two soluble proteins differed. The soluble proteins had a solubility which was highly pH-dependent, with a low solubility at pHÂ 3 to 5. Their surface charge depended on both the pH (in particular) and the NaCl concentration. The surface hydrophobicity at pHÂ 7 of soluble proteins from larvae (670.3) was higher than that of soluble proteins from larvae meal (102.5). These soluble proteins lowered the water surface tension to 42Â mN/m and 32Â mN/m for the soluble proteins from larvae and from larvae meal respectively. Chemical compounds used in this work. Glycine (PubChem CID: 750); Glycerol (PubChem CID: 753); Tris-(hydroxymethyl)aminomethane (PubChem CID: 4468930); Sodium chloride (PubChem CID: 5234); Ethanol (PubChem CID: 702); Monosodium phosphate (PubChem CID: 23672064); Disodium hydrogen phosphate (PubChem CID: 24203); 2-mercaptoethanol (PubChem CID: 1567); Hydrochloric acid (PubChem CID: 313); Bromophenol blue (PubChem CID: 8272); Sodium hydroxide (PubChem CID: 14798); Sodium dodecyl sulphate (PubChem CID: 3423265).	Relevant	Title and abstract are relevant to the topic	References are used for the full review stage

55	Aysen Bas, Sedef Nehir El	Nutritional evaluation of biscuits enriched with cricket flour (Acheta domesticus)	2022	International Journal of Gastronomy and Food Science	https://doi.org/10.1016/j.ijgfs.2022.100583	The need for alternative protein sources has received considerable interest in insect-based foods. In this study, wheat flour in the standard biscuit recipe was replaced with 20% cricket (Acheta domesticus) flour. Compared with standard biscuits, cricket biscuits did not differ in physical properties, but they differed in protein, dietary fiber, ash, and carbohydrate content. The estimated glycemic index of standard and cricket biscuits was 49.28 and 47.84, respectively. The in vitro protein digestibility of cricket biscuits (45.19%) was almost two times that of standard biscuits (26.47%. There is not significant difference in lipid digestibility between standard (57.74%) and cricket (55.56%) biscuits (pÂ >Â 0.05). In addition, hydroxymethylfurfural was not detected. Furfural was 0.22 and 0.38Â ppm, and acrylamide was 111.10 and 122.01Â ppb in standard and cricket biscuits, respectively. Cricket biscuits can be considered as a suitable option for snack standards because of their high protein with nutritional and health claims.	Relevant	Title and abstract are relevant to the topic	References are used for the full review stage
56	Ricky Wang, Taner Sar, Amir Mahboubi, Rikard Fristedt, Mohammad J Taherzadeh, Ingrid Undeland	In vitro protein digestibility of edible filamentous fungi compared to common food protein sources	2023	Food Bioscience	https://doi.org/10.1016/j.fbio.2023.102862	Edible filamentous fungi, as a source of mycoprotein, is an emerging sustainable protein source as it can be cultivated on food-industry sidestreams, thus providing the food system with circularity. However, the digestibility of mycoprotein from different species of fungi is yet to be studied and compared to commonly consumed food proteins derived from muscle. Using the static INFOGEST in vitro gastrointestinal (GI) digestion protocol, but with less pancreatin than the recommended amount to omit high background from enzyme autolysis, this study investigated the protein degree of hydrolysis (DH%) and amino acid accessibility of five species of edible fungi in comparison with salmon fillet, chicken breast, beef tenderloin and casein. Three of the edible fungi species reached protein DH% between 58%Â Â±Â 2.6% and 62%Â Â±Â 5.6% during GI digestion compared to chicken, salmon, and beef reaching 62%â€“67% as well as casein at 55%. The amino acid accessibility of fungi (81%â€“92%), was comparable to that of salmon, chicken breast, and beef (90%â€“94%). This study thus indicated that edible fungi is a sustainable and nutritionally sound protein source.	Irrelevant	Title and abstract are not relevant with the topic	References removed
57	Sang-Dock Ji, Phuong Nguyen, Sun-Mi Yoon, Kee-Young Kim, Jong Gon Son, Hae-young Kweon, Young Ho Koh	Comparison of nutrient compositions and pharmacological effects of steamed and freeze-dried mature silkworm powders generated by four silkworm varieties	2017	Journal of Asia-Pacific Entomology	https://doi.org/10.1016/j.aspen.2017.10.010	Abstracts The mulberry silkworm, Bombyx mori, has provided valuable fabrics and foods to humans for >5000years. We invented the protocol for processing mature silkworms, which contain various functional substances, into edible steamed and freeze-dried mature silkworm powders (SMSPs). However, previously reported technique could not create powders smaller than 0.1mm and had problems with loss of product due to large SMSP particles sticking to hammer mill machines during the pulverizing process. To resolve these problems, we invented a new pulverization protocol that could create particle sizes smaller than 0.01mm. Reduced particle sizes in SMSPs offered several advantages: increased nutrient contents in the nutritional aspect and extended life expectancy and enhanced locomotor activity in the pharmacological aspect. In addition, four SMSPs were enriched with flavonoids, poly-phenols, and vitamins that are known to act as oxidative stress inhibitors in cells and tissues. Nutrient and phytochemical composition analysis results suggested that this is why SMSPs extended healthspans and inhibited onset of Parkinson's disease. Although most nutrient and phytochemical compositions among the four SMSPs were comparable, certain nutrients and chemicals were significantly higher in certain SMSPs. Therefore, further research using these four SMSPs will identify the specific health-promoting effects of each SMSP, resulting in the further development of sericulture and entomology.	Irrelevant	Title and abstract are not relevant with the topic	References removed
58	K D MartÃ-nez, M E FarÃ-as, A M R Pilosof	Effects of soy protein hydrolysis and polysaccharides addition on foaming properties studied by cluster analysis	2011	Food Hydrocolloids	10.1016/j.foodhyd.2011.03.005	The objective of the work was to study foaming properties (foam overrun, drainage rate and collapse stability) of soy protein and their hydrolysates as affected by polysaccharides. As starting material a sample of commercial soy protein isolate was used (SP) and hydrolysates of 0.4, 5.0 and 5.2% degree of hydrolysis (DH) were produced by an enzymatic reaction. The polysaccharides added were xanthan, λ and κ-carrageenan, guar, locust bean gum and hydroxypropylmethylcelluloses as surface-active polysaccharides. The effect of polysaccharides addition on foaming properties depended in a complicated way on the degree of hydrolysis of protein, surface-activity of polysaccharide, concentration of both macromolecules, contribution of polysaccharide consistency to bulk viscosity and interfacial interactions between biopolymers. However, through cluster analysis the best combination of protein/polysaccharide to obtain defined foaming properties could be determined for an eventual industrial application.	Irrelevant	Title and abstract are not relevant with the topic	References removed

59	Zhaoyang Song, Yinjuan Cao, Haijun Qiao, Pengcheng Wen, Guozheng Sun, Weibing Zhang, Ling Han	Analysis of the effect of Tenebrio Molitor rennet on the flavor formation of Cheddar cheese during ripening based on gas chromatography-ion mobility spectrometry (GC-IMS)	2023	Food Research International	https://doi.org/10.1016/j.foodres.2023.113074	This study aimed to evaluate the potential application of Tenebrio Molitor rennet (TMR) in Cheddar cheese production, and to use gas chromatography-ion mobility spectrometry (GC-IMS) to monitor flavor compounds and fingerprints of cheese during ripening. The results indicated that Cheddar cheese prepared from TMR (TF) has fat content significantly lower than that of commercial rennet (CF) (pÂ <Â 0.05). However, the results of the sensory evaluation showed that there were no statistically significant differences between the two kinds of cheese (pÂ >Â 0.05). Both cheeses were rich in free amino acids and free fatty acids. Compared to the CF cheese, gamma-aminobutyric acid and Ornithine contents of the TF cheese reached 187 and 749Â mg/kg, respectively, during 120Â days of ripening. Moreover, GC-IMS provided information on the characteristics of 40 flavor substances (monomers and dimers) in the TF cheese during ripening. Only 30 flavor substances were identified in the CF cheese. The fingerprint of the two kinds of cheese during ripening can be established by GC-IMS and principal component analysis based on the identified flavor compounds. Therefore, TMR has potential application in Cheddar cheese production. GC-IMS might be applied for the quick, accurate and comprehensive monitoring of cheese flavor during ripening.	Irrelevant	Title and abstract are not relevant with the topic	References removed
60	Legesse Shiferaw Chewaka, Chan Soon Park, Youn-Soo Cha, Kebede Taye Desta, Bo-Ram Park	Enzymatic Hydrolysis of Tenebrio molitor (Mealworm) Using Nuruk Extract Concentrate and an Evaluation of Its Nutritional, Functional, and Sensory Properties.	2023	Foods (Basel, Switzerland)	10.3390/foods12112188	Enzymatic protein hydrolysis is a well-established method for improving the quality of dietary proteins, including edible insects. Finding effective enzymes from natural sources is becoming increasingly important. This study used nuruk extract concentrate (NEC), an enzyme-rich fermentation starter, to produce protein hydrolysate from defatted Tenebrio molitor (also called mealworm, MW). The nutritional, functional, and sensorial properties of the hydrolysate were then compared to those obtained using commercial proteases (alcalase and flavourzyme). The protease activities of the crude nuruk extract (CNE), NEC, alcalase, and flavourzyme were 6.78, 12.71, 11.07, and 12.45 units/mL, respectively. The degree of hydrolysis and yield of MW hydrolysis by NEC were 15.10 and 35.92% (w/w), respectively. MW hydrolysate was obtained using NEC and had a significantly higher free amino acid content (90.37 mg/g) than alcalase (53.01 mg/g) and flavourzyme (79.64 mg/g) hydrolysates. Furthermore, the NEC hydrolysis of MW increased the antioxidant and angiotensin-converting enzyme inhibitory activity, with IC(50) values of 3.07 and 0.15 mg/mL, respectively. The enzymatic hydrolysis also improved sensory properties, including umaminess, sweetness, and saltiness. Overall, this study found that the NEC hydrolysis of MW outperformed commercial proteases regarding nutritional quality, sensory attributes, and biological activity. Therefore, nuruk could potentially replace commercial proteases, lowering the cost of enzymatic protein hydrolysis.	Relevant	Title and abstract are relevant to the topic	References are used for the full review stage
61	M L J Wessels, D Azzollini, V Fogliano	Frozen storage of lesser mealworm larvae (Alphitobius diaperinus) changes chemical properties and functionalities of the derived ingredients	2020	Food Chemistry	https://doi.org/10.1016/j.foodchem.2020.126649	The effect of frozen storage on the chemical properties and ingredient functionalities of Lesser mealworms was investigated at âˆ’20Â Â°C for 2Â months. Major changes occurred in the first week of frozen storage. Proteins, among which heavy chain myosin, underwent denaturation and aggregation, as shown by a decrease in solubility, SDS-PAGE pattern, and Confocal Laser Scanning Microscopy. The ice melting point in larvae was âˆ’32.5Â Â°C as determined by DSC: 25% of water is not frozen at âˆ’20Â Â°C, possibly due to anti-freezing proteins preventing ice formation. The presence of unfrozen water favoured various enzymatic activities as shown by a pH decrease, indicating protein hydrolysis. The molecular changes during frozen storage increased the browning reactions due to phenoloxidase activity. Foaming ability, foam stability and gel network stability increased upon frozen storage due to protein denaturation. Results provide important information regarding the opportunity of frozen storage of insect larvae for both research and industrial purposes.	Irrelevant	Title and abstract are not relevant with the topic	References removed

62	Muhammad Yusuf Abduh, Marcelino Putra Perdana, Muhammad Arifuddin Bara, Lela Wahyu Anggraeni, Ramadhani Eka Putra	Effects of aeration rate and feed on growth, productivity and nutrient composition of black soldier fly (Hermetia illucens L.) larvae	2022	Journal of Asia-Pacific Entomology	https://doi.org/10.1016/j.aspen.2022.101902	Black soldier fly larvae (Hermetia illucens L.) are widely cultivated as bioconversion agents. These larvae are known to be highly sensitive to changes in the external environment, such as temperature and relative humidity. Therefore, a rearing hive equipped with an instrumentation system known as the Modular Fly Hive was designed to cultivate black soldier larvae. This study aimed to determine the effects of aeration rate (0 â€“ 0.84Â m3/s.) and feed type (coconut endosperm waste and soybean curd) on growth and productivity of black soldier fly cultivated in Modular Fly Hives as well as well as feed digestibility and nutrient composition of the larval biomass. Increasing aeration rate decreased the average temperature and relative humidity within the hives. The results showed that the larvae demonstrated a sigmoidal growth curve for all types of feed and the type of feed significantly affects the average wet weight and length of the larvae. The greatest dry biomass productivity (52.85Â Â±Â 3.85Â g/m2/day) was observed when the larvae were cultivated using soybean curd residue with an aeration rate of 0.84Â m3/s, while the lowest dry biomass productivity (35.27Â Â±Â 9.72Â g/m2/day) was observed when the larvae were cultivated using coconut endosperm waste without aeration. Proximate analysis revealed that the larval biomass had total protein, lipid, ash and carbohydrate content in the range of 37.20 â€“ 48.60%, 9.61 â€“ 20.02%, 4.80 â€“ 6.40%, 33.86 â€“ 38.70%, respectively. Amino acid in the biomass were dominated by glutamic acid (11.11â€“12.30%), aspartic acid (8.25 â€“ 10.35%), leucine (8.09 â€“ 8.57%), and lysine (6.74 â€“ 8.14%). Lipid isolated from the larval biomass were mainly composed of lauric acid (28.35â€“61.68%), linoleic acid (6.27 â€“ 30.29%), palmitic acid (7.62 â€“ 15.23%), and myristic acid (5.05 â€“ 14.34%).	Irrelevant	Title and abstract are not relevant with the topic	References removed
63	Jin-Zheng Wang, Cheng-Kun Wu, Xin-Ying Wang, Yuan-Zhi Xu, Richard Ansah Herman, Sheng Sheng, Shuai You, Jun Wang, Fu-An Wu	One positive part of copper ion on biocatalysts: CNPs@Cu-Trypsin-assisted aqueous extraction technique for simultaneous exploration of edible insect oil and protein	2023	Chemical Engineering Journal	https://doi.org/10.1016/j.cej.2023.142560	Edible insects are rich in oil and protein resources, and has it become increasingly important to develop them reasonably when resources are increasingly scarce. However, due to the challenges in recovering enzymes in the enzyme-assisted aqueous extraction technique and the remaining organic solvents in the extraction process based on organic solvent, it is difficult to achieve simultaneous utilization of oil and protein from edible insects. Hence, an improved enzyme-assisted aqueous extraction process based on the assistance of chitosan cross-linked Fe3O4 nanoparticles (CNPs) immobilized enzymes and divalent copper ions chelation, CNPs@Cu-Trypsin-assisted aqueous extraction technique, was first proposed. The hydrolysis activity was significantly increased by 6.16 times, and the value of Kcat/Km was increased by 1.7 times for the results of the formation of the hydrophilic microenvironment. Furthermore, the extraction ratio of silkworm pupae oil extracted via CNPs@Cu-Trypsin-assisted aqueous extraction technique could be increased to 93%, and the oil extraction rate could be maintained above 65% after 10 times reused. Improvement of enzyme-assisted aqueous extraction under the addition of divalent copper ions could achieve an ideal effect of oil extracted from silkworm pupae with less time. Additionally, the quality of silkworm pupae oil was significantly improved and the silkworm pupae protein hydrolysate also showed improved biological activity. Therefore, the enzyme-assisted aqueous extraction enhancement by trypsin immobilization and divalent copper ion chelation is a promising strategy for the green and sustainable exploration of edible insect oil and protein without the use of organic solvents.	Irrelevant	Title and abstract are not relevant with the topic	References removed
64	George J Dias, Thilanka N Haththotuwa, David S Rowlands, Martin Gram, Alaa El-Din A Bekhit	Wool keratin â€“ A novel dietary protein source: Nutritional value and toxicological assessment	2022	Food Chemistry	https://doi.org/10.1016/j.foodchem.2022.132436	Keratin derived protein (KDP) was extracted from sheep wool using high pressure microwave technology and food acids and investigated for its potential as a novel dietary protein. The proximate composition, amino acid profile, element profile, in vitro cytotoxicity and digestibility of KDP were evaluated. Nutritive effects of KDP at 50% dietary supplementation were compared with a casein-based diet in a growing rat model for 95Â days. Results indicate KDP to be rich in protein (86%), amino acid cysteine (8.8Â g/100Â g) and element selenium (0.29Â Î¼g/g). KDP was non-cytotoxic in vitro atÂ â‰¤Â 2Â mg/mL concentration. There were no differences in the ratâ€™s weight gain compared to the control group (PÂ >Â 0.05). Overall, the inclusion of the KDP in the diet was an effective substitute for casein protein at 50% and KDP has the potential to be used in the food industry as a novel dietary protein, free of fat and carbohydrate.	Irrelevant	Title and abstract are not relevant with the topic	References removed

65	VÃctor M VillaseÃ±or, Jhony Navat Enriquez-Vara, Judith E UrÃas-Silva, Eugenia del Carmen Lugo-Cervantes, Diego A Luna-Vital, Luis Mojica	Mexican grasshopper (Sphenarium purpurascens) as source of high protein flour: Techno-functional characterization, and in silico and in vitro biological potential	2022	Food Research International	https://doi.org/10.1016/j.foodres.2022.112048	The aim of this work was to evaluate the techno-functional properties of Mexican grasshopper flour with different thermal pretreatments, as well as to assess the anti-inflammatory and antioxidant potential of their protein hydrolysates. Insect flour was thermally treated at 70, 80, 90, and 121Â Â°C. Insect flour protein solubility (184.3 â€“ 278.5Â mg/g) was higher at pH 7.0 â€“ 11.0. Thermally processed flour at 70, 80 and 90Â Â°C showed no significant differences (pÂ >Â 0.05) in water/oil holding capacity, emulsion properties and gel minimum concentration. Protein hydrolysates presented antioxidant potential for DPPH (IC50: 0.78Â mg/mL), ABTS (IC50: 0.63Â mg/mL) and nitric oxide (IC50: 2.21Â mg/mL). Protein hydrolysates showed inhibition potential against enzymes COX-2 (IC50: 0.52Â mg/mL) and iNOS (IC50: 0.51Â mg/mL) in biochemical trials. Molecular docking showed that from the 37 identified peptide sequences, GPPGPAGV (-9.5Â kcal/mol) and KPTVGVVTY (-10.4Â kcal/mol) have the lower binding energies for COX-2 and iNOS, respectively. S. purpurascens flour and protein hydrolysates could be used as a functional ingredient.	Relevant	Title and abstract are relevant to the topic	References are used for the full review stage
66	S Lee, Y.-S. Choi, K Jo, T.-K. Kim, H I Yong, S Jung	Quality characteristics and protein digestibility of Protaetia brevitarsis larvae	2020	Journal of Animal Science and Technology	10.5187/jast.2020.62.5.741	Herein, the in vitro protein digestibility of lyophilized Protaetia brevitarsis larvae flour with and without defatting using 70% ethanol was compared with beef loin. Proximate analysis showed that the defatted larvae contained the highest protein content ($p < 0.05$). The viable counts of total aerobic bacteria, Escherichia coli, and coliform bacteria decreased significantly after defatting the larval samples with 70% ethanol ($p < 0.05$). Measurement of α-amino group content and sodium dodecyl sulfate-polyacrylamide gel electrophoresis (SDS-PAGE) revealed higher amounts of low molecular weight proteins in the larvae compared to beef loin ($p < 0.05$). After in vitro digestion, the degree of protein hydrolysis of the digesta was higher for both larvae samples compared to beef loin ($p < 0.05$). No change was observed in the in vitro larval protein digestibility after defatting. These results highlight the excellent protein digestibility of P. brevitarsis larvae with high protein content. Defatting insect flour with 70% ethanol could enhance microbial safety while maintaining excellent protein digestibility.	Relevant	Title and abstract are relevant to the topic	References are used for the full review stage
67	N MuÃ±oz-Seijas, H Fernandes, B FernÃ¡ndez, J M DomÃnguez, J M Salgado	Eco-friendly technologies for obtaining antioxidant compounds and protein hydrolysates from edible insect Tenebrio molitor beetles	2025	Food Chemistry	10.1016/j.foodchem.2024.141726	The functional properties of edible insects can be explored by a joint use of novel technologies. This work applied varied pre-treatments (ultra-sound-assisted extraction, UAE; microwave-assisted extraction, MAE; temperature-assisted extraction, TAE; CO2-assisted extraction) and solvents (water, ethanol, water:ethanol) in Tenebrio molitor beetles to enhance the extraction of phenolic compounds with antioxidant activity. An enzymatic hydrolysis (EH) was performed in wet and treated biomasses to determine the protein hydrolysis. Higher phenolic compounds and antioxidant activity was released after MAE using water as solvent compared to the other treatments and solvents. Treatments decreased 32 %, 19 % and 30 % the protein, chitin and lipids content. EH improved protein and amino acids hydrolysis in the MAE-treated insects, followed by UAE and TAE treatments. In conclusion, MAE was the most effective to release phenolics and antioxidant activity from T. molitor beetles, while using MAE followed by EH improved protein and amino acids hydrolysis, envisioning valuable applications for this insect biomass. Â© 2024 The Authors	Relevant	Title and abstract are relevant to the topic	References are used for the full review stage
68	MarÃa RodrÃguez-RodrÃguez, Fernando G Barroso, Dmitri Fabrikov, MarÃa JosÃ© SÃ¡nchez-Muros	In Vitro Crude Protein Digestibility of Insects: A Review.	2022	Insects	10.3390/insects13080682	The high protein content of insects has been widely studied. They can be a good food alternative, and therefore it is important to study the effect of digestion on their protein. This review examines the different in vitro protein digestibility methodologies used in the study of different edible insects in articles published up to 2021. The most important variables to be taken into account in in vitro hydrolysis are the following: phases (oral, gastric and intestinal), enzymes, incubation time and temperature, method of quantification of protein hydrolysis and sample preprocessing. Insects have high digestibility data, which can increase or decrease depending on the processing of the insect prior to digestion, so it is important to investigate which processing methods improve digestibility. The most commonly used methods are gut extraction, different methods of slaughtering (freezing or blanching), obtaining protein isolates, defatting, thermal processing (drying or cooking) and extrusion. Some limitations have been encountered in discussing the results due to the diversity of methodologies used for digestion and digestibility calculation. In addition, articles evaluating the effect of insect processing are very limited. It is concluded that there is a need for the standardisation of in vitro hydrolysis protocols and their quantification to facilitate comparisons in future research.	Irrelevant	Title and abstract are not relevant with the topic	References removed

69	Giuseppe Montevecchi, Fabio Licciardello, Francesca Masino, Lucian T Miron, Andrea Antonelli	Fortification of wheat flour with black soldier fly prepupae. Evaluation of technological and nutritional parameters of the intermediate doughs and final baked products	2021	Innovative Food Science & Emerging Technologies	https://doi.org/10.1016/j.ifset.2021.102666	Bread wheat flour presents specific nutrient deficiencies, such as some essential amino acids and this drawback could be tackled by adding ingredients that contain them in high amounts. Therefore, this study aims at developing new types of flours as well as at analysing enriched flours and baked derived products, which combine the compositional and sensory characteristics of wheat bread with some peculiar nutritional properties of black soldier fly prepupae (Hermetia illucens). These composite flours were obtained by mixing â€œItalian type 1â€semi-whole wheat flour (WÂ =Â 300) with flour obtained from prepupae of black soldier fly (20Â g and 40Â g/1Â kg composite flour). Chemical, physical, and rheological analyses were carried out for each of the flour mixtures. Moisture, ash, gluten, total protein content, falling number, strength, tenacity, extensibility, amino acid profile of doughs and breads were analysed as well. An increase in the content of essential amino acids in doughs and baked products was obtained and also led to an improvement in bread texture.	Irrelevant	Title and abstract are not relevant with the topic	References removed
70	K D MartÃ-nez, C C SÃ¡nchez, V P RuÃ-z-Henestrosa, J M RodrÃ-guez Patino, A M R Pilosof	Effect of limited hydrolysis of soy protein on the interactions with polysaccharides at the air-water interface	2007	Food Hydrocolloids	10.1016/j.foodhyd.2006.09.008	The objective of the work was to study the effect of limited hydrolysis of soy protein on the interactions with polysaccharides with and without surface activity at the air–water interface at neutral pH where a limited incompatibility between macromolecules can occur. The surface pressure and phase angle as a function of time were evaluated with a drop tensiometer at 20 °C, pH 7 and ionic strength 0.05 M. Hydrolysates of 2% (H1) and 5.4% (H2) degree of hydrolysis (DH) with neutral protease from Aspergillus oryzae were obtained from a commercial soy protein isolate. The polysaccharides used were: hydroxypropylmethylcellulose (HPMC) as surface active polysaccharide; lambda carrageenan (λC) and locust bean gum (LB) as non-surface active polysaccharides. It was found that increasing DH decreased the surface pressure and increased film viscoelasticity (determined as the phase angle, θ) of soy protein hydrolysates and the nature of protein–polysaccharide interactions was strongly affected by DH. The presence of polysaccharides led to an increase of surface pressure of H1 but when added to H2, HPMC and λC decreased the surface pressure. The less hydrolyzed protein H1 gave rise to a higher surface pressure and film viscoelasticity in combination with the polysaccharides. This result points out that a limited protein hydrolysis was sufficient to improve the surface properties of soy proteins if used in combination with polysaccharides. Polysaccharides used in admixture with hydrolyzed soy proteins could control and improve the stability of foams and emulsions not only by increasing bulk viscosity but also by improving film viscoelasticity.	Irrelevant	Title and abstract are not relevant with the topic	References removed
71	M J Bidochka, G G Khachatourians	Protein hydrolysis in grasshopper cuticles by entomopathogenic fungal extracellular proteases	1994	Journal of Invertebrate Pathology	10.1006/jipa.1994.1002	Several regions of unsclerotized (teneral) cuticle from the migratory grasshopper, Melanoplus sanguinipes, were treated with extracellular protease-containing culture supernatants of the entomopathogenic fungi, Beauveria bassiana or Metarhizium anisopliae, or purified extracellular B. bassiana protease. Treatment of the various cuticles with the culture supernatants resulted in a 41.1% (forewing) to 83.0% (hindwing) loss in cuticle dry weight. Addition of phenylmethylsulfonyl fluoride (PMSF), a protease inhibitor, to the culture supernatants, resulted in a substantial retention of cuticle dry weight after the treatment. The urea-soluble proteins from untreated cuticles or cuticles treated with B. bassiana or M. anisopliae culture supernatants or purified B. bassiana protease were characterized by two-dimensional (2D) gel electrophoresis. An initial reduction in the number of acidic proteins was observed in the 2D gels from cuticles previously treated with fungal culture supernatants or purified B. bassiana protease. High-molecular-weight (>31 kDa) basic cuticular proteins were also susceptible to degradation by proteases. Addition of PMSF to the B. bassiana or M. anisopliae supernatants previous to incubation with the cuticle resulted in only minor qualitative changes in cuticular protein 2D patterns. The action of B. bassiana or M. anisopliae proteases toward acidic cuticular proteins, and to a lesser extent the high-molecular basic proteins, is discussed in light of what is known of the biochemistry of entomopathogenic fungal proteases.	Relevant	Title and abstract are relevant to the topic	References are used for the full review stage

72	Aymar Rodrigue Fogang Mba, Germain Kansci, MichÃ¨le Viau, Nordine Hafnaoui, Anne Meynier, Gustave Demmano, Claude Genot	Lipid and amino acid profiles support the potential of Rhynchophorus phoenicis larvae for human nutrition	2017	Journal of Food Composition and Analysis	https://doi.org/10.1016/j.jfca.2017.03.016	In view of future use of insects in the diet, Rhynchophorus phoenicis larvae were evaluated for their potential as protein and lipid sources. Their lipid and protein contents represented 21.35Â±2.01g/100g and 8.18Â±0.44g/100g fresh weight (FW), respectively, with energy content of 940.0kJ (224.9kcal)/100g FW. Indispensable amino acids were in higher amounts than in Food and Agriculture Organization (FAO) reference protein, with remarkably high scores for tryptophan and sulfur amino acids. Total lipids comprised neutral lipids (97.53Â±0.05g/100g lipid), glycolipids (0.59Â±0.04g/100g), phospholipids (1.88Â±0.06g/100g) and tocopherols (146Â±13Î¼g/g). Total and neutral lipids had palmitic and oleic acids (38 and 46g/100g total fatty acids, respectively) as main fatty acids, and palmitoyl, dioleylglycerol (POO) (36.4Â±0.3%) and dipalmitoyl, oleylglycerol (PPO) (30.3Â±0.3%) as main triacylglycerol molecular species. This composition explains the lipid melting/crystallization profile. Polyunsaturated fatty acids represented 0.76Â±0.17g/100g fresh larvae (linoleic acid: 0.58Â±0.11g/100g; alpha-linolenic acid: 0.17Â±0.06g/100g). They concentrated in the phospholipids, characterised by a majority of choline-carrying species and high levels of lyso-phospholipids. These results confirm that Rhynchophorus phoenicis larvae are a potential alternative source of dietary fat and proteins that can be used to address under-nutrition and malnutrition.	Irrelevant	Title and abstract are not relevant with the topic	References removed
73	R C Hagan, R Villota	Effects of nonprotein substances on protein hydrolysis and plastein formation	1987	Food Chemistry	10.1016/0308-8146(87)90114-2	Effects of various naturally occurring nonprotein substances (carbohydrates, polysaccharides, fats and salts) on enzymatic hydrolysis of soy protein isolate and plastein formation from hydrolyzed soy protein were investigated. Relative extent of hydrolysis and plastein formation were measured as protein solubility in 10% trichloroacetic acid (TCA) since this method was found suitable for analysis of turbid, viscous and/or low protein samples. The presence of guar, xanthan, locust bean and arabic gums, arabinogalactan, unsaturated fatty acids (2%), salt mixture and xylan were found to enhance soy protein peptic hydrolysis at 0·5% enzyme/substrate; unsaturated fatty acids (1%) inhibited hydrolysis. At enzyme/substrate of 3·5%, hydrolysis was enhanced by xanthan gum, unsaturated fatty acids and sodium chloride but inhibited by gum karaya, salt mixture, starch, cellulose, and saturated fatty acids. Plastein synthesis was inhibited by xanthan, locust bean and guar gums but stimulated by arabinogalactan. Several nonprotein substances were found to interfere with the TCA solubility assay. Positive interference was noted for systems containing saturated and unsaturated fatty acids and magnesium, but negative interference was observed for systems containing guar gum, xanthan gum, calcium chloride and gum arabic.	Irrelevant	Title and abstract are not relevant with the topic	References removed
74	Andrea Osimani, Vesna MilanoviÄ‡, Federica Cardinali, Andrea Roncolini, Cristiana Garofalo, Francesca Clementi, Marina Pasquini, Massimo Mozzon, Roberta Foligni, Nadia Raffaelli, Federica Zamporlini, Lucia Aquilanti	Bread enriched with cricket powder (Acheta domesticus): A technological, microbiological and nutritional evaluation	2018	Innovative Food Science & Emerging Technologies	https://doi.org/10.1016/j.ifset.2018.06.007	Recently, the high nutritional value of edible insects attracted the attention of researchers and food industry for their potential use in foods with enhanced nutritional characteristics. In this study cricket (Acheta domesticus) powder was added to wheat flour to obtain bread with enhanced nutritional value. Bread loaves were obtained from doughs produced using different blends of wheat flour and cricket powder added in amounts of 10 or 30% (based on wheat flour) and baker's yeast and/or sourdough. Raw materials, doughs and breads were subjected to technological, microbiological, chemical and sensory analyses. Overall, a negative linear correlation between the amount of added cricket powder and the dough technological parameters was seen. However, compared to control breads produced with the sole wheat flour, breads containing cricket powder showed a higher nutritional profile in terms of fatty acid composition, high protein content and occurrence of essential amino acids. Finally, bread enriched with 10% cricket powder showed a discrete global liking by untrained panelists. Data overall collected highlighted a good suitability of cricket powder for the production of enriched bread. Of note, the presence of spore-forming bacteria in cricket-based bread loaves, thus highlighting potential safety issues to be deepened. Industrial relevance The present study demonstrated that edible insects powder can successfully be included in leavened baked goods to enhance their protein content. The present research also responds to the growing awareness of consumers towards innovative and wholesome leavened goods, proving that edible insects can constitute a novel source of innovative ingredients to be used in bread making.	Irrelevant	Title and abstract are not relevant with the topic	References removed

75	Aymar Rodrigue Fogang Mba, Germain Kansci, MichÃ¨le Viau, Rodolphe Rougerie, Claude Genot	Edible caterpillars of Imbrasia truncata and Imbrasia epimethea contain lipids and proteins of high potential for nutrition	2019	Journal of Food Composition and Analysis	https://doi.org/10.1016/j.jfca.2019.03.002	Imbrasia truncata and I. epimethea caterpillars were evaluated as dietary protein and lipid sources. They contained approximately 7.0â€‰g/100â€‰g fresh weight (FW) of lipids and 20.0â€‰g/100â€‰g FW of proteins calculed with determined nitrogen to protein conversion factors: 6.01â€‰Â±â€‰0.21 and 6.27â€‰Â±â€‰0.15 for I. truncata and I. epimethea, respectively. Unsaturated fatty acids represented about 2.63â€‰Â±â€‰0.21â€‰g/100â€‰g FW for I. truncata and 3.24â€‰Â±â€‰0.21â€‰g/100â€‰g FW for I. epimethea, with Î±-linolenic acid as major fatty acid (around 1.88â€‰Â±â€‰0.15â€‰g/100â€‰g FW for I. truncata 2.17â€‰Â±â€‰0.13â€‰g/100â€‰g FW for I. epimethea) and very low n-6/n-3 ratios: 0.15 (I. truncata) and 0.27 (I. epimethea). Polar lipids (phospholipids and glycolipidsâ€‰+â€‰sulfolipids), representing between 4 and 6% of lipids, contained little amounts of arachidonic acid (C20:4n-6). The major tocopherol isomer was Î±â ˆ’tocopherol in I. truncata (0.52â€‰Â±â€‰0.08â€‰g/100â€‰g FW) and Î³â ˆ’tocopherol in I. epimethea (1.00â€‰Â±â€‰0.08â€‰g/100â€‰g FW). The proteins of both insect included all indispensable amino acids at amounts (mg/g protein) higher than the indispensable amino acid requirement patterns recommended by WHO/FAO/UNU (2007). In conclusion, Imbrasia caterpillars exhibit a great nutritional potential due to the presence of good quality proteins and healthy fat	Irrelevant	Title and abstract are not relevant with the topic	References removed
76	Y Chen, J Zhao, W Zhang, T Zhao, Q Zhang, G Mao, W Feng, Q Li, L Yang, X Wu	Purification of novel polypeptides from bee pupae and their immunomodulatory activity in vivo and in vitro	2022	Journal of Insects as Food and Feed	10.3920/JIFF2021.0190	Edible insects have been proposed as an understudied food whose cultivation could be increased with global population growth. However, the bioactivators and bioactivities of bee pupae are poorly studied. In this paper, the active ingredients of bee pupa powder were analysed, and novel bee pupa polypeptides (BPP) were obtained through protein hydrolysis with alkaline protease. Two purified polypeptide components (BPP-21 and BPP-22) were isolated and purified on a diethylaminoethyl-sepharose fast flow column and a Sephadex G-25 column, and identified using size exclusion chromatography-high performance liquid chromatography and amino acid composition analyses. Due to its higher cell proliferation activity, BPP-22 was selected for further study of its immunomodulatory activity and mechanismin vivo andin vitro. The analysis of immunomodulatory activity showed that BPP-22 significantly increased the body weight growth rate, organ index, macrophage phagocytosis, delayed-type hypersensitivity reaction, cytokine level (interleukin (IL)-2 and interferon (IFN)-γ), immunoglobulin (Ig) levels (IgA, IgG, and IgM), and routine blood indexes in cyclophosphamide-treated immunosuppressed mice (P<0.01). Mechanistic research in RAW264.7 cells showed that BPP-22 might promote the secretion of cytokines (IL-2, tumour necrosis factor-α and IFN-γ) and the production of nitric oxide by increasing homologous mRNA expression and could exert immunomodulatory activity by increasing the phosphorylation of ERK and p38, and modulating the expression of intranuclear transcription factors (EIK-1, MEF-2 and CREB) in the MAPK signalling pathway. These findings are helpful for promoting the application of bee pupae as potential immunomodulatory agents and protein supplements.	Relevant	Title and abstract are relevant to the topic	References are used for the full review stage
77	Francesca Accardo, Alba MiguÃ©ns-GÃ³mez, Veronica Lolli, Andrea Faccini, Anna ArdÃ©vol, Ximena Terra, Augusta Caligiani, Montserrat Pinent, Stefano Sforza	Molecular composition of lipid and protein fraction of almond, beef and lesser mealworm after in vitro simulated gastrointestinal digestion and correlation with the hormone-stimulating properties of the digesta	2022	Food Research International	https://doi.org/10.1016/j.foodres.2022.111499	The current production of meat presents many disadvantages for the environment and much research focuses on alternative protein sources. Insects are novel protein sources highly valued for their nutritional and sustainable potential. However, many aspects concerning biological and nutritional properties of the insects after digestion, in comparison with other protein sources, are still overlooked. In this work, a comparative study on three different protein sources, namely almond, lean beef and insect Alphitobius diaperinus (lesser mealworm), was performed after in vitro simulated gastrointestinal digestion. An in-depth characterization of the chemical composition of the solubilized protein and lipid fractions of the digesta was performed by applying different analytical techniques, including chromatographic methods coupled to mass spectrometry and 1H NMR spectroscopy. Beef and insect were proven to be very similar in amino acid composition and protein solubilization after digestion, when considering the proper corrections for the chitin content. Lipid fraction from insects was solubilized during digestion as the one of almonds, but with a fastest kinetics. Thus, lesser mealworms are a good source of both lipids and highly nutritional proteins. Then, the amino acid composition of raw and digested protein fraction from the three sources was related to the PYY, ghrelin, GLP-1 and CCK release and ratsâ€™ food intake. The composition of amino acids in insect digesta was found to be related to specific effects on enterohormone release, and the modulation of food intake in rats.	Irrelevant	Title and abstract are not relevant with the topic	References removed

78	Dora Elisa Cruz-Casas, CristÃ³bal N Aguilar, Juan A Ascacio-ValdÃ©s, RaÃºl RodrÃguez-Herrera, MÃ³nica L ChÃ¡vez-GonzÃ¡lez, Adriana C Flores-Gallegos	Enzymatic hydrolysis and microbial fermentation: The most favorable biotechnological methods for the release of bioactive peptides	2021	Food Chemistry: Molecular Sciences	https://doi.org/10.1016/j.fochms.2021.100047	Bioactive peptides are biomolecules derived from proteins. They contain anywhere from 2 to 20 amino acids and have different bioactivities. For example, they have antihypertensive activity, antioxidant activity, antimicrobial activity, etc. However, bioactive peptides are encrypted and inactive in the parental protein, so it is necessary to release them to show their bioactivity. For this, there are different methods, where biotechnological methods are highly favorable, highlighting enzymatic hydrolysis and microbial fermentation. The choice of the method to be used depends on different factors, which is why it is essential to know about the process, its principle, and its advantages and disadvantages. The process of peptide release is critical to generate various peptide sequences, which will produce different biological effects in the hydrolysate. This review focuses on providing extensive information on the enzymatic method and microbial fermentation to facilitate selecting the method that provides the most benefits.	Irrelevant	Title and abstract are not relevant with the topic	References removed
79	Sayan Deb Dutta, Keya Ganguly, Min-Soo Jeong, Dinesh K Patel, Tejal V Patil, Seong-Jun Cho, Ki-Taek Lim	Bioengineered Lab-Grown Meat-like Constructs through 3D Bioprinting of Antioxidative Protein Hydrolysates.	2022	ACS applied materials & interfaces	10.1021/acsami.2c10620	Lab-grown bovine meat analogues are emerging alternatives to animal sacrifices for cultured meat production. The most challenging aspect of the production process is the rapid proliferation of cells and establishment of the desired 3D structure for mass production. In this study, we developed a direct ink writing-based 3D-bioprinted meat culture platform composed of 6% (w/v) alginate and 4% (w/v) gelatin (Alg/Gel)-based hydrogel scaffolds supplemented with naturally derived protein hydrolysates (PHs; 10%) from highly nutritive plants (soybean, pigeon pea, and wheat), and some selected edible insects (beetles, crickets, and mealworms) on in vitro proliferation of bovine myosatellite cells (bMSCs) extracted from fresh meat samples. The developed bioink exhibited excellent shear-thinning behavior (n < 1) and mechanical stability during 3D bioprinting. Commercial proteases (Alcalase, Neutrase, and Flavourzyme) were used for protein hydrolysis. The resulting hydrolysates exhibited lower-molecular-weight bands (12-50 kDa) than those of crude isolates (55-160 kDa), as determined by sodium dodecyl sulfate-polyacrylamide gel electrophoresis. The degree of hydrolysis was higher in the presence of Alcalase for both plant (34%) and insect (62%) PHs than other enzymes. The 3D-printed hydrogel scaffolds displayed excellent bioactivity and stability after 7 days of incubation. The developed prototype structure (pepperoni meat, 20 Ã— 20 Ã— 5 mm) provided a highly stable, nutritious, and mechanically strong structure that supported the rapid proliferation of myoblasts in a low-serum environment during the entire culture period. The 2,2-diphenyl-1-picrylhydrazyl radical scavenging assay enhanced the free radical reduction of Alcalase- and Neutrase-treated PHs. Furthermore, the bioprinted bMSCs displayed early myogenesis (desmin and Pax7) in the presence of PHs, suggesting its role in bMSC differentiation. In conclusion, we developed a 3D bioprinted and bioactive meat culture platform using Alg/Gel/PHs as a printable and edible component for the mass production of cultured meat.	Irrelevant	Title and abstract are not relevant with the topic	References removed
80	A Enta, M Hayashi, M L Lopez Caceres, L Fujiyoshi, T Yamanaka, A Oikawa, F Seidel	Nitrogen resorption and fractionation during leaf senescence in typical tree species in Japan	2020	Journal of Forestry Research	10.1007/s11676-019-01055-z	In northeastern Japan, an area of high precipitation and mountains, beech (Fagus creanata Blume), larch (Larix kaempferi Lamb.), cedar (Cryptomeria japonica D. Don) and black locust (Robinia pseudoacacia L.) were evaluated for N resorption and N isotope fractionation in pre- and post-abscission leaves in comparison to green leaves. The highest leaf N concentration in summer corresponded to the N-fixing black locust, followed in decreasing order by the deciduous beech and larch and evergreen cedar. On the other hand, the lowest N resorption efficiency corresponded to black locust and the highest to beech, in increasing order by larch and cedar. All tree species returned significant amounts of N before leaf abscission; however, N isotope fractionation during leaf N resorption was only found for beech, with a depleted N isotope value from green to pre-abscission leaf. The most N, however, was resorbed from pre-abscission to post-abscission. This result may indicate that δ15N fractionation took place during N transformation processes, such as protein hydrolysis, when the concentration of free amino acids increased sharply. The difference in the type of amino acid produced by each species could have influenced the N isotope ratio in beech but not in the other tree species. The results of this study showed that it is possible to infer the type and timing of processes relevant to N resorption by analyzing leaf δ15N variation during senescence.	Irrelevant	Title and abstract are not relevant with the topic	References removed

81	Forough Jahandideh, Stephane L Bourque, Jianping Wu	A comprehensive review on the glucoregulatory properties of food-derived bioactive peptides	2022	Food Chemistry: X	https://doi.org/10.1016/j.fochx.2022.100222	Diabetes mellitus, a group of metabolic disorders characterized by persistent hyperglycemia, affects millions of people worldwide and is on the rise. Dietary proteins, from a wide range of food sources, are rich in bioactive peptides with antidiabetic properties. Notable examples include AGFAGDDAPR, a black tea-derived peptide, VRIRLLQRFNKRS, a Î²-conglycinin-derived peptide, and milk-derived peptide VPP, which have shown antidiabetic effects in diabetic rodent models through variety of pathways including improving beta-cells function, suppression of alpha-cells proliferation, inhibiting food intake, increasing portal cholecystokinin concentration, enhancing insulin signaling and glucose uptake, and ameliorating adipose tissue inflammation. Despite the immense research on glucoregulatory properties of bioactive peptides, incorporation of these bioactive peptides in functional foods or nutraceuticals is widely limited due to the existence of several challenges in the field of peptide research and commercialization. Ongoing research in this field, however, is fundamental to pave the road for this purpose.	Irrelevant	Title and abstract are not relevant with the topic	References removed
82	Su-Hyeon Pyo, Chae-Ryun Moon, So-Won Park, Ji-yu Choi, Jong-Dae Park, Jung Min Sung, Eun-Ji Choi, Yang-Ju Son	Quality and staling characteristics of white bread fortified with lysozyme-hydrolyzed mealworm powder (Tenebrio molitor L.)	2024	Current Research in Food Science	https://doi.org/10.1016/j.crfs.2024.100685	Edible insects have a low environmental impact but are rich in nutrients and have been promoted as alternative protein sources. However, adding insect flour to bread negatively affects the overall quality, especially loaf volume and textural properties. Furthermore, relevant studies on chitin are limited. Therefore, this study examined chitin hydrolysis using lysozymes to enhance the quality characteristics in defatted mealworm (Tenebrio molitor L.) powder (DF-M)-supplemented bread. The chitin hydrolysis degree by lysozymes was evaluated using the 3,5-dinitrosalicylic acid assay and matrix-assisted laser desorption ionizationâ€“time-of-flight mass spectrometry. The amount of chitin oligomers increased with time, and no significant difference in the hydrolysis efficiency between water and 400Â mM acetate buffer was observed. Enzymatic hydrolysis improved the DF-M water- and oil-binding and antioxidant capacities. In addition, chitin hydrolysis increased the volume and softened the texture of white bread. In particular, bread supplemented with DF-M hydrolyzed for 4Â h at 10 % had the highest moisture content among the mealworm-added bread groups during storage for 5 days. Moreover, sensory evaluation showed a positive effect of chitin hydrolysis on acceptability. Our findings indicate that chitin hydrolysis can improve the quality of bread containing insect additives. In conclusion, this study provides novel insights into producing high-quality and functional bakery products from edible insects by the enzymatic hydrolysis of edible insect powders and could expand the applications of edible insects as food ingredients.	Relevant	Title and abstract are relevant to the topic	References are used for the full review stage
83	Shubam Singh, Hina F Bhat, Sunil Kumar, Mehnaza Manzoor, Aunzar B Lone, Pawan Kumar Verma, Rana Muhammad Aadil, Konstadina Papastavropoulou, Charalampos Proestos, Zuhaib F Bhat	Locust protein hydrolysates have the potential to enhance the storage stability of cheese	2023	Current Research in Food Science	https://doi.org/10.1016/j.crfs.2023.100561	The study evaluated the efficacy of locust protein hydrolysates (LoPHs) to enhance the quality of Cheddar cheese (ChCh) during storage. The locust protein (LoP) was pre-treated [microwave (Mic) or ultrasonication (Ult) or no treatment (Not)] before hydrolysis using alcalase enzyme (3% w/w). The ChCh samples containing LoPHs at the maximum level of 1.5% were evaluated for quality for 3 months (4Â Â±Â 1Â Â°C) and subjected to gastrointestinal simulation. Both pre-treatments (Mic and Ult) significantly (PÂ <Â 0.05) enhanced the antimicrobial and antioxidant activities of the LoPHs (UltÂ >Â MicÂ >Â Not). The ChCh samples with LoPHs exhibited significantly (PÂ <Â 0.05) lower means for lipid oxidation (TBARS and free fatty acids), protein oxidation (total-carbonyl content) and microbial counts (psychrophilic, total plate and yeast/moulds) during the storage. A positive effect was found on the sensory quality of ChCh samples after one month of storage. The gastrointestinal simulation improved the antioxidant capacity of the stored ChCh samples. LoPHs can be used as a novel bio-preservative for cheese.	Relevant	Title and abstract are relevant to the topic	References are used for the full review stage

84	Tae-Kyung Kim, Hyun-Jung Yun, Yea-Ji Kim, Ji Yoon Cha, Yun-Sang Choi	Allomyrina dichotoma larvae extract as a novel tenderizer on brined pork loin and changes in quality based on extraction methods	2025	Applied Food Research	https://doi.org/10.1016/j.afres.2024.100650	This study investigated the effects of Allomyrina dichotoma larvae extract, a novel food resource, on the tenderization and quality enhancement of brined pork loin. The extraction buffers used were distilled water and 0.58â€¯M saline buffer, and the extracted insect solution and residue were added to the brine. Treatments with insect extracts showed improved tenderization, as evidenced by lower shear force values, enhanced water-holding capacity, and reduced lipid oxidation, suggesting the potential for longer shelf life and improved nutritional quality. These findings provide valuable insights into the use of edible insect extracts in meat processing, highlighting the benefits of this innovative approach for enhancing meat quality for various consumer segments, including older adults. Overall, this study underscores the potential of edible insects as sustainable and effective ingredients in meat-curing processes, offering a promising avenue for the development of more nutritious and palatable meat products.	Irrelevant	Title and abstract are not relevant with the topic	References removed
85	Lawrence I Gilbert	Lipid Metabolism and Function in Insects	1967		https://doi.org/10.1016/S0065-2806(08)60208-8	Publisher Summary One of the fundamental questions concerning the role of lipids in the physiology of the insect concerns the quantity of lipid contained upon, and interior to, the rigid exoskeleton. Lipids are of vital importance to many insects as substrates for embryogenesis, metamorphosis and flight. Although several problems of the function and metabolism of lipid in insects have been unraveled in recent years, this research area remains ripe for invasion by both the entomologist and biochemist. The task of assuring that insects utilize the same metabolic pathways as micro-organisms and vertebrates has not yet been completed. This chapter concentrates on developments within the past ten years but also discusses older literature when applicable to the historical development of the topic. In most cases, sophisticated experiments have been conducted on only a few species of â€œdomesticatedâ€insects and the result cannot be extrapolated to all insects. The multitude of different ecological niches and behavioral characteristics are no doubt reflected in a great number of metabolic variations on perhaps more than one basic theme.	Irrelevant	Title and abstract are not relevant with the topic	References removed
86	A K Charnley, J Hunt, R J Dillon	The germ-free culture of desert locusts, Schistocerca gregaria	1985	Journal of Insect Physiology	https://doi.org/10.1016/0022-1910(85)90096-4	A technique is described for rearing germ-free desert locusts. Axenic insects appeared to develop and reproduce normally, therefore it is concluded that the gut bacterial flora does not contribute significantly to locust nutrition. However, it is suggested that the gut flora may benefit its host by contributing to the locust's defences against attack by pathogenic microorganisms. The results of morphometric measurements are discussed in terms of the bacterial origin of the so called phase pheromone, locustol. No evidence was found to support the hypothesis that microbial activity restricts gut carbohydrase.	Irrelevant	Title and abstract are not relevant with the topic	References removed
87	JosÃ© L Soulages, Michael A Wells	Lipophorin: The Structure of an Insect Lipoprotein and Its Role in Lipid Transport in Insects	1994	Lipoproteins, Apolipoproteins, and Lipases	https://doi.org/10.1016/S0065-3233(08)60644-0	Publisher Summary A significant way of organizing a lipid transport system is provided by insect lipoproteins, which provide an interesting contrast to the mammalian lipid transport system. In insects, a single lipoprotein, lipophorin, acts as a reusable shuttle to transfer diglycerides between the fat bodies, where they are stored as triglycerides, and the peripheral tissues. This chapter describes the composition and the organization of lipids and proteins in the lipophorins and the structure of apolipophorin-III that is determined by X-ray crystallography to 2.5 Ã…. Lipophorin is reloaded with lipid by another remarkable protein, the lipid transfer particle, which can affect a net transfer of diglycerides from the fat bodies to the depleted lipophorin particles. The major progress that is achieved in describing the lipid transport system in insects provides the results of significance for understanding lipid transport in general, an illustration of comparative biochemistry at its best. The only common major lipid component of vertebrate and insect lipoproteins is phospholipid (PL). The physiological roles of lipophorin in the biosynthesis and metabolism are also discussed.	Irrelevant	Title and abstract are not relevant with the topic	References removed

88	James T Bradley, Barbara H Estridge, Malgorzata Kloc, Karen G Wolfe, Szczepan M Bilinski	Balbiani bodies in cricket oocytes: Development, ultrastructure, and presence of localized RNAs	2001	Differentiation	https://doi.org/10.1046/j.1432-0436.2001.670404.x	Formation of two spherical Balbiani bodies along the long axis of previtellogenic oocytes in Acheta domesticus was demonstrated by differential interference microscopy. The structures form adjacent to and on opposite sides of the germinal vesicle, the anterior body first. Each migrates to the nearest pole of the elongating oocyte and retains its spherical structure until occluded from view by accumulating yolk. In situ hybridization, immunocytochemistry, and confocal immunofluorescent microscopy showed Balbiani body components to include Î³-tubulin, Î±-tubulin, EF1Î±, and several RNAs homologous to localized Xenopus RNAs implicated in embryonic axis formation or germ cell determination. The latter include Xcat2, Xwnt11, Xlsirt, and Xpat. Balbiani body ultrastructure includes a dense cloud of tubular mitochondria, rough ER, Golgi-like membrane aggregates, and microtubules. The results suggest that molecules and mechanisms specifying early determinative events for embryogenesis in vertebrates and insects are highly conserved and that Balbiani bodies may have a role in establishing developmental asymmetry in the cricket.	Irrelevant	Title and abstract are not relevant with the topic	References removed
89	Sandro R Marana, Alberto F Ribeiro, Walter R Terra, ClÃ©lia Ferreira	Ultrastructure and secretory activity of Abracris flavolineata (Orthoptera: Acrididae) midguts	1997	Journal of Insect Physiology	https://doi.org/10.1016/S0022-1910(96)00117-5	The midgut of Abracris flavolineata adults comprises a ventriculus and six anteriorly placed caeca each displaying an anterior and a posterior lobe. Columnar cells in the caeca and anterior ventriculus present secretory vesicles originating from abundant Golgi areas, which seem to result (through exocytosis) in dark granules among the microvilli. A. flavolineata males were starved for 24 h, fed for 20 min at noon and dissected at 0, 1, 3 and 5 h after the meal. Enzyme assays were accomplished on crop and caecal contents and in subcellular fractions obtained from the isolated anterior caeca. Subcellular fractions putatively containing secretory vesicles were recognized. Digestive enzyme activity is usually low (amylase is high) in the secretory vesicles in starving insects, decreases 1 h after the meal, increases at 3 h, and thereafter decreases again (amylase remains constant). In caecal contents, digestive enzymes decrease at 1 h and increase at 3 h after the meal, the contrary being true for crop contents. Thus, in A. flavolineata caecal cells, digestive enzymes (Î²-glucosidase is an exception) are synthesized and secreted by exocytosis in response to feeding. Also in response to feeding, digestive enzymes are transferred from caecal contents to the crop and, after about 3 h following the meal, crop-caecal dispersed material with accompanying enzymes are translocated to the caeca, where digestion ends and absorption occurs.	Irrelevant	Title and abstract are not relevant with the topic	References removed
90	R H Dadd	Feeding Behaviour and Nutrition in Grasshoppers and Locusts	1963		https://doi.org/10.1016/S0065-2806(08)60174-5	Publisher Summary This chapter relates feeding behavior and nutrition to the other aspects of insect functioning that are sufficiently well surveyed and understood to provide the possibility of integration. The food preferences of some acridids are strictly limited and a degree of discrimination is usually to be discerned even in those of polyphagous propensities. Many of these latter require little pressure to feed outside their range of native preference and this may lead to their becoming pests. The indications are that for species of such catholic tastes nutritional factors enter into their choice of food. Nutrition in the strict chemical sense of certain locusts, their needs, while qualitatively similar to those of the generality of insects, is noteworthy for a few unusual features. These relate to the requirement for ascorbic acid, inositol, and unsaturated fatty acids and to the critical importance of large amounts of choline in larval growth. A need for the lipogenic factors, choline and inositol, and unsaturated fatty acids seems to characterize all those orthopteroid insects that have been studied in detail. The need for dietary carotene to ensure normal pigment metabolism in locusts may be a peculiarity of those particular species in which pigmentation is bound up with the special physiological phenomena of phase.	Irrelevant	Title and abstract are not relevant with the topic	References removed
91	Julian A T Dow	Insect Midgut Function	1987		https://doi.org/10.1016/S0065-2806(08)60102-2	Publisher Summary This chapter presents recent progress in the study of the midgut, particularly with reference to ion and water transport. However, the midgut does not function in isolation, and so the attempt is to fit the role of the midgut into the context of the operation of the gut as a whole. The chapter considers the cockroach as an example of a classic generalist and looks at scavenging dipteran larvae, mainly aquatic. Midgut function is described in various types of insects such as solid/plant feeders (phytophagous insects), solid/animal feeders (carnivores), liquid/animal feeders (bloodsuckers), and liquid/plant feeders (sap and nectar feeders). The chapter also discusses the process of digestion of cellulose in insects. There are four categories of evidence that an insect can digest cellulose. It could survive on a diet of pure synthetic cellulose; it could be shown to incorporate and metabolize label from radioactive cellulose in the food; it could be shown to break down synthetic crystalline cellulose; or it could be shown that there is less cellulose in the excreta than was consumed in the food.	Irrelevant	Title and abstract are not relevant with the topic	References removed

92	C F Hinks, M A Erlandson	The accumulation of haemolymph proteins and activity of digestive proteinases of grasshoppers (Melanoplus sanguinipes) fed wheat, oats or kochia	1995	Journal of Insect Physiology	https://doi.org/10.1016/0022-1910(94)00116-X	Lesser migratory grasshoppers, Melanoplus sanguinipes (Fab.), were fed single plant diets of wheat, oats or kochia foliage in the instar preceding sampling, from the 2nd to 5th instars. Haemolymph proteins, tryptic and chymotryptic activity in the gut and total gut protein were determined. Nutritional indices were calculated from data collected from 2nd-instar grasshoppers. In this instar, grasshoppers consumed more than twice as much kochia as either wheat or oats, and produced correspondingly greater amounts of frass. The duration of the instar was in the order wheat < kochia < oats. Approximate digestibility was significantly lower in grasshoppers fed oats than those fed either wheat or kochia. The efficiency of conversion of digested food was significantly lower and the relative consumption rate significantly higher in those fed kochia than either wheat or oats. The efficiency of conversion of ingested food was in the order wheat > oats > kochia. Haemolymph concentrations of both putative apoprotein I and the comigrating complex of apoprotein II and larval storage protein (LSP) subunits (70â€"80 kDa mol. wt) were significantly higher in grasshoppers fed the standard colony diet of wheat germ, lettuce and wheat in all instars than for those grasshoppers fed single-plant diets. Among grasshoppers fed the single-plant diets, those fed wheat had the highest levels of putative apoprotein/LSP. On an individual grasshopper basis, there was no correlation between the concentrations of apoprotein/LSP in the haemolymph and the levels of gut protein, trypsin or chymotrypsin. Total protein in whole-gut homogenates was significantly higher in kochia-fed grasshoppers in the 2nd instar and significantly lower in those fed oats in the 5th instar. In all instars, grasshoppers fed oats had higher total trypsin activity than those fed wheat; ratios of trypsin activity in oat- vs wheat-fed grasshoppers were 1.8 : 1, 1.9 : 1, 3.8 : 1 and 1.9 : 1 for the 2nd, 3rd, 4th and 5th instars, respectively. Total chymotrypsin activity was significantly higher in grasshoppers fed oats in the 4th and 5th instars, and in those fed kochia in the 5th instar. The ratios of chymotrypsin activity in oat- vs wheat-fed grasshoppers were 1.5 : 1, 1.0 : 1, 2.5 : 1 and 1.9 : 1 for the 2nd, 3rd, 4th and 5th instars, respectively. The present study confirmed that both the kochia and oats diets were inferior in supporting grasshopper growth, probably because of lower protein acquisition and hypermodulation of digestive proteinases. However, the levels of activity of these enzymes were considerably lower in the earlier instars than those previously reported in adults of this species. The results are discussed in the context of hormonal activity and suggest that protein is allocated differently in nymphs compared to adults. Comparison of the physiological and biochemical responses of grasshoppers to wheat and oats and their variably resistant cultivars provides a valuable model for studying such interactions.	Irrelevant	Title and abstract are not relevant with the topic	References removed
93	R E Blackith	The water reserves of hatchling locusts	1961	Comparative Biochemistry and Physiology	https://doi.org/10.1016/0010-406X(61)90136-0	The demand for water reserves and conservation which various environments make on the locust species hatching into them is reflected by the greater efficacy of the waterproofing of the Desert locust as compared with the Red locust, which hatches into swampy grassland. This difference persists even when the water-proofing layer of the cuticle is disrupted, and abrasion is probably important to locust hatchlings exposed to blown sand or dust; the vermiform sheath protects them during the ascent through the soil, after eclosion. Water conservation is enhanced by the lysis of proteins to augment the osmotic pressure of the blood of starving, dehydrated hatchlings. This process is rapid in hatchlings whose cuticle has been abraded, and the behavioural changes which accompany dehydration in Catantopids will also tend to conserve water.Generally, the survival of hatchlings in dry atmosphere is greatest for those which weigh most at eclosion, and most of such differences of weight represent variation in the water reserves.	Irrelevant	Title and abstract are not relevant with the topic	References removed
94	L H Teo, A M Hammond, J P Woodring, H W Fescemyer	Study on some aspects of glycosidases present in the velvetbean caterpillar, Anticarsia gemmatii	1990	Comparative Biochemistry and Physiology Part B: Comparative Biochemistry	https://doi.org/10.1016/0305-0491(90)90212-C	The optimal pHs of the following enzymes were determined: sucrase, 6.4; maltase, 5.4–7.6; trehalase, 6.0; salicin hydrolase, 6.4. The optimal temperatures of sucrase, maltase and cellobiase were found to be 40°C and those of trehalase and salicin hydrolase were above 45°C. The highest activation energy was obtained with sucrase, followed by cellobiase, trehalase, salicin hydrolase and maltase. In all glycosidases studied, there were many more enzymes in the lumen than in the tissues of the gut.	Irrelevant	Title and abstract are not relevant with the topic	References removed

95	Georges PÃ©tavy	Contribution of the vitellophags to yolk digestion and cytophagocytosis during embryogenesis of the migratory locust, locusta migratoria L. (orthoptera : acrididae)	1986	International Journal of Insect Morphology and Embryology	https://doi.org/10.1016/0020-7322(86)90029-2	The vitellophags perform 2 essential functions during embryogenesis of the migratory locust, Locusta migratoria (Orthoptera : Acrididae). They contribute to yolk digestion and probably to translocation of the released nutrients toward the embryo. When the germ band, the amnion, and the serosa are segregated, the vitellophags become far more efficient than the various cells bordering the yolk. Their activity increases as the yolk cleavage progresses. The 2 kinds of yolk inclusions are modified differently. (i) The lipoglycoprotein bodies become altered very early through erosion, followed by fragmentation. The vitellophagic lysosomes increase in number, while the matrix of certain adjacent yolk bodies reacts to a test for acid phosphatases. (ii) The lipid droplets are modified in such a manner that more and more vitellophags become laden with tiny lipid globules. Moreover, several vitellophags phagocytize cells that die during normal embryogenesis. (i) Some of them ingest prematurely dead cells that are pushed aside from the germ anlage, then from the inner layer of germ band, toward the adjacent yolk. (ii) Before katatrepsis, a few vitellophags phagocytize some of the amniotic cells that degenerate ventrally to head and thorax. (iii) During and after the completion of dorsal closure, numerous vitellophags engulf and digest most of the extraembryonic cells that have been carried into the yolk mass.	Irrelevant	Title and abstract are not relevant with the topic	References removed
96	C F Hinks, M T Cheeseman, M A Erlandson, O Olfert, N D Westcott	The effects of kochia, wheat and oats on digestive proteinases and the protein economy of adult grasshoppers, Melanoplus sanguinipes	1991	Journal of Insect Physiology	https://doi.org/10.1016/0022-1910(91)90051-Z	Adult male grasshoppers (Melanoplus sanguinipes), fed diets of wheat, oats or kochia ad libitum varied in the amount of food consumed wheat > oats > kochia which paralleled weight gains but not the amount of frass produced. Approximate digestibility was significantly lower, and the efficiency of conversion of assimilated food was significantly higher on kochia than either of the cereals. Assays of proteolytic digestive enzymes on the three diets revealed that adults produced substantially more trypsin and chymotrypsin on oats and kochia than on wheat. Production of greater amounts of these enzymes occurred within seven days after introduction to oats or kochia. Electropherograms of haemolymph from grasshoppers fed oats or kochia had much less stainable protein compared to those fed wheat. All putative bands of vitellogenin from either haemolymph or fat body were substantially reduced on diets of oats or kochia. In the male reproductive system, the major accessory gland protein, long hyaline gland protein, was significantly reduced in kochia-fed grasshoppers. Leaves of wheat had significantly more precipitable protein than oats, and oats had significantly more precipitable protein than kochia. Measurable protein in the three diets may not completely account for the disparities in vitellogenins and other haemolymph proteins. It is suggested that the overproduction of digestive proteolytic enzymes may have been a contributory factor in protein attrition, resulting in slower growth and reduced reproduction. These observations explain in part the consequences of these diets to the biotic potential of grasshoppers.	Irrelevant	Title and abstract are not relevant with the topic	References removed

97	C F Hinks, D Hupka	The effects of feeding leaf sap from oats and wheat, with and without soybean trypsin inhibitor, on feeding behaviour and digestive physiology of adult males of Melanoplus sanguinipes	1995	Journal of Insect Physiology	https://doi.org/10.1016/0022-1910(95)00042-S	Newly-fledged adult male grasshoppers (Melanoplus sanguinipes Fab.) were fed measured quantities of freshly prepared sap from the leaves of seedlings of wheat (Triticum aestivum L. cv. Katepwa) or oats (Avenu sativa L. cv. Harmon), or sap from each plant to which soybean trypsin inhibitor (STI) was added. Different patterns of feeding with respect to movement and position of the antennae and palpi were recorded on oat sap compared to wheat sap. All grasshoppers were allowed to feed ad Lib twice daily on each diet and the volumes of sap imbibed were recorded. The volumes of sap imbibed were not significantly different during the first day of feeding, but on subsequent days they were significantly smaller in grasshoppers fed oat sap compared to those fed wheat sap. The addition of STI to oat sap had no effect on the volume imbibed. Meal volumes were more or less consistent from day to day in grasshoppers fed wheat sap, but the addition of STI to wheat sap stimulated a significant increase in the volume imbibed. After 5 days of feeding in this manner the grasshoppers were weighed, stimulated to regurgitate the crop contents, then dissected. The complete alimentary tract was removed from each grasshopper and assayed for trypsin and chymotrypsin activity. A significantly higher gain in fresh body weight was recorded in grasshoppers fed wheat sap + STI than in those fed wheat sap alone. In contrast, grasshoppers fed oat sap lost weight during the course of the experiment, and a greater loss was recorded from those fed oat sap to which STI was added. Protein concentration in the regurgitate varied significantly with diet, and was in the following order: wheat sap + STI > wheat sap > oat sap + STI = oat sap. Trypsin and chymotrypsin activities were in the same order as protein concentration. Small increases in trypsin activity were observed in the regurgitate from grasshoppers fed sap + STI from either plant, compared to the sap alone. A 20-fold increase in chymotrypsin activity was observed in the regurgitate of grasshoppers fed wheat sap + STI compared to the regurgitate in those fed wheat sap. A 4.5-fold increase in chymotrypsin activity was observed in the regurgitate of grasshoppers fed oat sap + STI compared to the regurgitate of those fed oat sap alone. Protein concentration in the gut tissues varied significantly with diet and paralleled those observed in the regurgitate. Small non-significant increases in trypsin activity were observed in the grasshoppers fed sap + STI from either plant, compared to the sap alone. A 6-fold increase in chymotrypsin activity was observed in the residual gut tissues from grasshoppers fed wheat sap + STI compared to wheat sap, but activity of this enzyme was lower in grasshoppers fed oat sap + STI compared to oat sap alone.The results are discussed in the context of protein availability and its allocation, and the dynamic interaction between antifeedants, phagostimulants and antibiosis factors and their influence on the impact of proteinase inhibitors.	Irrelevant	Title and abstract are not relevant with the topic	References removed
98	Donald W Roberts, Raymond J B T - Advances in Applied Microbiology St. Leger	Metarhizium spp., Cosmopolitan Insect-Pathogenic Fungi: Mycological Aspects	2004		https://doi.org/10.1016/S0065-2164(04)54001-7	Publisher Summary The chapter focuses on mycological aspects of the genus Metarhizium. The genus includes several species, varieties within species, and individual isolates with broad ranges of physiological traitsâ€"including host range. The interactions of these fungi with their hosts, and the large literature on their use for pest control, largely define the scientific and popular concepts of Metarhizium spp. Fungi of the hyphomycete genus Metarhizium have been isolated from infected insects and soil. Although some isolates of these fungi have rather restricted host ranges, the group is better known for its ability to kill a wide spectrum of insects, including insects in at least seven orders. The common name for Metarhizium-induced disease is â€œgreen muscardine,â€ based on the encrustation of insect cadavers with green conidia. The rapid increase in research on Metarhizium, followed by sustained high scientific output, can be explained by several important worldwide attitude changes and the initiation of several promising Metarhizium-based pest-control and molecular-biology efforts.	Irrelevant	Title and abstract are not relevant with the topic	References removed

99	ClÃ©lia Ferreira, Mariana C Oliveira, Walter R Terra	Compartmentalization of the digestive process in Abracris flavolineata (Orthoptera: Acrididae) adults	1990	Insect Biochemistry	https://doi.org/10.1016/0020-1790(90)90044-U	Carbohydrases predominate in the crop and their pH optima agree with pH prevailing in crop contents. Major amounts are also found in caecal contents. Aminopeptidase and trypsin are active mainly in the caeca, where they predominate in cells and contents, respectively. Aminopeptidase is partly membrane-bound. Except for trehalase, salivary glands display negligible amounts of digestive enzymes. The specific activity of digestive enzymes is high in all midgut cells and the enzyme molecules do not differ among gut compartments, as judged by polyacrylamide gel electrophoresis. Thus, it is probable that digestive enzymes are synthesized and secreted by all midgut cells (mainly in caeca) and then passed forward into the crop. Digestive enzymes are found in hindgut in concentrations similar to those in ventricular contents and, since they are stable in gut contents, they are likely excreted at a rate similar to undigested food. The data support the hypothesis that carbohydrate and protein are digested mainly in crop and caecal lumina, respectively, with part of the final digestion of proteins occurring at the surface of caecal cells. The peculiar features of the digestion of A. flavolineata grasshoppers, including the lack of midgut countercurrent fluxes, are thought to be derived from putative Polyneoptera ancestors.	Irrelevant	Title and abstract are not relevant with the topic	References removed
100	Amer I Tawfik, Anna VedrovÃ¡, Weiwei Li, FrantiÅ¡ek Sehnal, D Obeng-Ofori	Haemolymph ecdysteroids and the prothoracic glands in the solitary and gregarious adults of Schistocerca gregaria	1997	Journal of Insect Physiology	https://doi.org/10.1016/S0022-1910(96)00116-3	The haemolymph titre of ecdysteroids rises in the adults of both sexes to a brief peak in the first week after emergence. A major rise begins on days 8â€“10, coincidentally with intensive vitellogenesis in the females and aggregation pheromone production in the males. The titre reaches about 400 ng of 20-hydroxyecdysone (20E) equiv./ml in the solitary, but less than 150 ng/ml in the gregarious adults of both sexes. In the females, the titre drops after oviposition (prior to day 20) and remains low until the next egg batch matures; then it increases in either phase to 150â€“180 ng/ml. A similar titre reduction in the males occurs just after day 20: in the solitary males the titre declines slowly to about 110 ng/ml on day 40, whereas in the gregarious males ecdysteroids virtually disappear by day 25. The blood of females contains similar levels of 20E and ecdysone, whereas in the males 20E makes up 73% (solitary) or 94% (gregarious) of total ecdysteroids. Minor components include a compound behaving as makisterone A and highly polar metabolites. The prothoracic glands are well preserved in both solitary and gregarious adults for at least 1 week after fledging and in some females they seem to persist for the lifetime, but in contrast to the glands of larvae, do not produce significant amounts of ecdysteroids when cultured in vitro.	Irrelevant	Title and abstract are not relevant with the topic	References removed
101	Gerard R Wyatt, Kenneth G Davey	Cellular and Molecular Actions of Juvenile Hormone. II. Roles of Juvenile Hormone in Adult Insects	1996		https://doi.org/10.1016/S0065-2806(08)60030-2	Publisher Summary Among animal hormones, juvenile hormone (JH) is distinctive because of its unique structure and the diversity of its effects on insect development and reproduction. This chapter reviews the actions of JH on the fat body, gonads, accessory glands, muscle, and nervous system of adult insects. Whereas the epidermis is a major target of premetamorphic JH action, it has been studied little in adult insects, which generally do not moult. However, since the development of yellow pigmentation that accompanies sexual maturation in adult male locusts is clearly dependent on JH-regulation processes, in which cellular and molecular mechanism are investigated. The rapid recent progress in understanding how ecdysteroids regulate the gene activities has resulted from the opportunities afforded by drosophila melanogaster: mapped and characterized mutants, polytene chromosomes with puffs marking active genes, efficient germ-line transformation. The recognition of two aspects of JH action in the tissues of adult insects is reviewed. A model for understanding some aspects of priming by JH may be found in the action of ecdysteroids, where early genes produce factors needed for the expression of late genes. In structure, thyroxine is very different from JH, but there is considerable resemblance between thyroxine and phenoxyphenoxy carbamate, fenoxycarb. Functionally, there are marked similarities. Thyroxine governs metamorphosis in amphibians, but is remarkably pleiotropic in governing many processes ranging from the maturation of the central nervous system to thermoregulation. The chapter emphasizes the importance of selecting insect systems on the basis of their optimal features for research, rather than historical precedent or economic importance. With selection of appropriate systems and application of the cell and molecular research techniques now available, the elusive problem of JH action should soon yield to enlightenment.	Irrelevant	Title and abstract are not relevant with the topic	References removed

102	Alan D Elbein	The Metabolism of Î±,Î±-Trehalose**The work cited from the author's laboratory was supported by grants from the Robert A. Welch Foundation and the National Institutes of Health (AI 09402).	1974		https://doi.org/10.1016/S0065-2318(08)60266-8	Publisher Summary This chapter discusses the metabolism of Î±,Î±-trehalose. The chapter outlines the various reactions that have been shown to be involved in the metabolism of trehalose. The other isomers of trehalose containing D-glucopyranoseâ€"that is, Î±,Î²-trehalose and Î²,Î²-trehalose have been synthesized chemically. However, except for a few rare cases, these isomers of trehalose do not appear to be naturally occurring. The mechanism of biosynthesis of Î±,Î±-trehalose. To determine the role of trehalose in Mycobacterium the levels of free and â€œboundâ€(that is, lipid-associated) trehalose and glycogen during growth of Mycobacterium smegmutis under various conditions of nitrogen limitation is examined. Whereas the glycogen levels increased markedly as the nitrogen content of the medium was lowered, the levels of trehalose remained fairly constant. The results of labeling studies suggested that the free trehalose in these cells may be utilized for purposes except as an energy reserve, whereas glycogen is probably stored mainly as a reserve. Synthesis and degradation of trehalose constitute a mechanism for the resorption of D-glucose in the kidney and, perhaps, also in the intestine. It has also been suggested that trehaloseâ€"like another naturally occurring, nonreducing disaccharide, sucroseâ€"could function in the movement of carbohydrate, .i.e., as a translocate in plants or insects, or both.	Irrelevant	Title and abstract are not relevant with the topic	References removed
103	G R Wyatt	The Biochemistry of Sugars and Polysaccharides in Insects	1967		https://doi.org/10.1016/S0065-2806(08)60210-6	Publisher Summary In their carbohydrate metabolism, insects make use of substances, enzymes and operational principles that are known from other living groups. Occupying a central place among the knowledge of carbohydrate metabolism in insects is the presence of trehalose as the predominant blood sugar. Its distribution and the relations of its metabolism to the mechanisms of nutrient absorption, storage and supply to the tissues, are discussed. Characteristic of insects and other arthropods is the presence of chitin as a structural component of the exoskeleton, which in its deposition and resorption interacts closely with the metabolism of other carbohydrates. The mucopolysaccharides of insect hemolymph and tissues have only recently begun to be investigated. This chapter focuses on a metabolic process, which apparently peculiar to insects and some other arthropods is the bulk conversion of glycogen to glycerol and sorbitol. Insects have provided the material for several well-known original biochemical discoveries. The chapter also presents an investigation of the biochemistry of this animal group, which has in general lagged far behind that of micro-organisms on the one hand and vertebrate animals on the other.	Irrelevant	Title and abstract are not relevant with the topic	References removed
104	J D Thomas	Schistosomiasis and the Control of Molluscan Hosts of Human Schistosomes with Particular Reference to Possible Self-regulatory Mechanisms	1973		https://doi.org/10.1016/S0065-308X(08)60189-X	Publisher Summary This chapter discusses the mechanisms involved in controlling the population growth of Biomphalaria glabrata (Say), one of the molluscan hosts of Schistosoma mansoni Sambon. It is hoped that the information thus gained can be utilized in devising means of controlling the population growth of the snail hosts and thus preventing transmission of the schistosome parasites to the mammalian host. The various strategies that might succeed in preventing transmission of schistosomes, including the control of snail hosts, and reducing the probability of success of miracidia, sporocysts, cercariae, and adult parasites are discussed and evaluated. The chapter discusses the results of experiments designed to show how various environmental factors including contact by other individuals resulting in copulatory behaviour, resources including food and ions in the external pool, and substances added to the medium either by the snails or their plant food receive expression in the growth and natality rates of individual Biomphalaria glabrata. The effects of chemical conditioning or of particular chemicals were assayed in both closed and open systems using the principle of the chemostat. The two types of media used were heterotypically conditioned media produced by interactions between the snail, food and the medium and homotypically conditioned media produced by interaction between the snails, the aquatic medium and a pure cellulose food source. The various effects observed on growth and fecundity of juvenile snails has been shown to be a function of numbers, biomass, volume or space, and time are classified. The causes of these effects are also summarized.	Irrelevant	Title and abstract are not relevant with the topic	References removed

105	Meriam Miladi, Khemais Abdellaoui, Amel Ben Hamouda, Iteb Boughattas, Mouna Mhafdhi, Fatma Acheuk, Monia Ben Halima-Kamel	Physiological, histopathological and cellular immune effects of Pergularia tomentosa extract on Locusta migratoria nymphs	2019	Journal of Integrative Agriculture	https://doi.org/10.1016/S2095-3119(19)62704-8	The migratory locust Locusta migratoria (Orthoptera, Acrididae) is one of the most important pests due to its extensive and severe damage to crops in large parts of Africa and Asia. Biodegradable and ecologically natural products such as botanical insecticides are emerging candidates for replacement of usually applied chemical pesticides. The crude methanolic extract of Pergularia tomentosa (PME) was investigated for their toxicity and physiological aspects on L. migratoria nymphs. Results showed that treatment of newly emerged fourth and fifth instar nymphs resulted in significant mortality and significant repellent activity with an LC50 value of 0.18 and 0.38%, respectively, after seven days of treatment. The PME toxicity was also demonstrated by histopathological changes in the alimentary canal resulting in considerable disorganization and severe damage of the caeca and proventriculus structure. The extract induced cellular immune reactions which manifested by a significant decrease in the number of the differential haemocyte counts (prohemocytes and plasmatocytes) and important cell lysis. Data of biochemical analyses showed that the PME reduced the activity of acetylcholinesterase and induced the glutathione S-transferases. The neurotoxic effect was confirmed by the histological alterations in the brain structure, particularly in the neurosecretory cells showing typical signs of cell necrosis.	Irrelevant	Title and abstract are not relevant with the topic	References removed
106	Robert M Ouedraogo, Andrena Kamp, Mark S Goettel, Jacques Brodeur, Michael J Bidochka	Attenuation of fungal infection in thermoregulating Locusta migratoria is accompanied by changes in hemolymph proteins	2002	Journal of Invertebrate Pathology	https://doi.org/10.1016/S0022-2011(02)00117-9	Hemolymph proteins in the locust, Locusta migratoria migratorioides infected with the fungus Metarhizium anisopliae var acridum were analyzed with sodium dodecyl sulfateâ€“polyacrylamide gel electrophoresis (SDSâ€“PAGE). Under conditions that allowed locusts to thermoregulate, 2 proteins, ITB1 (ca. 18kDa) and ITB2 (ca. 13kDa) were induced 48h post inoculation. In contrast, under non-thermoregulating conditions, only 1 band, INTB1 (ca. 18kDa) was induced with similar molecular mass to ITB1. ITB1 and ITB2 were N-terminally sequenced but showed little homology to known proteins. The induction of hemolymphal proteins in infected, thermoregulating locusts and implication in insect immune defence are discussed.	Irrelevant	Title and abstract are not relevant with the topic	References removed
107	K L Quickenden, G R Roemhild	Maternal age and density effects on carbohydrate partitioned to eggs of the grasshopper, Aulocara elliotti	1969	Journal of Insect Physiology	https://doi.org/10.1016/0022-1910(69)90231-5	Mannose, glucose, glycerol, and trehalose were identified by chromatographic means in 1- to 7-day-old eggs of the grasshopper, Aulocara elliotti. Trehalose and â€˜glycogenâ€™ levels, at a time when embryonic development had not progressed beyond the blastoderm stage, were measured in eggs collected from adults reared at three densities throughout the fecund period. Glycogen levels in these eggs increased with maternal age as did egg weight. Parental density had no noticeable effect on glycogen content in eggs. During the last half of the fecund period, trehalose decreased from 57Â·6 to 20Â·2 Î¼g/egg (9Â·46 to 3Â·21 mg/g fresh egg weight) in eggs obtained from adults reared at the highest density. This is probably due to the combined effect of crowding and maternal age. The greater the density, the greater the amount of trehalose that was partitioned to eggs during the first two-thirds of the reproductive period. Maternal age and density effects on trehalose levels partitioned to eggs are discussed in relation to rate of development and a density-stress response mechanism which may be likened to that of vertebrates.	Irrelevant	Title and abstract are not relevant with the topic	References removed

108	Xiao-Meng Xun, Cheng-Hai Yan, Zi-Xuan Yuan, Zhi-Ang Zhang, Richard Ansah Herman, Yan Xu, Qiong-Ying Wu, Jun Wang	60Co-Î³-ray-irradiated edible silkworm (Bombyx mori) pupae-assisted protease digestion: A strategy for obtaining low molecular weight bioactive peptides	2025	Sustainable Chemistry and Pharmacy	https://doi.org/10.1016/j.scp.2025.101941	Bioactive peptides from edible insect protein are a class of peptide compounds with relatively low molecular weight, possessing specific biological activity functions, but their extraction and utilization face challenges. The study aims to explore a novel approach using 60Co-Î³-ray irradiation-assisted enzymatic hydrolysis of silkworm pupae protein to obtain bioactive peptides. The silkworm pupae were irradiated with 60Co-Î³-ray at a dose point of 10Â kGy, and then the protein was extracted and subsequently hydrolyzed by alkaline protease, and the irradiated silkworm pupae protein improved solubility and hydrolysis degree. The proportion of Î±-helix in the secondary structure of the irradiated silkworm pupae protein increases, indicating that irradiation can enhance its functional properties by altering the intrinsic structure. Through irradiation-assisted enzymatic hydrolysis, The percentage of low molecular weight peptides (180â€“500Â Da) prepared by irradiation-coupled enzymatic hydrolysis increased from 32.04Â Â±Â 0.97% to 52.77Â Â±Â 0.72%, and the percentage of low molecular weight peptides (0.5â€“1Â kDa) increased from 19.65Â Â±Â 0.52% to 24.75Â Â±Â 0.79%. Activity-guided isolation and identification reveal a greater diversity of peptides with antioxidant, hypoglycemic, and hypotensive activities in the irradiated group, including novel peptides such as QASSGTPATLR, LLPR, FKVPN, and HHFP that exhibit strong scavenging activity against DPPH radicals, Î±-glucosidase inhibition, Fe2+ chelation, and angiotensin-converting enzyme (ACE) inhibition. Molecular docking simulations shown that the irradiated peptides have enhanced affinity for target enzymes, suggesting that the 60Co-Î³-ray irradiation-assisted enzymatic hydrolysis technique can effectively improve peptide activity. This study provides a theoretical basis for irradiation-assisted enzymatic degradation of edible insect proteins to obtain low molecular weight bioactive peptides.	Relevant	Title and abstract are relevant to the topic	References are used for the full review stage
109	G Anjani, R N Pratiwi, N F Fathimatuzzahrah, R A Kusuma, D N Afifah	Protein quality and physical characteristic of wood grasshopper (Melanoplus cinereus) hydrolysate flour	2023	Food Research	10.26656/fr.2017.7(S3).13	Indonesia is a tropical country with high biodiversity including insects. Insects are referred to as a good source of protein to overcome various nutritional problems. Wood grasshoppers (Melanoplus cinereus) have been consumed for a long time as a source of protein with limited digestibility due to its high chitin content. In this research, wood grasshoppers flour was added with bromelain enzyme with various concentrations 0%, 4%, 5%, 6% (w/v) to produce hydrolyzed flour in order to improve its protein quality. The manufacture of wood grasshoppers hydrolyzate flour was carried out by first dissolving the flour with water, then adjusting it at pH 7. Then, various concentrations of the bromelain enzyme and inucbated 7 hrs at 55Â°C. The final product obtained was the hydrolyzed flour in freeze-dried form. These flour were analyzed for their proximate analysis, protein quality (soluble protein, protein digestibility, amino acids total), antioxidant activity and physical properties (pH, color quantification). All these results were compared to obtain the best flour quality. The protein digestibility and color quality improved by the increasing bromelain concentration. However, soluble protein, amino acid total, and pH of wood grasshopperâ€™s hydrolysate flour decreased. Variation of bromelain concentration gave a significant difference on water content, carbohydrates, fats, protein, protein soluble, protein digestibility, antioxidant activity, redness and yellowness. Nonetheless ash content, pH and lightness of these flour were comparable. In conclusion, the best protein digestibility of wood grasshopperâ€™s hydrolysate flour was 6% of bromelain concentration, which was 51.33%. Â© 2023, Rynnye Lyan Resources. All rights reserved.	Relevant	Title and abstract are relevant to the topic	References are used for the full review stage
110	Felicia G Hall, Owen G Jones, Marguerite E O'Haire, Andrea M Liceaga	Functional properties of tropical banded cricket (Gryllodes sigillatus) protein hydrolysates.	2017	Food chemistry	10.1016/j.f[illegible]	Recently, the benefits of entomophagy have been widely discussed. Due to western cultures' reluctance, entomophagy practices are leaning more towards incorporating insects into food products. In this study, whole crickets (Gryllodes sigillatus) were hydrolyzed with alcalase at 0.5, 1.5, and 3.0% (w/w) for 30, 60, and 90min. Degree of hydrolysis (DH), amino acid composition, solubility, emulsion and foaming properties were evaluated. Hydrolysis produced peptides with 26-52% DH compared to the control containing no enzyme (5% DH). Protein solubility of hydrolysates improved (p<0.05) over a range of pH's, exhibiting >30% soluble protein at pH 3 and 7 and 50-90% at alkaline pH, compared with the control. Emulsion activity index ranged from 7 to 32m(2)/g, while foamability ranged from 100 to 155% for all hydrolysates. These improved functional properties demonstrate the potential to develop cricket protein hydrolysates as a source of functional alternative protein in food ingredient formulations.	Relevant	Title and abstract are relevant to the topic	References are used for the full review stage

111	B Purschke, P Meinlschmidt, C Horn, O Rieder, H JÃ¤ger	Improvement of techno-functional properties of edible insect protein from migratory locust by enzymatic hydrolysis	2018	European Food Research and Technology	10.1007/s0	Enzymatic hydrolysis of migratory locust (Locusta migratoria L.) protein flour (MLPF) was investigated as a method to improve the techno-functional properties. Experiments were conducted under variation of the applied proteases (Alcalase, Neutrase, Flavourzyme, Papain) or combinations thereof, enzymeâ€“substrate ratio (0.05â€“1.0% w/w), heat pre-treatment (60â€“80Â Â°C; 15â€“60Â min), and hydrolysis time (0â€“24Â h). Protein degradation was monitored in terms of degree of hydrolysis (DH) and SDS-PAGE. Solubility, emulsifying, foaming and water/oil binding properties of the hydrolysates were determined. In comparison to the control (DH = 5%), hydrolysis resulted in considerably higher DH values up to 42%. SDS-PAGE profiles revealed a steady decrease of bands between 25 and 75Â kDa and an increase of low molecular weight bands (10â€“15Â kDa). However, different heat pre-treatments resulted in impaired hydrolytic cleavage as evidenced by lower DH values. Protein solubility of MLPF hydrolysates was improved over a broad pH range from initially 10â€“22% up to 55% at alkaline conditions. Furthermore, hydrolysis resulted in enhanced emulsifying activity (54%) at pH 7, improved foamability (326%) at pH 3 and advanced oil binding capacity. The results of this study have clearly demonstrated the potential of targeted enzymatic degradation to improve the techno-functionality of migratory locust protein in order to produce tailored insect-based ingredients for the use in food applications. Â© 2017, The Author(s).	Relevant	Title and abstract are relevant to the topic	References are used for the full review stage
112	S Anootthato, N Therdthai, P Ritthiruangdej	Characterization of protein hydrolysate from silkworm pupae (Bombyx mori)	2019	Journal of Food Processing and Preservation	10.1111/jfp	Silkworm pupae (Bombyx mori) were subjected to drying, defatting, and hydrolysis using two enzymes: Alcalase and Neutrase. The degree of hydrolysis by Alcalase (9.61%) was much higher than for Neutrase (2.91%). Enzymatic hydrolysis increased the amount of total amino acid from 9.4 (raw material) to 42.56â€“62.37Â mg/100Â mg with the highest productivity obtained using Alcalase hydrolysis. The molecular weight of the hydrolysate using the Alcalase treatment was less than that using the Neutrase treatment. The antioxidant activity of both protein hydrolysates was higher than for the raw material with the decrease in IC50 (the inhibitory concentration required to inhibit 50% of 1,1-diphenyl-2-picrylhydrazyl radical) and the improvement of ferric reducing antioxidant power. Both enzymatic treatments improved the water solubility of dried and defatted samples. The foaming capacity and stability was improved by Neutrase hydrolysis. In contrast, the emulsifying properties were not obviously improved by either Alcalase hydrolysis or Neutrase hydrolysis. Practical applications: Enzymatic hydrolysis could improve the physicochemical and functional properties of dried silkworm pupae. Protein hydrolysate from Neutrase hydrolysis provided excellent antioxidant activity, water solubility, and foaming properties. Therefore, it could be used as an ingredient for the development of food with high antioxidant activity and foaming characteristics. Protein hydrolysate from Alcalase hydrolysis provided high protein content, total amino acid content, and water solubility index. Therefore, it could be used as the ingredient for protein enrichment in food product development. Â© 2019 Wiley Periodicals, Inc.	Relevant	Title and abstract are relevant to the topic	References are used for the full review stage

113	Y Zhang, J Wang, Z Zhu, X Li, S Sun, W Wang, F A Sadiq	Identification and characterization of two novel antioxidant peptides from silkworm pupae protein hydrolysates	2021	European Food Research and Technology	10.1007/s0	Silkworm pupae are a kind of insect resource food that rich of good quality protein. To identify the peptide with high antioxidant activity from silkworm pupae protein hydrolysates, and provide the basis for the application of silkworm pupae protein hydrolysates and antioxidant peptides in functional foods, silkworm pupae proteins were hydrolysed by a dual-enzyme system consisting of acidic protease and neutral protease. The hydrolysates were purified sequentially by ultrafiltration, gel filtration chromatography and high-performance liquid chromatography (HPLC). The ABTS radical scavenging activity was used to evaluate antioxidant activity. Fractions with high activity were further analyzed by liquid chromatography- tandem mass spectrometry (LCâ€“MS/MS). Two peptides, FKGPACA and SVLGTGC with molecular weights of 692.34 and 635.30Â Da were obtained. To further determine the major active sites of FKGPACA and SVLGTGC, four peptides, FKGP, ACA, SVLG and TGC were artificially synthesized. ACA and TGC had higher ABTS radical scavenging activities than FKGP and SVLG. The main active sites of FKGPACA and SVLGTGC were possibly located in the ACA and TGC fragments, which are related to Cys, Ala or Thr residues. Both FKGPACA and SVLGTGC proved to good antioxidants even after high-temperature thermal processing for 1Â h. After digestion with pepsin, the ABTS radical scavenging activity of FKGPACA was stable, while the ABTS radical scavenging activity of SVLGTGC decreased slightly. After further digestion with pancreatin, the ABTS radical scavenging activities of FKGPACA and SVLGTGC decreased by 10.59% and 43.56%, respectively. After digestion with chymotrypsin, the ABTS radical scavenging activities of FKGPACA and SVLGTGC were stable. The silkworm pupae protein hydrolysates and FKGPACA could be potentially used as natural antioxidants in functional foods. Â© 2020, Springer-Verlag GmbH Germany, part of Springer Nature.	Relevant	Title and abstract are relevant to the topic	References are used for the full review stage
114	Sungwon Yoon, Nathan A K Wong, Minki Chae, Joong-Hyuck Auh	Comparative Characterization of Protein Hydrolysates from Three Edible Insects: Mealworm Larvae, Adult Crickets, and Silkworm Pupae.	2019	Foods (Basel, Switzerland)	10.3390/fo	A comparative characterization of proteins from three edible insects-Tenebrio molitor (mealworm) larvae, Gryllus bimaculatus (cricket), and Bombyx mori (silkworm) pupae-was performed in this study. Proteins were extracted from edible insects and their hydrolysates were prepared through enzymatic hydrolysis with commercial enzymes (Flavourzyme: 12%; Alcalase: 3%). Solubility was significantly higher following enzymatic hydrolysis, while foamability was lower compared to those of the protein control. Angiotensin-converting enzyme was significantly inhibited after enzymatic hydrolysis, especially following Alcalase treatment, with IC(50) values of 0.047, 0.066, and 0.065 mg/mL for G. bimaculatus, T. molitor larvae, and B. mori pupae, respectively. Moreover, the Alcalase-treated group of B. mori pupae and the T. molitor larvae group treated with a mixture of enzymes showed the effective inhibition of Î±-glucosidase activity. The anti-inflammatory activity of the insect hydrolysates was assessed via nitric oxide production from macrophages, and B. mori pupae samples exhibited significant activity regardless of the method of hydrolysis. These results indicate the functional properties of protein and hydrolysates from three species of edible insects, which may be useful in their future exploitation.	Relevant	Title and abstract are relevant to the topic	References are used for the full review stage
115	Ewelina ZieliÅ„ska, Barbara Baraniak, Monika KaraÅ›	Antioxidant and Anti-Inflammatory Activities of Hydrolysates and Peptide Fractions Obtained by Enzymatic Hydrolysis of Selected Heat-Treated Edible Insects.	2017	Nutrients	10.3390/nu	This study investigated the effect of heat treatment of edible insects on antioxidant and anti-inflammatory activities of peptides obtained by in vitro gastrointestinal digestion and absorption process thereof. The antioxidant potential of edible insect hydrolysates was determined as free radical-scavenging activity, ion chelating activity, and reducing power, whereas the anti-inflammatory activity was expressed as lipoxygenase and cyclooxygenase-2 inhibitory activity. The highest antiradical activity against DPPH(â€¢) (2,2-diphenyl-1-picrylhydrazyl radical) was noted for a peptide fraction from baked cricket Gryllodes sigillatus hydrolysate (IC(50) value 10.9 Âµg/mL) and that against ABTS(â€¢+) (2,2'-azino-bis(3-ethylbenzothiazoline-6-sulfonic acid) radical) was the highest for raw mealworm Tenebrio molitor hydrolysate (inhibitory concentration (IC(50) value) 5.3 Âµg/mL). The peptides obtained from boiled locust Schistocerca gregaria hydrolysate showed the highest Fe(2+) chelation ability (IC(50) value 2.57 Âµg/mL); furthermore, the highest reducing power was observed for raw G. sigillatus hydrolysate (0.771). The peptide fraction from a protein preparation from the locust S. gregaria exhibited the most significant lipoxygenase and cyclooxygenase-2 inhibitory activity (IC(50) value 3.13 Âµg/mL and 5.05 Âµg/mL, respectively).	Relevant	Title and abstract are relevant to the topic	References are used for the full review stage

116	Shavinder Singh, Hina F. Bhat, Sunil Kumar, Aunzar B. Lone, Rana Muhammad Aadil, Abderrahmane AÃ¯t-Kaddour, Abdo Hassoun, Charalampos Proestos, Zuhaib F. Bhat	Ultrasonication and microwave pre-treated locust protein hydrolysates enhanced the storage stability of meat emulsion	2023	Ultrasonics Sonochemistry	10.1016/j.u	Locust protein hydrolysates (LoProHs) pre-processed with microwave and ultrasonication were developed and evaluated for their potential for enhancing the quality of the stored meat emulsion (MEmul). Locust protein (LoPro) samples pre-processed with ultrasonication (Ult) or microwave (Mic) or with no treatment (Not) were hydrolysed with alcalase enzyme (3%). The microwave pre-processed (Mic-LoProHs) and ultrasonicated (Ult-LoProHs) hydrolysates showed significantly (P < 0.05) higher antioxidant [FRAP (ferric reducing antioxidant power) and ABTS and DPPH radical scavenging activities] and antimicrobial [minimum inhibitory concentration (MIC) and inhibitory halos (mm)] potential. The MEmul samples incorporated with Mic-LoProHs and Ult-LoProHs at the maximum level of 1.5% exhibited significantly (P < 0.05) improved results for all the quality parameters such as antioxidant potential (FRAP, ABTS and DPPH), protein oxidation (total carbonyl content), lipid stability, and microbial quality during refrigerated storage (4 Â± 1 Â°C) of two-weeks compared to the control MEmul without any LoProHs. A positive (P < 0.05) impact of the LoProHs was found on the sensory quality of MEmul samples after one week of storage. The digestion simulation improved (P < 0.05) the antioxidant potential of the MEmul samples.	Relevant	Title and abstract are relevant to the topic	References are used for the full review stage
117	Francielle Miranda de Matos, Paula Kern Novelli, Ruann Janser Soares de Castro	Enzymatic hydrolysis of black cricket (Gryllus assimilis) proteins positively affects their antioxidant properties.	2021	Journal of food science	10.1111/17	The development of innovative ingredients through biotechnological routes has established insect proteins as an emerging source of bioactive peptides. The current study aimed to evaluate the antioxidant properties of black cricket (Gryllus assimilis) protein hydrolysates produced using the proteases Flavourzyme(TM) 500L, Alcalase(TM) 2.4L, and Neutrase(TM) 0.8L, either individually or in binary/ternary combinations. The enzymatic hydrolysis promoted an increase of approximately 160% in total antioxidant capacity and 93% in the ferric reducing antioxidant power. The isolated use of the enzyme Flavourzyme(TM) 500L showed the most prominent positive effect on the antioxidant properties, presenting an IC(50) value of 455 and 71Â Âµg/mL for DPPH and ABTS radicals scavenging activities, respectively. This sample was composed mainly of small peptides (MW <Â 3Â kDa), in which the antioxidant properties increased after fractionation by ultrafiltration. Gel electrophoresis analysis showed protein hydrolysates composed mainly of polypeptide chains with a mass of less than 14Â kDa. Finally, the enzymatic treatment proved to be an efficient process to improve the antioxidant properties of black cricket proteins, increasing the possibility of applying these hydrolysates as bioactive ingredients in food or nutraceutical products. PRACTICAL APPLICATION: Insects represent an alternative source of proteins. Their modification through hydrolysis allows for the acquisition of compounds with great potential in industrial applications, such as functional ingredients or for nutraceutical purposes. The use of our experimental design proved to be an adequate tool for defining the best process conditions required for increasing the attainment of biologically active compounds.	Relevant	Title and abstract are relevant to the topic	References are used for the full review stage
118	Felicia Hall, Philip E. Johnson, Andrea Liceaga	Effect of enzymatic hydrolysis on bioactive properties and allergenicity of cricket (Gryllodes sigillatus) protein	2018	Food Chemistry	10.1016/j.f	Food-derived bioactive peptides have gained attention for their role in preventing chronic diseases. Edible insects are viable sources of bioactive peptides owing to their high protein content and sustainable production. In this study, whole crickets (Gryllodes sigillatus) were alcalase-hydrolyzed to a degree of hydrolysis (DH) ranging from 15 to 85%. Antioxidant activity, angiotensin converting enzyme (ACE), and dipeptidyl peptidase-4 (DPP-IV)- inhibition of the cricket protein hydrolysates (CPH) were evaluated before and after simulated gastrointestinal digestion (SGD). Antioxidant activity was similar among CPH, whereas ACE and DPP-IV inhibition was greater (p < 0.05) in CPH with 60â€“85% DH. Bioactivity improved after SGD. CPH allergenicity was evaluated using human shrimp-allergic sera. All sera positively reacted to tropomyosin in the unhydrolyzed cricket and CPH with 15â€“50% DH, whereas 60â€“85% DH showed no reactivity. In conclusion, CPH (60â€“85% DH) had the greatest bioactive potential and lowest reactivity to tropomyosin, compared with other CPH and the unhydrolyzed control.	Relevant	Title and abstract are relevant to the topic	References are used for the full review stage

Relevant =	45
Irrelevant =	73
Total =	118
