# Supplementary material for: Enzymatic Hydrolysis Methods of Insect Orthoptera Protein: A Systematic Review
Source: Int J Food Sci. 2026 Apr 24;2026:9091997. doi: 10.1155/ijfo/9091997 (PMC13108587; doi:10.1155/ijfo/9091997)
Supplement: Supplementary file 4 — Supporting Information 4 Screening results are based on full text review. [file IJFO-2026-9091997-s004.pdf]

Supplementary Material 4
Screening result by full text review

No.	Authors	Title	Year	Source	DOI	Population	Intervention	Comparison	Outcome	Data availability	Full text availability	Inclusion or Exclusion Criteria	Reason	Decision
1	M Maciejewska, A DÄ…browska, M Cano-Lamadrid	Sustainable Protein Sources: Functional Analysis of Tenebrio molitor Hydrolysates and Attitudes of Consumers in Poland and Spain Toward Insect-Based Foods	2025	Foods	10.3390/foods14020333	Edible insect (mealworm)	Insect processing using enzymatic hydrolysis process	duration of hydrolysis	Determination of Antioxidant Activity, Degree of hydrolysis	Yes, available	Yes, available	Exclusion Criteria	Types of insects not included in the Orthoptera order	References removed
2	Suttida Chukiatsiri, Nattakarn Wongsrangsap, Pichamon Kiatwuthinon, Wannarat Phonphoem	Purification and identification of novel antioxidant peptides derived from Bombyx mori pupae hydrolysates	2024	Biochemistry and Biophysics Reports	https://doi.org/10.1016/j.bbrep.2024.101707	Edible insect (Bombyx mori pupae)	Insect processing using enzymatic hydrolysis process	types of protease enzymes	novel antioxidant peptides	Yes, available	Yes, available	Exclusion Criteria	Types of insects not included in the Orthoptera order	References removed
3	L J H Sweers, M Mishyna, R M Boom, V Fogliano, J K Keppler, C M M Lakemond	Microfiltration for effective microbiological decontamination of edible insects â€“ Protein hydrolysis, aggregation and pH are critical for protein recovery	2023	Food and Bioproducts Processing	https://doi.org/10.1016/j.fbp.2023.08.002	Edible insect [Lesser mealworms (Alphitobius diaperinus) and house crickets (Acheta domesticus)]	Microfiltration method	type of insect, ph condition	Protein recovery, Protein solubility, Microfiltration membrane performance, Microbiological analysis , Lipid distribution	Yes, available	Yes, available	Exclusion Criteria	Irrelevant intervention methods	References removed

4	Francielle Miranda de Matos, JosÃ© Thalles Jocelino Gomes de Lacerda, Giovanna Zanetti, Ruann Janser Soares de Castro	Production of black cricket protein hydrolysates with Î±-amylase, Î±-glucosidase and angiotensin I-converting enzyme inhibitory activities using a mixture of proteases	2022	Biocatalysis and Agricultural Biotechnology	https://doi.org/10.1016/j.bcab.2022.102276	Edible insect (black cricket)	Insect processing using enzymatic hydrolysis process	types of protease enzymes	Determination of the in vitro antidiabetic properties, Angiotensin converting enzyme (ACE) inhibitory activity, Electrophoretic profile, Mass spectrometry analysis	Yes, available	Yes, available	Inclusion Criteria	Included/ Eligible	References are continued to the data extraction stage
5	Annalaura Brai, Claudia Immacolata Trivisani, Chiara Vagaggini, Roberto Stella, Roberto Angeletti, Giulia Iovenitti, Valeria Francardi, Elena Dreassi	Proteins from Tenebrio molitor: An interesting functional ingredient and a source of ACE inhibitory peptides	2022	Food Chemistry	https://doi.org/10.1016/j.foodchem.2022.133409	Edible insect (mealworm)	Enzimatic hydrolisis by simulted gastric fluids: pepsin, trypsin, Chymotrypsin	Fractionation of extracts after enzymatic hydrolysis	ACE inhibitory activity assay, In silico analysis, Synthesis of peptides, ACE inhibitory activity of peptides, Quantification of YAN in the supernatant and pellet fractions, LC-HRMS/MS analysis	Yes, available	Yes, available	Exclusion Criteria	Types of insects not included in the Orthoptera order	References removed
6	Milan Dhakal, Varongsiri Kemsawasd, Kanyawee Whanmek, Wimonphan Chathiran, Saranya Intawong, Warangkana Srichamnong, Uthaiwan Suttisansanee, Suwapat Kittibunchakul	Physicochemical characteristics, volatile components and bioactivities of fermented seasoning sauce produced from cricket (Acheta domesticus) meal	2025	Future Foods	https://doi.org/10.1016/j.fufo.2024.100505	Edible insect (House cricket meal)	Accelerated fermentation of cricket flour with enzymes	Types of bacteria	degree of hydrolysis, volatile profile analysis, in vitro investigation of bioactivities	Yes, available	Yes, available	Exclusion Criteria	Improper comparasion or research design	References removed

7	LetÃ-cia Nunes da Cruz, Liliana de Oliveira Rocha, Ruann Janser Soares de Castro	Submerged fermentation using Aspergillus tubingensis as an efficient strategy to obtain antioxidant	2023	Food and Humanity	https://doi.org/10.1016/j.foohum.2023.08.012	Edible insect (black cricket)	submerged fermentative process	-	antioxidant	No	No	Exclusion Criteria	Incomplete data and fulltext	References removed
8	Lorenzo Nissen, Seyedeh Parya Samaei, Elena Babini, Andrea Gianotti	Gluten free sourdough bread enriched with cricket flour for protein fortification: Antioxidant	2020	Food Chemistry	https://doi.org/10.1016/j.foodchem.2020.127410	Edible insect (cricket flour)	Fermented bread enriched with cricket flour	Type of bacteria, fermentation time	Protein characterization, volatile profile analysis, Antioxidant activity assays	Yes, available	Yes, available	Exclusion Criteria	Irrelevant intervention methods	References removed
9	Jia-hao Xu, Shan Xiao, Ji-hui Wang, Bo Wang, Yan-xue Cai, Wen-feng Hu	Comparative study of the effects of ultrasound-assisted alkaline extraction on black soldier fly (Hermetia illucens) larvae protein: Nutritional,	2023	Ultrasonics Sonochemistry	https://doi.org/10.1016/j.ultsonch.2023.106662	Edible insect (black soldier fly)	insect isolation by ultrasound-assisted alkaline extraction	use of ultrasound and without ultrasound	nutritional value, structural characteristics, in vitro protein digestibility, and techno-functional properties	Yes, available	Yes, available	Exclusion Criteria	Irrelevant intervention methods	References removed
10	Laura Jenet Montiel-Aguilar, Jorge Ariel Torres-Castillo, RocÃ-o RodrÃ-guez-Servin, Adiel Berenice LÃ³pez-Flores, VÃ-ctor Eustorgio Aguirre-Arzola, Gerardo MÃ©ndez-Zamora, Sugey Ramona Sinagawa-GarcÃ-a	Nutraceutical effects of bioactive peptides obtained from Pterophylla beltrani (Bolivar & Bolivar) protein isolates	2020	Journal of Asia-Pacific Entomology	10.1016/j.aspen.2020.06.006	Edible insect (Adults of P. beltrani)	Enzimatic hydrolisis by simulted gastric fluids: pepsin, trypsin, Chymotrypsin	Ultrafiltration membrane size	Protein quantification assay, Angiotensin-Converting Enzyme (ACE) inhibitory activity assay, α-Amylase inhibitory activity assay, Antioxidant activity by ABTS assay, bioactive peptides	Yes, available	Yes, available	Inclusion Criteria	Included/ Eligible	References are continued to the data extraction stage

11	Suwapat Kittibunchakul, Kanyawee Whanmek, Chalat Santivarangkna	Physicochemical, microbiological and nutritional quality of fermented cricket (Acheta domesticus) paste	2023	LWT	https://doi.org/10.1016/j.lwt.2023.115444	Edible insect (Acheta domesticus flour)	Cricket processing uses fermentation processes	Duration of fermentation	Physicochemical analysis , Protease activity, Trichloroacetic acid (TCA)-soluble peptide content, Degree of hydrolysis (DH), Microbiological analysis, Nutritional analysis	Yes, available	Yes, available	Exclusion Criteria	Irrelevant intervention methods	References removed
12	Ratasark Summart, Sumeth Imsoonthornruksa, Jirawat Yongsawatdigul, Mariena Ketudat-Cairns, Natteewan Udomsil	Characterization and molecular docking of tetrapeptides with cellular antioxidant and ACE inhibitory properties from cricket (Acheta domesticus) protein	2024	Heliyon	https://doi.org/10.1016/j.heliyon.2024.e35156	Edible insect (cricket)	Insect processing using enzymatic hydrolysis process	Types of antioxidant testing	Cellular antioxidant and ACE inhibitory properties	Yes, available	Yes, available	Inclusion Criteria	Included/ Eligible	References are continued to the data extraction stage
13	Abir Boukil, VÃ©ronique Perreault, Julien Chamberland, Samir Mezdour, Yves Pouliot, Alain Doyen	High Hydrostatic Pressure-Assisted Enzymatic Hydrolysis Affect Mealworm Allergenic Proteins.	2020	Molecules (Basel, Switzerland)	10.3390/molecules25112685	Edible insect (mealworm)	Insect processing using enzymatic hydrolysis process	Types of enzymes, variation of High Hydrostatic Pressure-Assisted Enzymatic Hydrolysis condition	Determination of the Degree of Hydrolysis, Digestion Profiles of Mealworm Proteins, Protein Identification by Mass Spectrometry,	Yes, available	Yes, available	Exclusion Criteria	Types of insects not included in the Orthoptera order	References removed

14	Aunzar B Lone, Hina F Bhat, Sunil Kumar, Mehnaza Manzoor, Abdo Hassoun, Abderrahmane AÃ¯t-Kaddour, Tanyaradzwa E Mungure, Rana Muhammad Aadil, Zuhaib F Bhat	Improving microbial and lipid oxidative stability of cheddar cheese using cricket protein hydrolysates pre-treated with microwave and ultrasonication	2023	Food Chemistry	https://doi.org/10.1016/j.foodchem.2023.136350	Edible insect (cricket)	Insect processing using enzymatic hydrolysis process	There are microwave and ultrasonication modification treatments in the hydrolysis process.	In vitro gastrointestinal digestion, antioxidant activity, microbiological analysis, sensory characteristics, lipid stability, protein oxidation and physicochemical parameters	Yes, available	Yes, available	Inclusion Criteria	Included/ Eligible	References are continued to the data extraction stage
15	Carlos I Rivas-Vela, Eduardo CastaÃ±o-Tostado, Anaberta Cardador-MartÃnez, Silvia L Amaya-Llano, Gustavo A Castillo-Herrera	Subcritical water hydrolysis for the obtention of bioactive peptides from a grasshopper Sphenarium purpurascens protein concentrate	2023	The Journal of Supercritical Fluids	https://doi.org/10.1016/j.supflu.2023.105893	Edible insect (grasshopper)	The effect of adding sodium bicarbonate(SB) and citric acid(CA) in the subcritical water (SW) hydrolysis process	temperature and pressure variations	degree of hydrolysis(DH), molecular distribution, antioxidant activity and Angiotensin I-converting enzyme(ACE) inhibitory activity were evaluate	No	No	Exclusion Criteria	Incomplete data and fulltext	References removed
16	Giulia Leni, Augusta Caligiani, Stefano Sforza	Killing method affects the browning and the quality of the protein fraction of Black Soldier Fly (Hermetia illucens) prepupae: a metabolomics and proteomic insight	2019	Food Research International	https://doi.org/10.1016/j.foodres.2018.08.021	Edible insect (Black Soldier Fly)	Effect of blanching or freezing as killing methods on the susceptibility of insect proteins to enzymatic proteolysis	Differences in insect killing methods by freezing and blanching	Amino acids determination, SDS PAGE, Protein identification by high resolution mass spectrometry, Degree of hydrolysis	Yes, available	Yes, available	Exclusion Criteria	Types of insects not included in the Orthoptera order	References removed
17	Yongli Jiang, Qi Tian, Chongyang Chen, Yun Deng, Xiaosong Hu, Yunjie Yi	Impact of salting-in/out assisted extraction on rheological, biological, and digestive, and proteomic	2024	International Journal of Biological Macromolecules	https://doi.org/10.1016/j.ijbiomac.2024.137044	Edible insect (mealworm)	-	-	-	No	No	Exclusion Criteria	Incomplete data and fulltext	References removed

18	Ha-Seong Cho, Ju-Hwi Park, Ibukunoluwa Fola Olawuyi, Ju-Ock Nam, Won-Young Lee	Optimization of ultrasound-assisted enzymatic hydrolysis Zophobas morio protein and its protective effects against H2O2-	2025	International Journal of Biological Macromolecules	https://doi.org/10.1016/j.ijbiomac.2025.140111	Edible insect (Zophobas morio)	-	-	-	No	No	Exclusion Criteria	Incomplete data and fulltext	References removed
19	Benjamin Kumah Mintah, Ronghai He, Mokhtar Dabbour, Jiahui Xiang, Akwasi Akomeah Agyekum, Haile Ma	Techno-functional attribute and antioxidative capacity of edible insect protein preparations and hydrolysates thereof: Effect of	2019	Ultrasonics Sonochemistry	https://doi.org/10.1016/j.ultsonch.2019.104676	Edible insect (H. illucens larvae)	Insect processing using enzymatic hydrolysis process and ultrasonication	variation of ultrasonication conditions	Determination of techno-functional attributes, Quantification of antioxidative action, Surface hydrophobicity	Yes, available	Yes, available	Exclusion Criteria	Types of insects not included in the Orthoptera order	References removed
20	Francielle Miranda de Matos, Gabriela Boscariol Rasera, Ruann Janser Soares de Castro	Multifunctional properties of peptides derived from black cricket (Gryllus assimilis) and effects of in vitro digestion simulation on their bioactivities	2024	Food Research International	https://doi.org/10.1016/j.foodres.2024.115134	Edible insect (black cricket)	Insect processing using enzymatic hydrolysis process	types of protease enzymes	Determination of antioxidant properties, Determination of antidiabetic properties, Electrophoretic profile, Mass spectrometry analysis	Yes, available	Yes, available	Inclusion Criteria	Included/ Eligible	References are continued to the data extraction stage
21	Teresa Gonzalez-de la Rosa, Sergio Montserrat-de la Paz, Fernando Rivero-Pino	Production, characterisation, and biological properties of Tenebrio molitor-derived oligopeptides	2024	Food Chemistry	https://doi.org/10.1016/j.foodchem.2024.139400	Edible insect (Tenebrio molitor flour)	Insect processing using enzymatic hydrolysis process	Types of enzymes	Chemical characterisation, Antioxidant activity, Anti-inflammatory activity , Peptidome profile, In silico analysis of the selected peptides	Yes, available	Yes, available	Exclusion Criteria	Types of insects not included in the Orthoptera order	References removed
22	Jiao Tan, Jing Yang, Xinyi Zhou, Ahmed Mahmoud Hamdy, Xilu Zhang, Huayi Suo, Yu Zhang, Ning Li, Jiajia Song	Tenebrio molitor Proteins-Derived DPP-4 Inhibitory Peptides: Preparation, Identification, and Molecular Binding Mechanism.	2022	Foods (Basel, Switzerland)	10.3390/foods11223626	Edible insect (Tenebrio molitor flour)	Insect processing using enzymatic hydrolysis process	Types of enzymes	Degree of Hydrolysis (DH) Assay, Structural Characterization, Peptide Identification, Molecular Docking	Yes, available	Yes, available	Exclusion Criteria	Types of insects not included in the Orthoptera order	References removed

23	Kora Kassandra Grossmann, Michael Merz, Daniel Appel, Maria Monteiro De Araujo, Lutz Fischer	New insights into the flavoring potential of cricket (Acheta domesticus) and mealworm (Tenebrio molitor) protein hydrolysates and their Maillard products	2021	Food Chemistry	https://doi.org/10.1016/j.foodchem.2021.130336	Edible insect [cricket (Acheta domesticus) and mealworm (Tenebrio molitor)]	Insect processing using enzymatic hydrolysis process	types of insects, types of enzymes	degree of hydrolysis, enzyme activities, determination of the free amino acids of the insect proteins processed, Gas chromatography–olfactometry (GC-O), Sensory evaluation	Yes, available	Yes, available	Inclusion Criteria	Included/ Eligible	References are continued to the data extraction stage
24	Daniel E Garcia-Valle, Madai. LÃ³pez-Silva, Graciela. Santos-MartÃ-nez, Virginia. HernÃ¡ndez-PÃ©rez, Juan JosÃ©. Figueroa-GonzÃ¡lez	Chemical, structural characterization and in vitro protein digestibility of cicada (Cicadidae) flour	2024	Journal of Food Composition and Analysis	https://doi.org/10.1016/j.jfca.2024.106454	Edible insect (Cicadas (Cicadidae) and grasshoppers)	processing raw Cicadidae into flour	variation of two types of insect samples	In vitro protein digestibility, rotein solubility and accessible thiols	No	No	Exclusion Criteria	Misaligned study outcome with the review focus	References removed
25	C. Azagoh, F. Ducept, R. Garcia, L. Rakotozafy, M. E. Cuvelier, S. Keller, R. Lewandowski, S. Mezdour	Extraction and physicochemical characterization of Tenebrio molitor proteins	2016	Food Research International	10.1016/j.foodres.2016.06.010	Edible insect (Tenebrio molitor)	extracted by solubilisation at an alkaline pH	sample conditions (larvae, flour, deffated)	Molecular weight, solubility, Amino acid content, Determination of fat, water, protein content	Yes, available	Yes, available	Exclusion Criteria	Misaligned study outcome with the review focus	References removed
26	Aysen Bas, Sedef Nehir El	Nutritional evaluation of biscuits enriched with cricket flour (Acheta domesticus)	2022	International Journal of Gastronomy and Food Science	https://doi.org/10.1016/j.ijgfs.2022.100583	Edible insect (Acheta domesticus)	-	-	-	No	No	Exclusion Criteria	Incomplete data and fulltext	References removed

27	Legesse Shiferaw Chewaka, Chan Soon Park, Youn-Soo Cha, Kebede Taye Desta, Bo-Ram Park	Enzymatic Hydrolysis of Tenebrio molitor (Mealworm) Using Nuruk Extract Concentrate and an Evaluation of Its Nutritional, Functional, and Sensory Properties.	2023	Foods (Basel, Switzerland)	10.3390/foods12112188	Edible insect (Tenebrio molitor)	Insect processing using enzymatic hydrolysis process	different types of protease enzymes	Degree of Hydrolysis, Total Soluble Solid and Hydrolysis Yield, Molecular Weight Distribution, Nutritional Composition of MW and Its Hydrolysates, Antioxidant Activity, Angiotensin Converting Enzyme Inhibitory Activity, Sensory Properties	Yes, available	Yes, available	Exclusion Criteria	Types of insects not included in the Orthoptera order	References removed
28	Victor M Villasenor, Jhony Navat Enriquez-Vara, Judith E UrÃ-as-Silva, Eugenia del Carmen Lugo-Cervantes, Diego A Luna-Vital, Luis Mojica	Mexican grasshopper (Sphenarium purpurascens) as source of high protein flour: Techno-functional characterization, and in silico and in vitro biological potential	2022	Food Research International	https://doi.org/10.1016/j.foodres.2022.112048	Edible insect (grasshopper)	Enzimatic hydrolisis by simulated gastrointestinal digestion	Autoclaving temperature	Inflammation markers inhibition (NOS and COX-2 inhibitor), Antioxidant potential of Mexican grasshopper protein hydrolysates, Peptides characterization, sequence identification screening Tests)	Yes, available	Yes, available	Inclusion Criteria	Included/ Eligible	References are continued to the data extraction stage
29	S Lee, Y.-S. Choi, K Jo, T.-K. Kim, H I Yong, S Jung	Quality characteristics and protein digestibility of Protaetia brevitarsis larvae	2020	Journal of Animal Science and Technology	10.5187/jast.2020.62.5.741	Edible insect (P. brevitarsis larvae)	Digestion process of Protaetia brevitarsis larvae flour	insect flour defatted and not deffated	In vitro digestion, SDS-PAGE, Alpha (α)-amino group content, Hydrolysis rate	Yes, available	Yes, available	Exclusion Criteria	Improper comparasion or research design	References removed

30	N Munoz-Seijas, H Fernandes, B FernÃ¡ndez, J M DomÃ-nguez, J M Salgado	Eco-friendly technologies for obtaining antioxidant compounds and protein hydrolysates from edible insect Tenebrio molitor beetles	2025	Food Chemistry	10.1016/j.foodchem.2024.141726	Edible insect (Tenebrio molitor)	Insect processing using enzymatic hydrolysis process	variation of pretreatment by ultrasonication, microwave, thermal extraction, and CO2-assisted extraction	Antioxidant activity, Total amino acids, Proximate composition, Phenolic compounds, flavonoids and ortho-diphenols contents	Yes, available	Yes, available	Exclusion Criteria	Types of insects not included in the Orthoptera order	References removed
31	M J Bidochka, G G Khachatourians	Protein hydrolysis in grasshopper cuticles by entomopathogenic fungal extracellular proteases	1994	Journal of Invertebrate Pathology	10.1006/jipa.1994.1002	Edible insect (grasshopper)	Addition of phenylmethylsulfonyl fluoride (PMSF), a protease inhibitor	Treatment of the various cuticles	Molecular-weight byt wo-dimensional (2D) gel electrophoresis	Yes, available	Yes, available	Exclusion Criteria	Misaligned study outcome with the review focus	References removed
32	Y Chen, J Zhao, W Zhang, T Zhao, Q Zhang, G Mao, W Feng, Q Li, L Yang, X Wu	Purification of novel polypeptides from bee pupae and their immunomodulatory activity in vivo and in vitro	2022	Journal of Insects as Food and Feed	10.3920/JIFF2021.0190	Edible insect (bee pupae)	hydrolysis with alkaline protease	Two purified polypeptide components (BPP-21 and BPP-22)	body weight growth rate, organ index, macrophage phagocytosis, delayed-type hypersensitivity reaction, cytokine level (interleukin (IL)-2 and interferon (IFN)-γ), immunoglobulin (Ig) levels (IgA, IgG, and IgM), and routine blood indexes in cyclophosphamide-treated immunosuppressed mice (P<0.01)	No	No	Exclusion Criteria	Incomplete data and fulltext	References removed

33	Su-Hyeon Pyo, Chae-Ryun Moon, So-Won Park, Ji-yu Choi, Jong-Dae Park, Jung Min Sung, Eun-Ji Choi, Yang-Ju Son	Quality and staling characteristics of white bread fortified with lysozyme-hydrolyzed mealworm powder (Tenebrio molitor L.)	2024	Current Research in Food Science	https://doi.org/10.1016/j.crfs.2024.100685	Edible insect (mealworm powder (Tenebrio molitor L.))	Hydrolysis of chitin from T. molitor powder using lysozyme	Duration of hydrolysis, buffer of hydrolysis	Determination of the enzymatic hydrolysis degree of mealworm chitin, physical properties of breads, Crumb structure image analysis, Bread color analysis, Quality parameters of bread shelf-life , Sensory analysis, antioxidant activity	Yes, available	Yes, available	Exclusion Criteria	Misaligned study outcome with the review focus	References removed
34	Shubam Singh, Hina F Bhat, Sunil Kumar, Mehnaza Manzoor, Aunzar B Lone, Pawan Kumar Verma, Rana Muhammad Aadil, Konstadina Papastavropoulou, Charalampos Proestos, Zuhaib F Bhat	Locust protein hydrolysates have the potential to enhance the storage stability of cheese	2023	Current Research in Food Science	https://doi.org/10.1016/j.crfs.2023.100561	Edible insect (Locust)	Insect processing using enzymatic hydrolysis process	Addition of locust protein hydrolysate as a stabilizer in cheese making	Antioxidant activity, digestion simulation, microbiological evaluation, sensory analysis	Yes, available	Yes, available	Inclusion Criteria	Included/ Eligible	References are continued to the data extraction stage
35	Xiao-Meng Xun, Cheng-Hai Yan, Zi-Xuan Yuan, Zhi-Ang Zhang, Richard Ansah Herman, Yan Xu, Qiong-Ying Wu, Jun Wang	60Co-Î^{3}-ray-irradiated edible silkworm (Bombyx mori) pupae-assisted protease digestion: A strategy for obtaining low molecular weight	2025	Sustainable Chemistry and Pharmacy	https://doi.org/10.1016/j.scp.2025.101941	edible insect (silkworm) pupae	Insect processing using enzymatic hydrolysis process	No irradiation and with irradiation	hydrolysis degree, molecular weight peptides, Bioactive peptides	Yes, available	Yes, available	Exclusion Criteria	Types of insects not included in the Orthoptera order	References removed

36	G Anjani, R N Pratiwi, N F Fathimatuzzahrah , R A Kusuma, D N Afifah	Protein quality and physical characteristic of wood grasshopper (Melanoplus cinereus) hydrolysate flour	2023	Food Research	10.26656/fr.2017.7(S3).13	Edible insect (grasshopper)	Insect processing using enzymatic hydrolysis process	Differences in bromelain enzyme concentration	Proximate analysis, Soluble protein, Amino acid profile, Protein digestibility, pH, Color	Yes, available	Yes, available	Inclusion Criteria	Included/ Eligible	References are continued to the data extraction stage
37	Felicia G Hall, Owen G Jones, Marguerite E O'Haire, Andrea M Liceaga	Functional properties of tropical banded cricket (Gryllodes sigillatus) protein hydrolysates.	2017	Food chemistry	10.1016/j.f	Edible insect (cricket)	Insect processing using enzymatic hydrolysis process	Alkalase enzyme concentration and hydrolysis duration	Degree of Hydrolysis, Amino acid analysis, SDS PAGE, Protein solubility , Foamability and foam stability (hydrolyzed)	Yes, available	Yes, available	Inclusion Criteria	Included/ Eligible	References are continued to the data extraction stage
38	B Purschke, P Meinlschmidt, C Horn, O Rieder, H JÃ¤ger	Improvement of techno-functional properties of edible insect protein from migratory locust by enzymatic hydrolysis	2018	European Food Research and Technology	10.1007/s0	Edible insect (Locust)	Insect processing using enzymatic hydrolysis process	type of enzyme protease, enzyme concentration, pre-treatment hydrolysis, and hydrolysis duration	Molecular weight distribution, Techno-functiona l properties	Yes, available	Yes, available	Inclusion Criteria	Included/ Eligible	References are continued to the data extraction stage
39	S Anootthato, N Therdthai, P Ritthiruangdej	Characterization of protein hydrolysate from silkworm pupae (Bombyx mori)	2019	Journal of Food Processing and Preservation	10.1111/jfp	Edible insect (silkworm pupae)	Insect processing using enzymatic hydrolysis process	type of enzyme protease and hydrolysis duration	Degree of hydrolysis and protein recovery , ntioxidant activity, Determination of functional properties	Yes, available	Yes, available	Exclusion Criteria	Types of insects not included in the Orthoptera order	References removed

40	Y Zhang, J Wang, Z Zhu, X Li, S Sun, W Wang, F A Sadiq	Identification and characterization of two novel antioxidant peptides from silkworm pupae protein hydrolysates	2021	European Food Research and Technology	10.1007/s0	Edible insect (silkworm pupae)	Insect processing using enzymatic hydrolysis process	The enzymatic hydrolysates were separated into three fractions, UF-I (MW > 5 kDa), UF-II (MW = 3–5 kDa), and UF-III (MW < 3 kDa)	Identification of purified peptides, Determination of ABTS radical scavenging activity	Yes, available	Yes, available	Exclusion Criteria	Types of insects not included in the Orthoptera order	References removed
41	Sungwon Yoon, Nathan A K Wong, Minki Chae, Joong-Hyuck Auh	Comparative Characterization of Protein Hydrolysates from Three Edible Insects: Mealworm Larvae, Adult	2019	Foods (Basel, Switzerland)	10.3390/fo	Three Edible Insects: Mealworm Larvae, Adult Crickets, and Silkworm Pupae	Insect processing using enzymatic hydrolysis process	type of enzyme protease	ACE Inhibitory Activity, α-glucosidase inhibitory, Anti-Inflammatory Activity, Solubility	Yes, available	Yes, available	Inclusion Criteria	Included/ Eligible	References are continued to the data extraction stage
42	Ewelina ZieliÅ„ska, Barbara Baraniak, Monika KaraÅ›	Antioxidant and Anti-Inflammatory Activities of Hydrolysates and Peptide Fractions Obtained by Enzymatic Hydrolysis of Selected Heat-Treated Edible Insects.	2017	Nutrients	10.3390/nu	Three species of insects: mealworms (larvae), locusts Schistocerca gregaria (adult), and crickets Gryllodes sigillatus (adult)	Enzimatic hydrolisis by simulated gastrointestinal digestion	The raw, boiled, and baked insect proteins	DPPH Radical Scavenging Activity Assay, ABTS Radical Scavenging Activity Assay, Determination of Fe 2+ Chelating Activity, Ferric-Reducing Power, Anti-Inflammatory Activity	Yes, available	Yes, available	Inclusion Criteria	Included/ Eligible	References are continued to the data extraction stage

43	Shavinder Singh, Hina F. Bhat, Sunil Kumar, Aunzar B. Lone, Rana Muhammad Aadil, Abderrahmane AÃ¯t-Kaddour, Abdo Hassoun, Charalampos Proestos, Zuhaib F. Bhat	Ultrasonication and microwave pre-treated locust protein hydrolysates enhanced the storage stability of meat emulsion	2023	Ultrasonics Sonochemis try	10.1016/j.u	Edible insect (Locust)	Insect processing using enzymatic hydrolysis process	pre-processed with ultrasonication (Ult) or microwave (Mic) or with no treatment (Not)	Physicochemical parameters, oxidative stability and antioxidant potentia, Microbiological analysis, Sensory analysis	Yes, available	Yes, available	Inclusion Criteria	Included/ Eligible	References are continued to the data extraction stage
44	Francielle Miranda de Matos, Paula Kern Novelli, Ruann Janser Soares de Castro	Enzymatic hydrolysis of black cricket (Gryllus assimilis) proteins positively affects their antioxidant properties.	2021	Journal of food science	10.1111/17	Edible insect (black cricket)	Insect processing using enzymatic hydrolysis process	type of enzyme protease	Effect of hydrolysate concentration on antioxidant properties, Electrophoretic profile of the protein hydrolysates	Yes, available	Yes, available	Inclusion Criteria	Included/ Eligible	References are continued to the data extraction stage
45	Felicia Hall, Philip E. Johnson, Andrea Liceaga	Effect of enzymatic hydrolysis on bioactive properties and allergenicity of cricket (Gryllodes sigillatus) protein	2018	Food Chemistry	10.1016/j.f	Edible insect (cricket)	Enzimatic hydrolisis by simulated gastrointestina l digestion	enzyme concentration and hydrolysis duration	Degree of hydrolysis, Antioxidant capacity, SDS-PAGE and Immunoblotting	Yes, available	Yes, available	Inclusion Criteria	Included/ Eligible	References are continued to the data extraction stage

Recap of results	Sum	Note
Article Included/ Eligible (Inclusion Criteria) =	16	Study learned, proceed to next stage
Types of insects not included in the Orthoptera order (Exclusion Criteria)=	13	
Misaligned study outcome with the review focus (Exclusion Criteria) =	4	
Irrelevant intervention methods (Exclusion Criteria) =	4	
Improper comparasion or research design (Exclusion Criteria) =	2	
Incomplete data and fulltext (Exclusion Criteria) =	6	
Total =	45	
